# Long-Term Efficacy, Safety, and Pharmacokinetics of Drisapersen in Duchenne Muscular Dystrophy: Results from an Open-Label Extension Study

**DOI:** 10.1371/journal.pone.0161955

**Published:** 2016-09-02

**Authors:** Nathalie M. Goemans, Már Tulinius, Marleen van den Hauwe, Anna-Karin Kroksmark, Gunnar Buyse, Rosamund J. Wilson, Judith C. van Deutekom, Sjef J. de Kimpe, Afrodite Lourbakos, Giles Campion

**Affiliations:** 1 University Hospitals Leuven, Leuven, Belgium; 2 University of Gothenburg, Gothenburg, Sweden; 3 Spica Consultants Ltd, Marlborough, United Kingdom; 4 BioMarin Pharmaceutical Inc., Leiden, Netherlands; Universidad de Sevilla, SPAIN

## Abstract

**Background:**

Drisapersen induces exon 51 skipping during dystrophin pre-mRNA splicing and allows synthesis of partially functional dystrophin in Duchenne muscular dystrophy (DMD) patients with amenable mutations.

**Methods:**

This 188-week open-label extension of the dose-escalation study assessed the long-term efficacy, safety, and pharmacokinetics of drisapersen (PRO051/GSK2402968), 6 mg/kg subcutaneously, in 12 DMD subjects. Dosing was once weekly for 72 weeks. All subjects had a planned treatment interruption (weeks 73–80), followed by intermittent dosing (weeks 81–188).

**Results:**

Subjects received a median (range) total dose of 5.93 (5.10 to 6.02) mg/kg drisapersen. After 177 weeks (last efficacy assessment), median (mean [SD]) six-minute walk distance (6MWD) improved by 8 (-24.5 [161]) meters for the 10 subjects able to complete the 6MWD at baseline (mean age [SD]: 9.5 [1.9] years). These statistics include 2 subjects unable to complete the test at later visits and who scored “zero”. When only the 8 ambulant subjects at week 177 were taken into account, a median (mean [SD]) increase of 64 (33 [121]) meters in 6MWD was observed. Of 7 subjects walking ≥330 m at extension baseline, 5 walked farther at week 177. Of 3 subjects walking <330 m, 2 lost ambulation, while 1 declined overall but walked farther at some visits. Over the 188 weeks, the most common adverse events were injection-site reactions, raised urinary α_1_-microglobulin and proteinuria. Dystrophin expression was detected in all muscle biopsies obtained at week 68 or 72.

**Conclusion:**

Drisapersen was generally well tolerated over 188 weeks. Possible renal effects, thrombocytopenia and injection-site reactions warrant continued monitoring. Improvements in the 6MWD at 12 weeks were sustained after 3.4 years of dosing for most patients. For a small, uncontrolled study, the outcomes are encouraging, as natural history studies would anticipate a decline of over 100 meters over a 3-year period in a comparable cohort.

**Trial Registration:**

ClinicalTrials.gov NCT01910649

## Introduction

Duchenne muscular dystrophy (DMD, OMIM 310200) is an X-linked recessive muscle disease with a global incidence of one in 3,500–5,000 newborn boys [1–3]. DMD is typically diagnosed by the age of five years, when deficits in motor function development become apparent, and has a predictable clinical trajectory. Initial development of motor skills is followed by a plateau phase; then progressive muscle function deterioration with age is observed (decline to be expected from 7 years of age), leading to loss of ambulation [4–10]. By their late teens, most patients develop reduced respiratory capacity leading to severe respiratory dysfunction, frequently necessitating ventilator support and reducing quality of life. Untreated, most patients die in their early twenties as a result of respiratory complications or cardiac dysfunction. Interventions such as glucocorticosteroid treatment [11] and ventilator support [12] can delay key milestones of progression, but do not fundamentally alter the course of the disease. Even with state-of-the-art medical care, most patients do not survive beyond their third decade, while no curative treatment is available.

DMD is caused by mutations (mostly deletions of one or more exons) in the *DMD* gene encoding the dystrophin protein [13], which has key structural and signalling functions in skeletal, smooth, and cardiac muscle. The resulting disruption of the transcriptional open-reading frame leads to prematurely aborted dystrophin synthesis. The lack of dystrophin at the myofiber membranes causes progressive myofiber damage and degeneration, and replacement of muscle by adipose and connective tissue. Mutations that maintain the translational-reading frame usually lead to internally truncated yet largely functional dystrophin proteins and are associated with typically much milder Becker muscular dystrophy (BMD) phenotypes [14].

A promising therapeutic strategy involves antisense oligonucleotides inducing specific exon skipping during pre-messenger RNA (mRNA) splicing [15,16]. This strategy aims to correct the reading frame and produces a shortened but functional dystrophin protein in patients with a DMD mutation [17]. Although the functionality of the resulting protein may vary, this could delay or even stop disease progression and may improve function in the remaining muscle [18]. Drisapersen (PRO051/GSK2402968) is a 2′-*O*-methyl phosphorothioate RNA antisense oligonucleotide that induces exon 51 skipping [19]. The skipping of exon 51 impacts the largest subgroup of DMD patients (approximately 13%), including those with deletions of exons 45 to 50, 48 to 50, 50 and 52 [20].

After a clinical study established proof of concept for local intramuscular administration of drisapersen in DMD [21], subcutaneous (sc) drisapersen was administered to 12 male subjects with DMD in an open-label, dose-escalation study, with dose-related novel dystrophin expression [22]. Subjects subsequently entered an extension phase, receiving drisapersen, 6 mg/kg/week sc. Over the first 12 weeks of the extension, treatment was well tolerated without serious adverse events (SAEs) and clinical effects were promising [22]. The current study is an ongoing, open-label extension of the dose-escalation study assessing long-term efficacy, safety, and pharmacokinetics of drisapersen (6 mg/kg sc) in these 12 subjects with DMD. Here we report the results after 188 weeks of follow-up.

## Materials and Methods

The protocol for this trial and the supporting TREND checklist are available as supporting information; see S1 Protocol and S1 Checklist, respectively. All amendments to the original protocol were approved by the local ethics committee.

### Ethical Conduct of the Study

The PRO051-02 study (NCT01910649; DMD114673) was conducted in accordance with the International Conference on Harmonization (ICH) guidance for Good Clinical Practice, the Declaration of Helsinki (2008), the European Directive 2001/20/EC and local regulations.

This two center study was approved by the local independent ethics committees (Medical Ethics Committee of University Hospitals Leuven, Belgium and Regional Ethics Committee of Gothenburg, Sweden) and authorized by the competent authorities (Belgian Federal Agency for Medicines and Health Products and the Swedish Medical Products Agency) of Belgium and Sweden. Written informed consent from parents/guardians and assent (subjects over 12 years of age) was obtained for all subjects prior to any study procedure.

The study was initially sponsored by Prosensa Therapeutics BV (Leiden, Netherlands) and study sponsorship was transferred to GlaxoSmithKline (Brentford, UK) in July 2011.

### Study Population

Male subjects aged 5–16 years with DMD resulting from a mutation correctable by treatment with drisapersen were eligible for inclusion in the original dose-escalation study (EudraCT 2007-004819-54). Inclusion and exclusion criteria, which have been described previously [22], are summarized in the Supporting S1 Methods and S1 Results.

### Study Design

The present study is an ongoing, open-label extension of the Phase I/IIa, open-label dose-escalation study, performed at two centers (University Hospitals Leuven, Belgium, and The Queen Silvia Children’s Hospital, Gothenburg, Sweden), from July 2009 to April 2013.

The initial Phase I/IIa, open-label study (5-week dose-escalation, then 13 weeks off-treatment) aimed to assess the safety, tolerability, pharmacokinetics, clinical and molecular effects of multiple (max 6 mg/kg) abdominal sc doses of drisapersen in 12 subjects with DMD [22]. After completion and following an off-treatment period (of 6–15 months), all subjects were included in the open-label extension study based on the drisapersen safety profile, pharmacokinetic data and effects seen on muscle biopsy. The start of the extension study was defined as “extension baseline” (week 0). The objectives of the extension period were to assess the long-term efficacy, safety, and pharmacokinetics of drisapersen (6 mg/kg sc) in these 12 subjects with DMD. The current analysis reports on the 188 weeks extension cut-off.

### Study Drug and Dosing

Abdominal sc injections of drisapersen (SynCo Bio Partners BV, Amsterdam, Netherlands) [21], 6 mg/kg (maximum 366 mg, capped at 300 mg from week 54), were given once weekly initially. After approximately 9 months of treatment, rotation among other injection sites was recommended.

All subjects had a scheduled interruption of treatment from weeks 73 to 80 inclusive for pharmacokinetic analyses. It was anticipated that this will not compromise efficacy due to retention of drisapersen in muscle and the long half-life of dystrophin. The interruption was planned and documented in a protocol amendment. After this period, a change from continuous to intermittent dosing was made after evaluating safety and pharmacokinetic data. An intermittent-dosing regimen, 6 mg/kg once weekly for 8 weeks followed by 4 weeks off-treatment, was introduced at week 81. At 188 weeks, all subjects had completed nine 12-week intermittent treatment cycles and were due to begin their tenth.

### Endpoints

Clinical efficacy endpoints included assessment of endurance and muscle function (six-minute walk distance [6MWD] and other timed function tests), muscle strength (myometry and spirometry) and parent questionnaire data. Molecular efficacy endpoints included exon 51 skipping at mRNA level and dystrophin expression at protein level. Safety and tolerability endpoints included AEs, SAEs, local tolerability, laboratory parameters, vital signs, electrocardiograms, echocardiography, physical examination, and serum antidystrophin antibodies. AEs were captured as verbatim terms and coded using MedDRA System Organ Class and Preferred Term.

Pharmacokinetic evaluations included the maximum plasma concentration (C_max_), time to achieve C_max_ (T_max_), and the area under the plasma concentration-time curve from 0 to 24 hours (AUC_0–24h_). The AUC was calculated using non-compartmental analysis.

### Assessments

Efficacy, safety, and pharmacokinetic assessments were conducted at regular intervals throughout the 188-week extension study. The last efficacy assessments were conducted at week 177.

The 6MWD [7] was recorded at baseline, every 12 weeks until week 72 and then at weeks 80, 93, 105, 117, 129, 141, 153, 165, and 177. Absolute/percent change from original and extension baseline were reported. In addition, an age- and height-based equation fitted to normative data by Geiger *et al*. [23] was applied to the 6MWD data and a percent-predicted value of normal for each subject was calculated [24].

Other timed function tests (including 10 m walk/run, rise from floor and four-stair climb tests), muscle strength and respiratory function were assessed at baseline, week 8, monthly until week 28 and at weeks 36, 48, 60, 72, 80, 93, 105, 117, 129, 141, 153, 165, and 177. Muscle strength was evaluated by handheld myometry using a microFET dynamometer (Biometrics BV, Almere, Netherlands), whereas respiratory function was assessed using a handheld Koko spirometer (PDS Instrumentation, Louisville, KY, USA) and a magnehelic manometer (Dwyer Instrument, Michigan City, IN, USA). Respiratory function testing included the following parameters: forced vital capacity (FVC) [25], forced expiratory volume in 1 second (FEV_1_), maximal inspiratory pressure (MIP) [26], maximal expiratory pressure (MEP) [26], peak flow (PF) and peak cough flow (PCF) [27].

The parents completed questionnaires 4 or 5 times at various time-points during the extension study, depending on the subject and the study site. These short questionnaires captured loss of skills/daily activities, improvement of daily activities and development of new skills.

The anti-dystrophin antibody assay was based on a method described previously [28]; the presence of antibodies in serum was assessed at weeks 12, 24, 48, 72, 96, 120, 144 and 168.

Biopsies were taken from the tibialis anterior muscle at week 24 for all subjects in the extension study and at either week 68 or 72 for volunteering subjects. The muscle biopsies were used for assessment of drisapersen tissue concentrations, exon 51 skipping and dystrophin protein levels [21,29,30]. To detect exon 51 skipping, total RNA was isolated from 10 to 15 mg of muscle tissue and analyzed by means of nested reverse transcriptase-polymerase chain reaction (RT-PCR) assay and sequencing [21,29]. For detection of dystrophin expression, immunofluorescence analysis and Western blot analysis of total protein extracts isolated from muscle tissue were performed [30].

Blood samples for pharmacokinetic assessments were collected pre-dose and 3 hours post-dose at monthly intervals up to week 24, and pre-dose only from weeks 76 to 184. At week 20, samples were collected pre-dose and at 0.5, 2, 3, 4, 6, 9, 12, and 24 hours post-dose. Assessment of drisapersen plasma levels was performed as described previously [22].

### Statistical Analysis

No formal inferential statistical testing was performed due to the small number of subjects and the absence of a control group.

Descriptive statistics were used to summarize the data. SAS^®^ was used to calculate means, medians and standard deviations.

## Results

### Subjects

**Fig 1:**
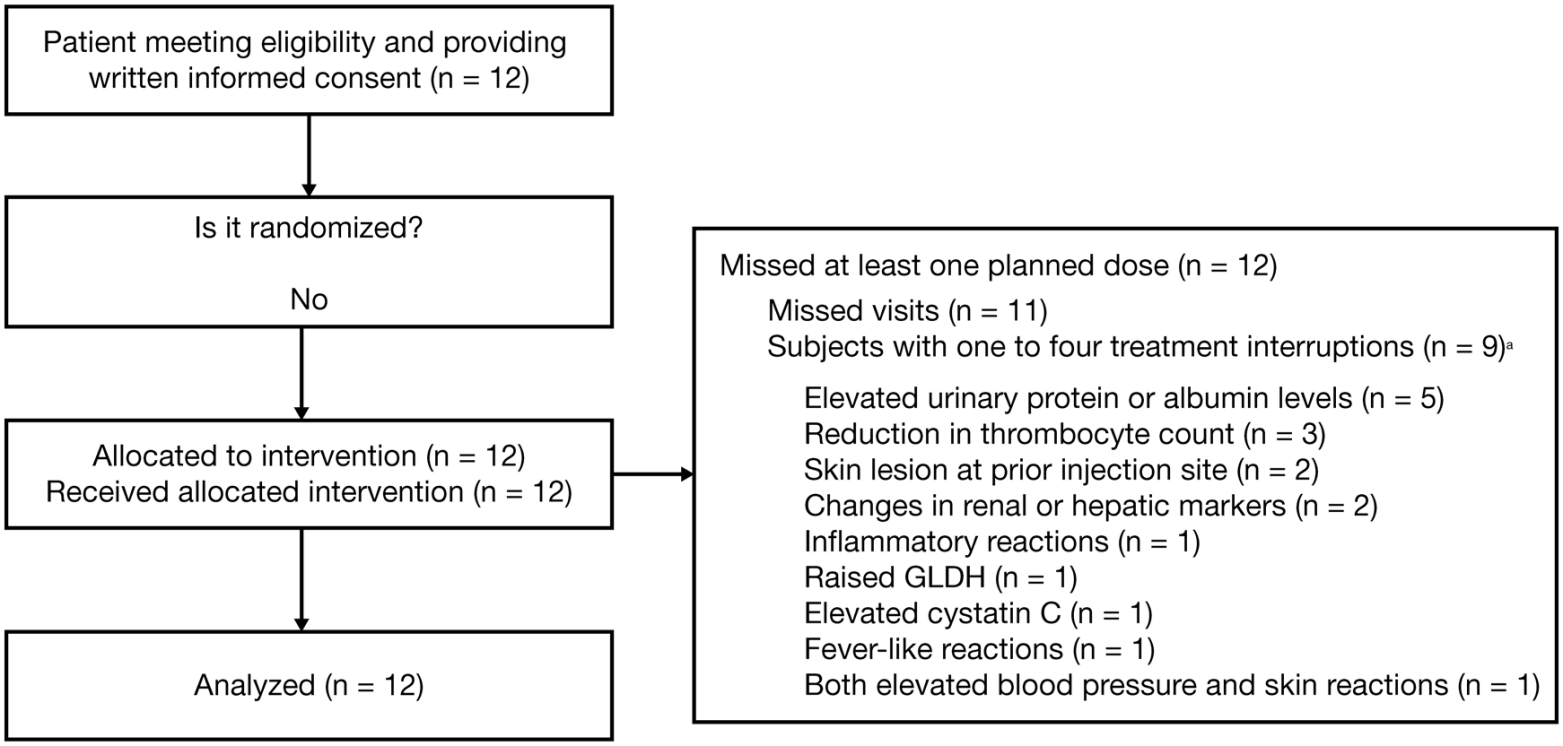
Patient flow diagram. ^a^Excluding planned washout and off-drug periods.

All 12 subjects who completed the dose-escalation study entered the 188-week extension study from July 2009 to April 2013. (Fig 1). The mean (median; range) age and mean (median; range) body mass index (BMI) of subjects at extension study baseline was 10.1 (10.1; 5.9 to 14.3) years and 19.3 (18.1; 15.4 to 30.7) kg/m^2^, respectively. Subjects received a mean dose of 5.93 mg/kg drisapersen per visit (range: 5.10 to 6.02 mg/kg), with the total dose ranging from 90 to 380 mg dependent on the subject’s body weight.

As reported previously [22], the study population was clinically heterogeneous at the start of the initial dose-escalation study, including a non-ambulant subject and ambulant subjects in different stages of disease. In the time interval between the dose-escalation and start of the extension study, an additional subject lost the ability to complete the 6MWD test. Demographic and clinical data at extension study baseline and week 177 are shown in Table 1.

**Table 1:** Demographic and clinical data for all subjects by disease status classification (extension study baseline), and use of ACE inhibitors and glucocorticosteroids during the extension phase.

		Extension study baseline					Week 177					Change from extension baseline to week 177 in 6MWD	
Subject	Exon deletion	Age (years)^a^	Height (cm)	Weight (kg)	BMI (kg/m^2^)	6MWD (meters)	Age (years)^a^	Height (cm)	Weight (kg)	BMI (kg/m^2^)	6MWD (meters)	Absolute change (meters)	Percent change (%)
**Subjects who walked ≥330 m at extension study baseline**													
**1**	52	10.9	124.0	27.8	18.1	374	14.3	129.0	29.5	17.7	521	147	39
**2**	45–50	8.0	119.7	25.5	17.8	406	11.4	131.5	32.5	18.8	505	99	24
**5**	48–50	10.3	122.0	25.8	17.3	647	13.7	126.3	32.0	20.1	625	-22	-3
**6**	48–50	7.5	114.0	24.6	18.9	429	10.9	119.5	25.6	17.9	466	37	9
**7**	45–50	9.2	117.9	24.4	17.6	340	12.6	123.0	26.9	17.8	503	163	48
**9**	45–50	5.9	97.5	14.6	15.4	350	9.3	109.0	20.3	17.1	441	91	26
**12**	45–50	9.9	114.5	27.8	21.2	500	13.3	125.5	36.8	23.4	302	-198	-40
**Subjects who walked <330 m at extension study baseline**													
**3**	48–50	14.3	141.0	36.8	18.5	0	17.7	146.0	44.7	21.0	0	NA	NA
**4**	52	11.8	141.0	61.0	30.7	75^b^	15.1	146.5	68.8	32.1	0^c^	NA	NA
**8**	45–50	12.0	125.5	28.3	18.0	287	15.4	128.5	34.2	20.7	231	-56	-20
**10**	48–50	9.6	132.2	30.9	17.7	263	12.9	138.6	42.3	22.0	0	-263	NA
**11**	48–50	11.4	135.9	38.1	20.6	243	14.8	142.0	41.0	20.3	0	-243	NA
**Concomitant medication use during the extension phase (safety population)**													
**ACE inhibitors**													
** Enalapril maleate, N (%)**			4 (33.3)										
**Glucocorticosteroids**^d^													
** Deflazacort, N (%)**			9 (75.0)										
** Prednisolone, N (%)**			4 (33.3)										

^a^Age was calculated from birth date.

^b^Subject 4 was unable to complete the 6MWD test at extension study baseline.

^c^Dose for Subject 4 was capped at 300 mg starting from week 54.

^d^Subject 6 was receiving prednisolone at study entry, but was changed to deflazacort during the study.

ACE: angiotensin-converting enzyme; BMI: body mass index; 6MWD: six-minute walk distance; NA: not available.

Disease progression in DMD is characterized by a non-linear pattern of evolution, with different slopes of decline at different stages of the disease. As 6MWD test results are influenced by baseline ambulatory status [8], subjects were retrospectively subdivided into those walking ≥330 m and <330 m at the extension study baseline. This subdivision reflected the clinical stage of the disease, with subjects walking ≥330 m considered to be in “plateau stage” or “stable” and those walking <330 m considered to be “in decline”; based on the judgement of the investigator, the subjects’ parents and physiotherapists according to medical notes and assessments.

All subjects were on stable daily corticosteroid treatment for at least 1 year before entry into the initial dose-escalation study, and remained on a stable continuous dose during the extension phase (Table 1). No changes in dosing regimen, other than for routine weight adjustment, were recorded for any subject, except for one subject who had a period of intermittent steroids implemented by his general practitioner because of delayed growth. Two subjects (3 and 8) were on stable angiotensin-converting enzyme (ACE) inhibitors at the baseline visit (Table 1). In two subjects (4 and 12), ACE inhibitors were initiated after week 48 (Table 1).

### Clinical Efficacy Endpoints

After 72 weeks continuous treatment with drisapersen, a washout for 8 weeks was scheduled before starting with an intermittent-dosing regimen (8 weeks of drisapersen followed by 4 weeks off-drug). Although data are limited, the introduction of an intermittent-dosing regimen following week 72 did not appear to adversely affect efficacy parameters.

#### 6MWD

Of the 12 subjects enrolled in the dose-escalation study, 11 were able to complete the 6MWD at baseline. At enrollment in the extension study, 10 subjects (mean age [SD]: 9.5 [1.9] years) were still able to perform the 6MWD at baseline, with (after an off-treatment period of 6–15 months) a median decrease in the 6MWD of 16 m (mean [SD]: -17.9 [58.9], range: -88 to 102 m) observed between the two study baselines (S1 Table). When the last efficacy assessment of the extension study was conducted (week 177) eight subjects were still ambulant, of whom five were able to walk further (range: 37 to 163 m) than at the extension baseline. The two subjects that lost ambulation during the extension study were older than 7 years of age and had a baseline 6MWD <330 m.

**Fig 2:**
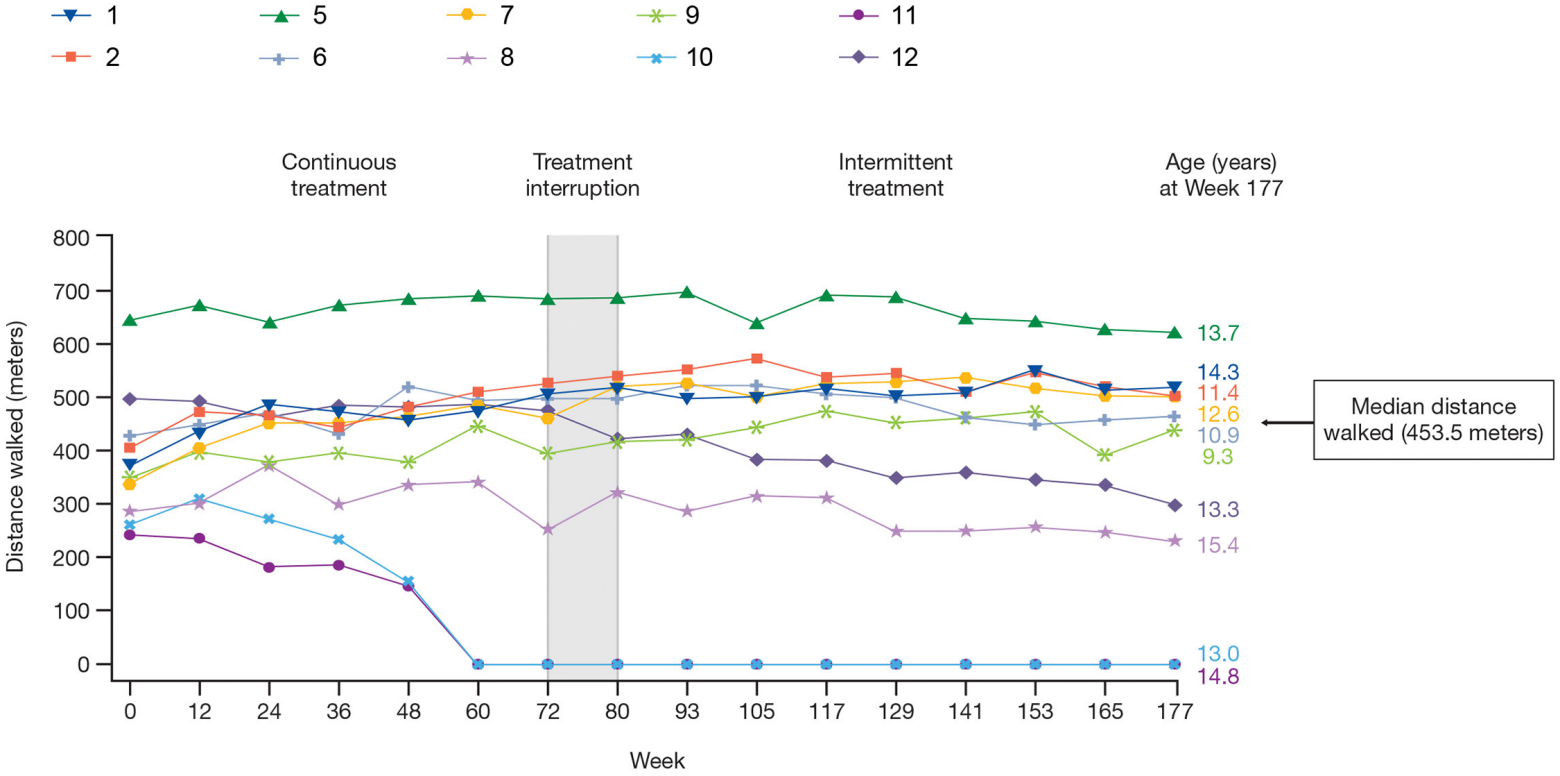
Change from extension study baseline in 6MWD, by visit over 177 weeks. Data shown are for all subjects who completed the test at the extension study baseline. One subject (Subject 3) was non-ambulant at study entry and did not participate in any 6MWD tests, while another subject (Subject 4) was unable to complete the 6MWD test at the extension study baseline. Data for both of these subjects are not shown here. Subjects 1, 2, 5, 6, 7, 9, and 12 walked ≥330 m at extension study baseline; subjects 8, 10, and 11 walked <330 m at extension study baseline. 6MWD: six-minute walk distance.

In Fig 2, the absolute changes in 6MWD are shown for the 10 subjects who completed the 6MWD at the extension baseline. The median (mean [SD]) 6MWD increased by 39 m (35.2 [28.7] m) over the first 12 weeks following the start of the extension study. The variability in 6MWD was high and increased with time, but in general the improvement was maintained at least up to week 129, when the median (mean [SD]) increase from the extension study baseline was 59 (-0.6 [165]) m. At week 177, the median (mean [SD]) change from the extension baseline in the 6MWD was 8 (-24.5 [161]) m. These summary statistics include two subjects who were unable to complete the test at later visits and whose results were reported as 0 m from that visit onwards (S1 Table). The median (mean [SD]) increase in 6MWD for the eight subjects still ambulant at week 177 was 64 (33 [121]) m.

Stratification of subjects by extension baseline ambulation status demonstrated that the two groups had notably different outcomes over the extension phase. Subjects walking ≥330 m (N = 7; mean age [SD]: 8.8 [1.8] years) generally had a more pronounced improvement in 6MWD with six subjects being able to walk further than at the extension baseline at the majority of subsequent visits (Table 1). At week 177, a median 91 m (range: -198 to 163 m) improvement in 6MWD was observed compared with the extension baseline. Five subjects improved (range: 37 to 163 m), of whom two walked more than 140 m further than at the extension study baseline (Table 1). Of those subjects walking <330 m (N = 5; mean age [SD]: 11.8 [1.7] years), two were non-ambulant at the extension baseline. The remaining three subjects deteriorated further, with two not able to complete the 6MWD after week 60 and one (subject 10; aged 15.4 years) still able to walk 231 m at week 177.

Percent-predicted values of normal for each subject are shown in Table 2. Six of the seven subjects walking ≥330 m at extension study baseline had percent-predicted values of greater than 70% of normal by week 93 in contrast to three at the extension baseline, despite five of these subjects being older than 7 years at the extension baseline. All subjects walking <330 m exhibited percent-predicted values below 55% at baseline, of whom only one retained percent-predicted values throughout the study.

**Table 2:** Percent-predicted normal 6MWD, as calculated using the Geiger equation [23,24].

Subject	Age at week 177 (years)	6MWD at extension study baseline (meters) (% normal)	6MWD at week 48 (meters) (% normal)	6MWD at week 141 (meters) (% normal)	6MWD at week 177 (meters) (% normal)
**1**	14.3	374 (59)	459 (71)	511 (78)	521 (79)
**2**	11.4	406 (69)	485 (80)	515 (81)	505 (78)
**5**	13.7	647 (104)	688 (108)	651 (100)	625 (96)
**6**	10.9	429 (75)	523 (89)	464 (75)	466 (74)
**7**	12.6	340 (56)	467 (76)	541 (85)	503 (78)
**8**	15.4	287 (45)	338 (52)	250 (38)	231 (35)
**9**	9.3	350 (69)	381 (71)	463 (80)	441 (74)
**10**	12.9	263 (42)	153 (24)	0	0
**11**	14.8	243 (37)	146 (22)	0	0
**12**	13.3	500 (82)	485 (78)	362 (56)	302 (46)

Subject 3 was non-ambulatory at study start and so could not take part in the 6MWD test at baseline.

Subject 4 was unable to complete the 6MWD test at extension study baseline.

Age was estimated using visit dates and the subject’s date of birth; results were rounded to the nearest 0.1 of a year. Height was taken from the nearest visit to the 6MWD assessment (visit schedules may differ by up to 3 weeks). The investigator could measure the height as standing (ambulant), lying down or by arm span assessment (non-ambulant), according to the stage of the disease.

6MWD: six-minute walk distance

#### Other timed function tests

Overall, for patients able to complete the rise from floor (N = 6), the stair climb (N = 7) and/or the 10 m walk/run tests (N = 8) through the extension study, no clear deterioration was observed and the test times were stable up to 177 weeks. The median (mean [SD]) change from the extension baseline to week 177 in the rise from floor test was 0.94 (1.05 [0.92]) seconds (N = 6); individual data are shown in S1 Fig. The median (mean [SD]) change from the extension baseline to week 177 in the 10 m walk/run (N = 8) and stair climb tests (N = 7) were 0.86 (1.61 [2.04]) seconds and 0.74 (4.23 [10.38]) seconds, respectively (S1 Fig). It should be noted that no imputation of missing data for patients unable to complete the test has been applied for summary statistics.

Stratification of subjects by extension baseline status demonstrated that subjects walking ≥330 m generally maintained their ability to complete all the tests throughout the extension study, whereas all subjects walking <330 m lost the ability to perform the rise from floor and stair climb test and only one subject walking <330 m was still able to perform the 10 m walk/run test.

#### Additional efficacy parameters

At week 165, the majority of parents considered their child’s walking ability (58%), endurance (83%), and general condition (75%) to be similar or improved compared with the beginning of the extension study. However, 67% felt their child’s ability to climb stairs had deteriorated. Questionnaires that captured changes in activities of daily living and anecdotal reports showed new or regained skills including running stairs, rope skipping, hopping, cycling, carrying heavy loads, and participating in sports with healthy peers in 5 subjects (1, 2, 5, 6 and 8). Furthermore, an improved exercise tolerance and endurance was reported in 6 subjects (1, 2, 5, 6, 7 and 8).

Muscle strength, measured as myometry and spirometry, appeared to decrease over 177 weeks. The high inter-individual variability makes it difficult to detect a clear overall trend, although there may have been trends in individual subjects. In general, there were no clinically relevant changes in myometry over 177 weeks (data not shown). A median percent-predicted decrease (mean [SD]) in FVC of 12.5 (-13.9 [13.46]) % was reported, whereas increases in absolute values of FVC (0.06; 0.06 [0.29] L), PF (42.0; 9.4 [73.06] L/min) and PCF (40.0; 22.5 [81.14] L/min) were observed (N = 12, S2 Table). It should be noted that one subject had an airway infection at 177 weeks, which may explain his bad performance at this visit compared to previous visits and which may have negatively affected the median and mean values [31]. When looking at the individual data, 8 of the 12 subjects showed less decline in FVC% as expected by natural history data (-4 to -5% per year). PCF and PF% even improved from baseline in 9 and 7 of the 12 subjects, respectively.

### Safety and Tolerability

All subjects reported one or more AEs. The most common AEs are shown by study period in S3 Table, whereas all treatment-related AEs are reported in Table 3. The main AEs occurring in all 12 subjects were injection-site reactions (erythema, hematoma, and induration), increased urinary α_1_-microglobulin, and proteinuria.

**Table 3:** Treatment-related adverse events during the 188-week extension phase.

System-organ class	Treatment-realted adverse event N (%)	Number (%) of subjects (N = 12)
**Blood and lymphatic system disorders**	Thrombocytopenia	5 (42)
	Leukopenia	2 (17)
**Gastrointestinal disorders**	Vomiting	3 (25)
	Diarrhea	2 (17)
	Nausea	2 (17)
	Upper abdominal pain	1 (8)
**General disorders and administration-site conditions**	Injection-site induration	12 (100)
	Injection-site erythema	12 (100)
	Injection-site hematoma	12 (100)
	Injection-site pain	10 (83)
	Injection-site discoloration	9 (75)
	Injection-site atrophy	6 (50)
	Injection-site pruritus	6 (50)
	Injection-site dryness	3 (25)
	Injection-site inflammation	3 (25)
	Injection-site irritation	3 (25)
	Injection-site reaction^a^	3 (25)
	Injection-site ulcer	3 (25)
	Pyrexia	2 (17)
	Influenza-like illness	2 (17)
	Injection-site rash	1 (8)
**Infections and infestations**	Gastroenteritis	2 (17)
	Injection-site infection	2 (17)
	Influenza	1 (8)
**Investigations**	α_1_-microglobulin urine increase	12 (100)
	Cystatin C increase	10 (83)
	Glutamate dehydrogenase increase	9 (75)
	Elevated MCP-1	5 (42)
	Urinary sediment abnormal	5 (42)
	Complement factor C3 decrease	3 (25)
	γ-glutamyltransferase increase	3 (25)
	Haptoglobin increase	3 (25)
	C-reactive protein increase	2 (17)
	White blood cells urine-positive	2 (17)
	Haematocrit decreased	2 (17)
	Haemoglobin decreased	2 (17)
	Red blood cell count decreased	2 (17)
	Troponin I increase	2 (17)
	White blood cell count decreased	2 (17)
**Musculoskeletal and connective tissue disorders**	Pain in extremity	1 (8)
	Back pain	1 (8)
	Myalgia	1 (8)
	Muscle spasms	1 (8)
**Nervous system disorders**	Headache	4 (33)
**Renal and urinary disorders**	Proteinuria	12 (100)
	Albuminuria	11 (92)
**Skin and subcutaneous tissue disorders**	Dry skin	2 (17)
	Alopecia	2 (17)
	Erythema	1 (8)

^a^Reported as an “injection-site reaction” with no specific description.

MCP1: monocyte chemotactic protein-1.

Local injection-site reactions in the abdominal sc tissue were the most prominent AEs of clinical note. The abdomen was the exclusive site of drug administration up to week 50 to 72. Given there was potential for injection-site reactions with repeated administration, a rotation schedule for injection was recommended. The majority of injection-site reactions were considered mild to moderate, but were slow to reverse or persisted. Reactions at the injection site included: erythema, hematoma and induration, pain, discoloration/pigmentation, nodules and sclerosis, with the appearance of sclerodermic scars or morphea-like skin changes, which occurred after chronic administration. In two cases ulceration developed following scratching of sclerotic skin, but healed with wound care.

**Fig 3:**
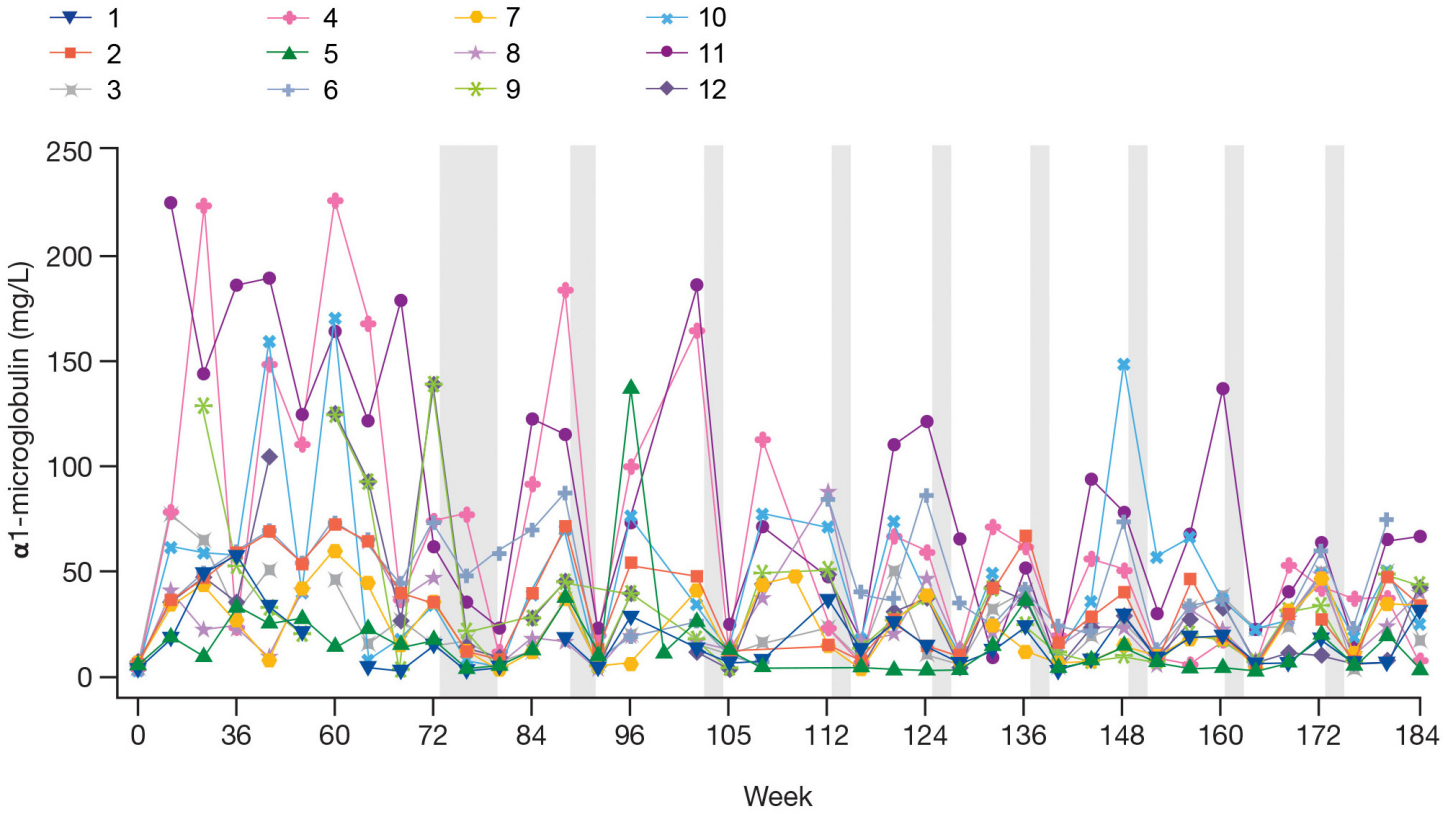
α_1_-Microglobulin levels over 177 weeks. Subjects 1, 2, 5, 6, 7, 9, and 12 walked ≥330 m at extension study baseline; subjects 3, 4, 8 10, and 11 walked <330 m at extension study baseline. Grey shading is representative of off-treatment periods.

Increases in urinary α_1_-microglobulin levels and proteinuria (in spot-urine samples; defined as ≥0.2 g/L) were each reported for all subjects and were considered to be mild in severity and treatment-related. Fig 3 shows α_1_-microglobulin levels in all 12 subjects over a 177-week period, indicating decreasing values during the drug-free periods, with the majority returning towards normal, followed by an increase upon resumption of treatment. All 12 subjects had at least one 24-hour urine collection by week 175 as a result of a predefined criterion of spot protein levels measured at ≥0.2 g/L in two consecutive samples. For six subjects, 24-hour urine protein levels were ≥0.15 g/day; however, none exceeded 0.3 g/day on a sustained basis. Increased urinary levels of α_1_-microglobulin and proteinuria were widespread during the continuous treatment phase (N = 12 [100%] and N = 11 [92%]), but were reported for fewer subjects during the interruption/intermittent treatment phase (N = 7 [58%] and N = 6 [50%], respectively).

There was evidence of changes in some hepatic parameters in response to the interruption/intermittent treatment phase: e.g. two subjects had elevated alanine aminotransferase, gamma-glutamyl transferase and glutamate dehydrogenase during the on-drug period and decreases during the off-drug periods. There was no evidence of overt drug-induced hepatotoxicity in any of the subjects. Furthermore, no anti-dystrophin antibodies were detected (data up to week 96).

Cardiac function measured using echocardiography was variable but showed stability over the 188-week extension study with some tendency for improvement in two subjects who walked <330 m at the start of the study (S4 Table). Thrombocyte counts, which slightly decreased during the continuous treatment phase, stabilized following introduction of the intermittent-dosing regimen.

Overall, most treatment-related AEs were classified as mild. Of the moderate AEs, most were related to injection-site reactions and one subject had moderate thrombocytopenia (thrombocyte count <150×10^9^/L). None of eight SAEs occurring in five subjects (tympanic membrane perforation, femur fracture, tibia fracture, scoliosis surgery and tendon contracture release, febrile convulsion, scrotal pain, and post-operative care for dental extraction) were considered treatment-related.

None of the subjects permanently discontinued the study. However, nine subjects had up to four treatment interruptions (excluding planned washout and off-drug periods) due to AEs and/or laboratory abnormalities. In general, biochemical parameters moved towards baseline levels off-treatment and inflammatory/fever-like reactions (e.g. low-grade fever, aches, flu-like symptoms) following drug administration in two subjects were manageable with nonsteroidal anti-inflammatory drugs (NSAIDs) and/or antihistamines. Of three subjects with treatment interruptions of maximally 7 weeks related to mild thrombocytopenia, the reductions in thrombocyte count were reversible without further intervention. None of the treatment interruptions resulted in hospitalizations.

### Pharmacokinetic Profile

After sc injection of 6 mg/kg drisapersen, the drug was quickly absorbed, with peak levels (T_max_) generally reached at 2–3 hours. Thereafter, drisapersen was rapidly distributed with a decline in plasma levels to approximately 18% of the C_max_ at 24 hours, and 0.6% at the end of the dose interval (7-day trough levels). Peak plasma levels and AUC_0–24h_ showed no clear increase after repeated dosing, but mean trough plasma levels increased with increasing numbers of injections up to at least the first 20 weeks of treatment, indicating continued tissue exposure at once-weekly dosing intervals.

**Fig 4:**
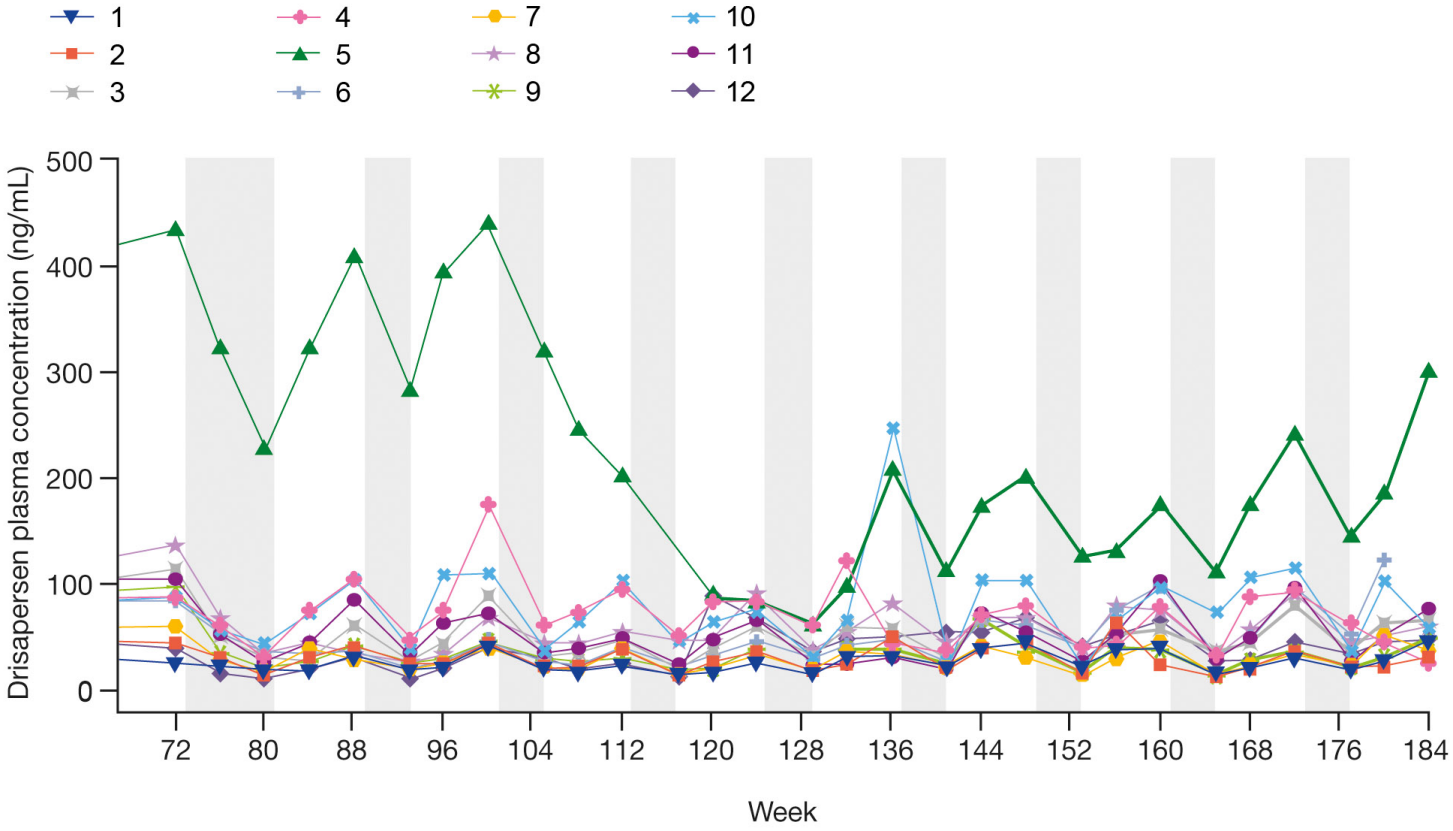
Individual drisapersen trough concentrations from weeks 72 to 184. Subject 5 had an additional treatment break from weeks 101 to 128 inclusive. Subjects 1, 2, 5, 6, 7, 9, and 12 walked ≥330 m at extension study baseline; subjects 3, 4, 8 10, and 11 walked <330 m at extension study baseline. Grey shading is representative of off-treatment periods.

Plasma levels of drisapersen decreased in all subjects during the washout and off-treatment periods, indicating clearance of the drug (Fig 4). Trough concentrations increased during the dosing periods, suggesting a likely decrease in tissue exposure during off-periods of the intermittent-dosing regimen. One subject had notably higher trough plasma levels; the reason for this apparent outlier is unknown. This subject had an extended treatment interruption (week 101–128) due to a leg ulcer, during which trough plasma concentrations declined and were within the range of the other subjects at treatment reinitiation. However, in the following weeks, they increased again to levels higher than those in other subjects.

### Biopsy Analysis

Drisapersen concentrations were measured in the tibialis anterior muscle biopsies of all subjects at week 24 (N = 12) and at week 68 or 72 (N = 8). Due to biopsy handling and/or shipping issues, the quality of the muscle biopsies taken at week 24 were seriously compromised. The mean (SD) drisapersen levels in the tibialis anterior muscle were higher at week 68/72 compared with week 24: 20.3 (9.6) and 14.4 (6.7) μg/g of tissue, respectively. Exon 51 skipping and dystrophin expression were detected in the biopsies at both time-points, yet no conclusion on an increase from baseline could be drawn because no material from pre-treatment biopsies was available.

## Discussion

Antisense-induced exon skipping is considered a promising new strategy for the treatment of DMD [32]. To our knowledge, this 188-week open-label extension of the dose-escalation study [22] of drisapersen (6 mg/kg sc) in 12 boys with DMD is the longest reported follow-up of any exon skipping therapy and provides the longest-duration published dataset on any oligonucleotide administered sc. We demonstrated that long-term treatment with drisapersen appears to be well tolerated in subjects over 188 weeks, with encouraging effects on efficacy parameters at 177 weeks (last efficacy assessment).

Of the 12 subjects enrolled in the dose-escalation study [22], 10 were able to complete the 6MWD at the extension baseline (time interval between two baselines: 6–15 months). Drisapersen treatment resulted in a median (mean [SD]) 8 (-24.5 [161]) m improvement in 6MWD at week 177. However, two subjects were unable to perform the 6MWD at week 60 whose results were reported as 0 m from that visit onwards. When only the eight ambulant subjects at week 177 are taken into account, a median (mean [SD]) increase of 64 (33 [121]) m in 6MWD was observed.

**Fig 5:**
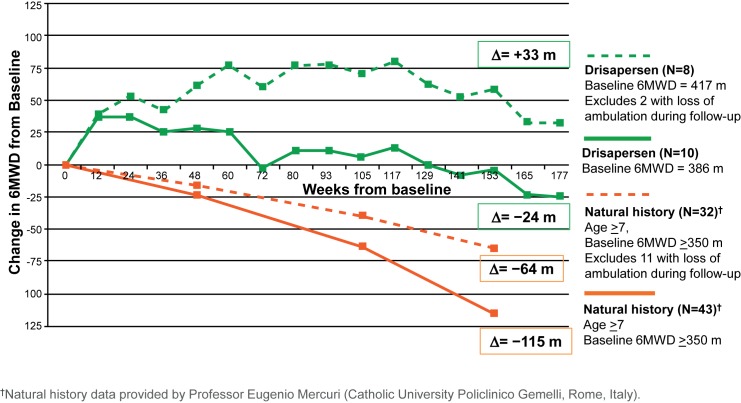
Comparison of change from baseline in 6MWD from drisapersen-treated subjects with natural history data. The continuous line includes all patients from the drisapersen cohort or natural history cohort for which the 6MWD was available, whereas the dashed line shows the same cohorts but excludes patients that lost ambulation during follow-up. ^†^Natural history data provided by Professor Eugenio Mercuri (Catholic University Policlinico Gemelli, Rome, Italy) 6MWD: six-minute walk distance.

Although caution must be exercised when comparing studies, the 6MWD results represent a clear deviation from natural history data (Fig 5), demonstrating an average decrease of 40–60 m in mean 6MWD over the course of 1 year [4,7–10,33,34]. This decline in 6MWD is not consistent over a child’s life. Several large natural history study cohorts report that generally DMD patients who are younger than 7 years improve or remain stable in 6MWD, whereas those older than 7 years generally show progressive deterioration [4,7–10,33]. In a recent long-term DMD natural history study, subjects younger than 7 years had some improvement in the first 24 months (mean [SD] 6MWD = 34.1 [88.8] m) but this was not maintained at 36 months of follow-up (mean [SD] 6MWD = -2.9 [117.7] m). In contrast, boys older than 7 years showed progressive deterioration, with a mean (SD) decrease in 6MWD of 146 (146.2) m at 36 months [8]. All, except one, of the subjects in our study were older than 7 years at the start of the extension study (mean age [range] at the extension baseline 10.1 [5.9, 14.3] years) and would be expected to show a declining 6MWD during the study period based on these longitudinal observations. However, five of the 10 ambulant subjects at the extension baseline improved, of whom two (aged 14.3 and 12.6 years) were able to walk more than 140 m further at week 177. Furthermore, parents and teachers reported increased stamina and participation in activities with peers; some boys acquired skills or regained skills that had been lost, at an age at which this would not be expected in DMD.

Our study included a clinically heterogeneous group of subjects, at different stages of disease progression. Recently published data indicates that patients who have a 6MWD of >330 m have reduced risk of losing ambulation within a one to three year period [8,9,33]. Classification of subjects by their ambulatory disease status at extension baseline demonstrated notable differences in the 6MWD over 177 weeks. In the seven subjects walking ≥330 m at extension study baseline, a median improvement of 91 (range: -198 to 163) m in 6MWD was observed over 177 weeks, with six able to walk further than baseline at the majority of subsequent visits. In addition, they showed a stable maintenance of function in the other timed function tests. Of the five subjects walking <330 m at extension study baseline, three were ambulant at the extension baseline. These three subjects deteriorated further, with two losing ambulation after 60 weeks. They also gradually lost the ability to complete other timed function tests, with the ability to rise from floor firstly deteriorating. Subjects walking <330 m were on average older, taller and heavier than subjects walking ≥330 m. In addition, they exhibited percent-predicted values below 55%, also reflecting a more advanced stage of disease [8,9,33]. This may indicate that a critical amount of residual muscle fiber is required to prevent further loss of ambulation with this therapeutic strategy, a theory which is supported by results in mice receiving exon skipping therapy (morpholino oligomers) [35]. In these subjects with advanced-stage disease, long-term assessment of upper limb, cardiac, and respiratory function is needed to evaluate any potential disease-modifying effect of treatment.

We compared the performance of subjects with DMD with healthy peers, by calculating a percent-predicted value to account for normal growth and development [23]. However, it is important to keep in mind that steroid treatment for DMD causes growth retardation compared with healthy peers. A previous study demonstrated that subjects aged 4–7 years maintained a stable 6MWD at approximately 80% of that of their typically developing peers, whereas in subjects older than 7 years, there was a variable decline in percent-predicted 6MWD [24]. All subjects walking ≥330 m at extension study baseline had percent-predicted values of >70% by week 93, of which six showed an increase in percent-predicted values despite two being close to the threshold value (55%). All subjects walking <330 m at extension study baseline exhibited percent-predicted values below 55% at baseline, of whom one retained stable percent-predicted 6MWD values despite his age and baseline 6MWD.

Although the 6MWD test has been adapted to evaluate muscle function and endurance in neuromuscular diseases and has been chosen as the primary outcome measure in several international multicenter clinical trials in DMD [34,36], it has limitations in a heterogeneous patient population that includes individuals with reduced baseline ambulatory capacity. The major disadvantage of the 6MWD test is that boys who become non-ambulant as part of the natural progression of DMD are unable to perform the test at later time points. Therefore, including additional parameters such as muscle strength, respiratory function and performance of upper limb (PUL) in ambulant boys would not only provide additional information but also allows following boys who may lose ambulation during the trial [37–39]. To address the issue of the impact of motivation and cooperation on the results of the 6MWD, subjects were tested in a standardized way by the same evaluator, using standardized encouragements. A learning effect can be excluded, considering the fact that these subjects were used to perform the 6MWD before the start of the extension study.

Cardiac function remained remarkably stable over the time frame of 188 weeks. Muscle strength generally declined over time in both subjects walking ≥330 m or <330 m, though the results on a by-subject basis were highly variable. Although the respiratory function assessment results were also highly variable, an encouraging trend was observed in comparison with published natural history data. Based on these data, reporting a linear decline in percent-predicted FVC and percent-predicted PF of approximately 5% per year in DMD subjects aged 5 to 24 years, a decline of 17% over 3.4 years would be anticipated [31,40,41]. However, a mean decline of 12.5% predicted FVC and of 0.8% predicted PF was observed in the current study. In addition, the stabilization of the percent-predicted PF and the improvements in PCF are particularly encouraging as these measures assess expiratory strength and inspiratory effort [42]. Although caution must be exercised when comparing percent-predicted respiratory results, as no comparison can be made if the equations used are different and if the patients that are compared did not all receive corticosteroid treatment (which delays the decline in respiratory function).

Long-term treatment with drisapersen was well tolerated in subjects over 188 weeks. The majority of AEs were considered to be mild and there were no treatment-related SAEs. Furthermore, none of the subjects had any detectable antibodies to dystrophin during this extension study, mitigating concerns about the novel production of dystrophin protein triggering an immune response.

The most common AEs were injection-site reactions, which were reported as mild AEs in the majority of patients as they were not associated with discomfort and did not interfere with activities of daily living. However, a number of individual injection-site reactions were of concern owing to the time taken for the subject’s skin to recover and/or the persistent nature of the reaction, such as sclerotic/fibrotic changes in the skin (that do not recover) or skin fragility with the risk of poor healing of wounds and ulcerations. Rotating the dosing in alternative locations may help to delay the development and progression of injection-site reactions. Alternatively, an intravenous administration formulation for drisapersen is currently under investigation. Although there was evidence of mild proteinuria and raised urinary α_1_-microglobulin levels, renal effects did not appear to be progressive, with values moving towards normal ranges during drug-free periods, which may indicate mild reversible interference with protein reabsorption in the proximal tubule. There were no signs of proximal tubule dysfunction (Fanconi’s syndrome).

No subjects died or permanently withdrew during the long-term extension study. Although nine subjects had treatment interruptions, none met the predefined stopping criteria. All biochemical parameters moved towards baseline levels off-treatment and systemic inflammatory reactions (e.g. flu-like symptoms) appeared to be manageable with NSAIDs and/or antihistamines.

Introduction of an intermittent-dosing regimen (8 weeks drisapersen followed by 4 weeks off-treatment) following week 80 did not appear to adversely affect efficacy. However, there is evidence that some safety parameters stabilized, either for the group as a whole or for individuals in whom parameters were trending up or down. In particular, the increases in α_1_-microglobulin and proteinuria reported in all subjects as mild AEs appeared to decrease during the drug-free periods.

RNA and protein analysis of more than 300 muscle biopsies, in three parallel running placebo-controlled drisapersen studies, revealed the occurrence of low levels of spontaneous exon 51 skipping and variably low levels of resulting trace dystrophin expression in the majority of patients at baseline and in the absence of treatment. This “natural rescue mechanism” necessitates a careful comparison of pre- and post-treatment biopsies collected from the same muscle group for each patient, to assess a molecular treatment effect. Inherent to the original study design, no material from the pre-treatment biopsies was available to allow such assessment. However, the fact that plasma trough levels increased with repeated administration suggests gradually increasing drisapersen levels in muscle tissue. Average tissue drisapersen concentrations measured in the biopsies were higher than 10 μg/g of muscle tissue at both week 24 and 68/72. Tissue levels of this magnitude have been shown to lead to an increased dystrophin signal intensity [36].

While our study provides important long-term data on drisapersen, its limitations must be acknowledged. The study was not designed to prove efficacy as it was a small, open-label study lacking a placebo control group. Therefore, functional outcomes need to be interpreted with caution. Despite the limitations and the heterogeneous study sample, evidence of an improvement in median 6MWD values was found compared to the expected decline reported by natural history studies. Additional analysis is ongoing comparing these 12 subjects to a drisapersen-naïve age (±6 months) and 6MWD (±30 m) matched population from a natural history cohort. All drisapersen-treated subjects had an exon 51 skippable mutation, whereas the NH subjects were a generalized DMD population with confirmation of individual mutations (Goemans N, submitted).

In conclusion, long-term treatment (3.4 years) with drisapersen appears to be well tolerated in subjects with DMD, with encouraging impact on efficacy parameters. In contrast to the expected decline in the 6MWD over time, there was evidence of improvement, in particular in the subjects who remained ambulant during the study or walked ≥330 m at the extension study baseline. Subjects walking ≥330 m at the extension study baseline largely retained performance on timed function tests and respiratory assessments in the face of a rapidly progressive disease. Even though these are promising results, drisapersen has not been approved for treatment of DMD patients by the FDA.

## Supporting Information

S1 Fig Changes from extension study baseline by visit over 177 weeks. A. Rise from floor time (N = 9). B. 10 meter walk/run (N = 11). C. Stair climb time (N = 11). Subjects 1, 2, 5, 6, 7, 9, and 12 walked ≥330 m at extension study baseline; subjects 4, 8, 10, and 11 walked <330 m at extension study baseline. Subject 8 was non-ambulant at study entry and did not participate in any of the timed tests. Subjects 1, 3 and 4 were not able to perform the rise from floor test, whereas subject 3 was unable to perform the 10 meter walk/run and stair climb test. It should be noted that no imputation of missing data for patients unable to complete the test has been applied.
(TIF)

S1 Methods Subjects. Briefly, subjects were eligible if they had an estimated life expectancy of 6 months or more, no serious pre-existing medical conditions, and no dependency on assisted ventilation, and had not participated in any other study with an investigational product in the past 6 months. Concurrent glucocorticosteroid treatment was permitted if stable for at least 2 months prior to enrollment, and was to be kept constant during the study if possible.
(DOCX)

S1 Protocol The protocol for this trial and the supporting is available as supporting information. (PDF)

S1 Results Subjects. Overall compliance was calculated to be 92.1%, and the mean (range) dose was 5.93 (5.10–6.02) mg/kg.
(DOCX)

S1 Table Actual distance walked and change from baseline in the 6MWD test for all subjects and by disease status at extension study baseline (intent-to-treat population): subjects able to complete the 6MWD test at extension study baseline. Missing values replaced by zero for subjects who became unable to complete the test at later visits. ^a^Two subjects were not able to complete the 6MWD test at extension study baseline; two additional subjects did not complete the 6MWD test at week 60. 6MWD: six-minute walk distance; SD: standard deviation.
(DOCX)

S2 Table Summary of spirometry parameters over 177 weeks: absolute values and changes from extension study baseline (intent-to-treat population). FEV_1_: forced expiratory volume in 1 second; FVC: forced vital capacity; MEP: maximal expiratory pressure; MIP: maximal inspiratory pressure; PCF: peak cough flow; PF: peak flow; SD: standard deviation.
(DOCX)

S3 Table AEs^a^ that occurred in at least two subjects during the 188-week extension phase. ^a^Includes all AEs (classified as both treatment related and unrelated) ocurring in at least two subjects in either the continuous or the intermittent treatment phases. ^b^Reported as an “injection-site reaction” with no specific description. ^c^Only two of these three subjects were described by the investigator as having ulcers; the third subject was reported as having wounds at the injection site, which the investigator has confirmed were not ulcers. AE: adverse events; MCP1: monocyte chemotactic protein-1.
(DOCX)

S4 Table Echocardiography during continued treatment (safety population). Only data for scheduled visits at weeks 24, 48, 72, 96, 120, 144 and 168 are displayed in this table.
(DOCX)

S1 TREND Checklist The TREND checklist is available as supporting information. (PDF)
