# Supplementary figures and images for: Long-Term Efficacy, Safety, and Pharmacokinetics of Drisapersen in Duchenne Muscular Dystrophy: Results from an Open-Label Extension Study
Source: PLoS One. 2016 Sep 2;11(9):e0161955. doi: 10.1371/journal.pone.0161955 (PMC5010191; doi:10.1371/journal.pone.0161955)

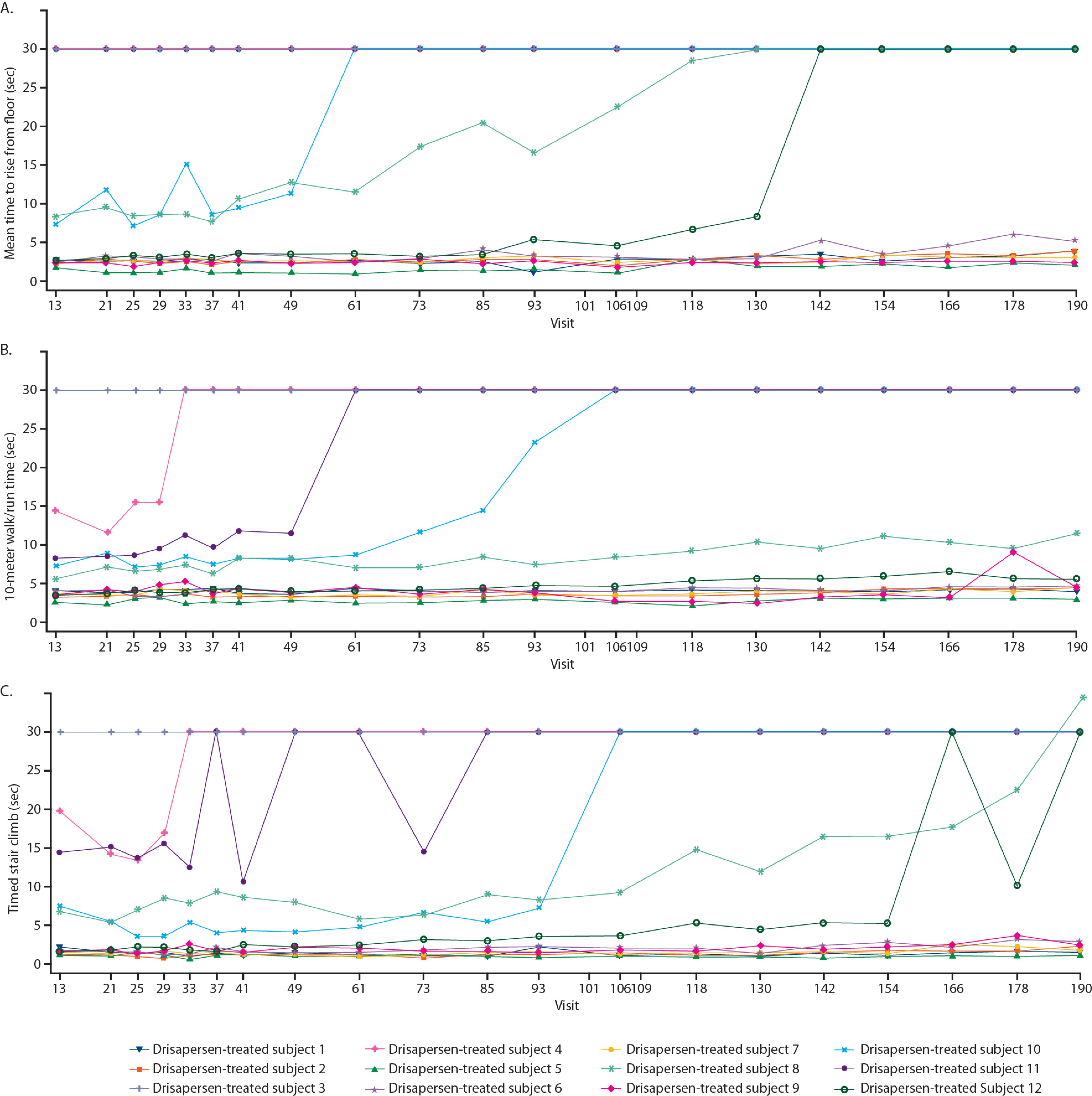

Supplement: S1 Fig — A. Rise from floor time (N = 9). B. 10 meter walk/run (N = 11). C. Stair climb time (N = 11). Subjects 1, 2, 5, 6, 7, 9, and 12 walked ≥330 m at extension study baseline; subjects 4, 8, 10, and 11 walked <330 m at extension study baseline. Subject 8 was non-ambulant at study entry and did not participate in any of the timed tests. Subjects 1, 3 and 4 were not able to perform the rise from floor test, whereas subject 3 was unable to perform the 10 meter walk/run and stair climb test. It should be noted that no imputation of missing data for patients unable to complete the test has been applied. (TIF) [file pone.0161955.s001.tif]
