# Supplementary material for: Long-Term Efficacy, Safety, and Pharmacokinetics of Drisapersen in Duchenne Muscular Dystrophy: Results from an Open-Label Extension Study
Source: PLoS One. 2016 Sep 2;11(9):e0161955. doi: 10.1371/journal.pone.0161955 (PMC5010191; doi:10.1371/journal.pone.0161955)
Supplement: S1 Methods — Briefly, subjects were eligible if they had an estimated life expectancy of 6 months or more, no serious pre-existing medical conditions, and no dependency on assisted ventilation, and had not participated in any other study with an investigational product in the past 6 months. Concurrent glucocorticosteroid treatment was permitted if stable for at least 2 months prior to enrollment, and was to be kept constant during the study if possible. (DOCX) [file pone.0161955.s002.docx]

## S1 Methods

### Subjects

Briefly, subjects were eligible if they had an estimated life expectancy of 6 months or more, no serious pre-existing medical conditions, and no dependency on assisted ventilation, and had not participated in any other study with an investigational product in the past 6 months. Concurrent glucocorticosteroid treatment was permitted if stable for at least 2 months prior to enrollment, and was to be kept constant during the study if possible.
