# Supplementary material for: Long-Term Efficacy, Safety, and Pharmacokinetics of Drisapersen in Duchenne Muscular Dystrophy: Results from an Open-Label Extension Study
Source: PLoS One. 2016 Sep 2;11(9):e0161955. doi: 10.1371/journal.pone.0161955 (PMC5010191; doi:10.1371/journal.pone.0161955)
Supplement: S1 Protocol — (PDF) [file pone.0161955.s003.pdf]

Clinical Study Protocol

A phase I/II, open label, escalating dose, pilot study to assess the effect, safety, tolerability and pharmacokinetics of multiple subcutaneous doses of PRO051(GSK2402968)in patients with Duchenne muscular dystrophy

CONFIDENTIAL

**EudraCT number**	2007-004819-54
**Protocol number**	PRO051-02
**Revised to incorporate Amendment 11**	17 May 2012
**Amendment 10**	10 August 2011
**Amendment 9**	14 April 2011 (to be implemented on 21 July 2011)
**Amendment 8**	9 March 2011
**Amendment 7**	15 November 2010
**Amendment 6**	16 July 2010
**Amendment 5**	2 September 2009
**Amendment 4**	11 June 2009
**Amendment 3**	27 April 2009
**Amendment 2**	5 May 2008
**Amendment 1**	21 March 2008
**Original Protocol**	7 December 2007

Sponsor

GlaxoSmithKline
Iron Bridge Road
Stockley Park West, Uxbridge, Middlesex,
UB11 1BU, UK
Telephone: [redacted]

Copyright 2011 the GlaxoSmithKline group of companies. All rights reserved. Unauthorised copying or use of this information is prohibited.

Protocol synopsis

**Protocol number**	PRO051-02	
**EudraCT number.**	2007-004819-54	
**Name of sponsor**	Prosensa Therapeutics B.V.	
**Name of finished product**	PRO051	
**Name of active ingredient**	2'-O-methyl-phosphorothioate antisense oligoribonucleotide named h51AON23	
**Title of study**	A phase I/II, open label, escalating dose, pilot study to assess the effect, safety, tolerability and pharmacokinetics of multiple subcutaneous doses of PRO051 in patients with Duchenne muscular dystrophy	
**Investigators**	N. Goemans, M.D., UZ Leuven (campus Gasthuisberg), Leuven, Belgium<br>M. Tulinius, M.D., The Queen Silvia Children's Hospital, Göteborg, Sweden	
**Study centers**	UZ Leuven (campus Gasthuisberg), Leuven, Belgium<br>The Queen Silvia Children's Hospital, Göteborg, Sweden	
**Publications (reference)**	N.A.	
**Planned study period**		**Phase of Development**
**(date of first enrolment)**	March 2008	Phase I/II
**(date of last completed)**	June 2009<br>(study period)	
**(date of last completed)**	2013<br>(treatment beyond study period)	
**Diagnosis and main criteria for inclusion**	1. Age at least 5 and not older than 16 years on the day of first drug administration<br>2. Duchenne muscular dystrophy resulting from a mutation correctable by treatment with PRO051<br>3. Not ventilator dependent<br>4. Life expectancy at least 6 months<br>5. No previous treatment with investigational medicinal treatment within 6 months prior to the study	
**Study objective(s)**	To preliminarily assess the effect of PRO051 at different dose levels in patients with Duchenne muscular dystrophy<br>To assess the safety and tolerability of PRO051 at different dose levels in patients with Duchenne muscular dystrophy<br>To determine the pharmacokinetics of PRO051 at different dose levels after subcutaneous administration in patients with Duchenne muscular dystrophy.	
**Target disease**	Duchenne muscular dystrophy	
**Design and methodology**	A phase I/II, open-label, escalating dose, multiple dose, pilot study.<br>Patients will receive one subcutaneous treatment of PRO051 per week for a period of five weeks (i.e. five	

**Protocol number**	PRO051-02
**EudraCT number.**	2007-004819-54
	injections in total). Depending on a possible maximum tolerated dose, twelve to eighteen patients will be investigated in four groups. Each consecutive group will consist of three patients who will be treated with maximally 0.5 mg/kg, 2 mg/kg, 6 mg/kg and 10 mg/kg, respectively. Effect and safety assessments will be done at regular intervals. Prior to each dose escalation, the safety data of the first three weekly administrations of PRO051 will be reviewed. If possible dose limiting toxicity occurs in one patient of the group of three, the group will be expanded to six patients.<br>If similar dose limiting toxicity occurs in two or more patients, the inclusion of new patients will be discontinued at that dose level. Furthermore, the inclusion of new subjects into the study may be stopped, or study may continue with a lower intermediate dose level, or study may continue by expanding the cohort of the previous dose level to six subjects. The dose level of the group which has a dose level one step below the group with similar dose limiting toxicity in two or more patients, will be considered the maximum tolerated dose (MTD).
**Subject sample size**	12-18 patients.
**Test product**	
**- Batch number**	07P51-001 (study period)
**- Batch number**	09P51-001 (treatment beyond study period up to Visit 113)
**- Dose and rationale**	PRO051 administered subcutaneously with one week between administrations. Five administrations in total. Maximum dose levels to be administered:<br>0.5 mg/kg<br>2 mg/kg<br>6 mg/kg<br>10 mg/kg
**- Mode of administration**	Subcutaneous injections
**Reference therapy**	Not applicable
**- Batch number**	Not applicable
**- Dose and rationale**	Not applicable
**- Mode of administration**	Not applicable
**Duration of treatment and duration of study**	18 weeks, consisting of 5 weeks of treatment plus 13 weeks follow up.<br>Provided patient and/or his parents, the investigator and the sponsor all agree that drug administration appears to improve the clinical status of the patient without direct safety concerns, patients will be offered the option to continue treatment with weekly (or alternative) dose administrations after the 13-week follow up period, accompanied by safety, effect and pharmacokinetic assessments at regular intervals. The dose level during the treatment beyond study

**Protocol number**	PRO051-02
**EudraCT number.**	2007-004819-54
	period will be decided for each individual subject on a case-by-case basis. All safety, effect and pharmacokinetic data that are available at the time of the decision will be taken into account. Dose level may be adjusted in case new safety, effect and/or pharmacokinetic data become available.
**Concomitant medication**	Anticoagulants, antithrombotics and antiplatelet agents are not allowed during the study.<br>Other concomitant medication including glucocorticosteroids is allowed; however, if possible medication usage should not change during the study.<br>Glucocorticosteroid use should be stable for at least 2 months prior to enrolment, and should be kept constant during the study unless medical reasons dictate otherwise.
**Criteria for evaluation (study period)**	
**- Effect parameters**	– Dystrophin expression in muscle biopsy<br>– Exon skip efficiency (RT-PCR on dystrophin mRNA from muscle biopsy and mononuclear blood cells)<br>– Muscle function (timed tests and 6-minutes walk)<br>– Muscle strength (QMT including respiratory muscles, MMT)
**- Safety parameters**	– Adverse events<br>– Local tolerability<br>– Safety biochemistry and hematology parameters<br>– Coagulation (aPTT)<br>– Urinalysis<br>– Complement split products (C3a/SC5b-9/Bb)<br>– Cytokines (IL-6, TNF-α) and chemokine (MCP-1)<br>– Antibodies to dystrophin<br>– ECG parameters
**- Pharmacokinetic parameters**	– $T_{1/2}$<br>– AUC: 0-24h, 24h-7d, 0-∞<br>– C_{max}, $C_{trough, 7d}$<br>– t_{max}<br>– volume of distribution and clearance
**Criteria for evaluation (treatment beyond study period)**	
**- Effect parameters**	– Dystrophin expression in muscle biopsy<br>– Exon skip efficiency (RT-PCR on dystrophin mRNA from muscle biopsy and mononuclear blood cells)<br>– Muscle function (timed tests and 6-minutes walk)<br>– Muscle strength (handheld myometry and spirometry)

**Protocol number**	PRO051-02
**EudraCT number.**	2007-004819-54
**- Safety parameters**	– Adverse events<br>– Local tolerability<br>– Safety biochemistry and hematology parameters<br>– Coagulation (aPTT)<br>– Cystatin C (in blood)<br>– Urinalysis (including Urine cystatin C, KIM-1, α-1 microglobulin, protein/creatinine ratio)<br>– Complement split products (C3)<br>– Cytokines (IL-6, TNF-α) and chemokine (MCP-1)<br>– Antibodies to dystrophin<br>– ECG parameters
**- Pharmacokinetic parameters**	– $T_{1/2}$<br>– AUC: 0-24h, 24h-7d, 0-∞<br>– C_{max}, $C_{trough, 7d}$<br>– t_{max}<br>– volume of distribution and clearance
**Statistical Methods**	All effect and safety data will be listed and summarized. A formal statistical analysis will not be conducted. Data will be scrutinized for effect and signs of potential safety or tolerability issues.<br>Plasma concentration versus time profiles of PRO051 will be analyzed to calculate the pharmacokinetic parameters. Linearity of PRO051 pharmacokinetics will be evaluated using ANOVA on log-transformed, dose normalized PK parameters.
**Interim analyses**	Not applicable
**Schedule of assessments**	See Appendix 2

N.B.GlaxoSmithKline will take sponsorship for the study on 21 July 2011 and will supply study drug from that date onwards. For simplicity, the protocol will continue to refer to the drug as PRO051 (PRO051 = GSK2402968).

Table of contents

Protocol synopsis 2

Table of contents 6

List of abbreviations and definition of terms 8

Study addresses 9

Sponsor signature page 10

Signature of principal investigator 11

Signature of co-investigator/sub-investigator 12

1 Introduction 13

1.1 Background on Duchenne muscular dystrophy 13

1.2 Therapeutic concept of exon skipping 13

1.3 Rationale for exon skipping in Duchenne muscular dystrophy 13

1.4 Dose justification 18

2 Study objective(s) 19

3 Study design 20

3.1 Overall study design 20

3.2 Study treatments 21

3.3 Study evaluations 22

4 Subject selection 26

4.1 Subject population 26

4.2 Inclusion criteria 26

4.3 Exclusion criteria 26

4.4 Concomitant medication/therapies 26

4.5 Subject withdrawal criteria 27

5 Study plan 28

5.1 Screening procedures 28

5.2 Effect parameters during study period 30

5.3 Safety parameters during study period 32

5.4 Pharmacokinetic parameters during study period 34

5.5 Effect parameters during treatment beyond study period 35

5.6 Safety parameters during treatment beyond study period 37

5.7 Pharmacokinetic parameters during treatment beyond study period 43

5.8 Compliance with the protocol 43

5.9 Termination of the study 44

6 Study medication 45

6.1 Study agents 45

6.2 Study medication manufacture, packaging, labeling and storage 45

6.3 Study medication administration 45
6.4 Discontinuation of study medication 46
6.5 Study medication accountability 46

7 Adverse event reporting and follow up 47

7.1 Definitions 47
7.2 Reporting of adverse events 48
7.3 Reporting of serious adverse events 50
7.4 Adverse event follow up 50

8 Data management, statistical analysis and reporting 51

8.1 Recording of data 51
8.2 Study monitoring 51
8.3 Safety monitoring 51
8.4 Data management 52
8.5 Statistical methods 52
8.6 Study report 55

9 Administrative procedures 56

9.1 Basic principles 56
9.2 Ethical considerations 56
9.3 Financial disclosure 57
9.4 Publication policy 57
9.5 Record retention 58
9.6 Protocol amendments 58
9.7 Quality control and quality assurance 58
9.8 Regulatory inspections 58

10 References 59

Appendix 1 Declaration of Helsinki 61

Appendix 2 Schedules of assessments 64

Appendix 3 Parameters for routine hematology, biochemistry and urinalysis assessment 84

Appendix 4 Analytical Methods 85

List of abbreviations and definition of terms

AE	Adverse Event
ALAT	Alanine Aminotransferase
ALP	Alkaline Phosphatase
AON	Antisense OligoriboNucleotide
aPTT	Activated Partial Thromboplastin Time
ASAT	Aspartate Aminotransferase
Bb	Split fragment of complement factor B
BMC	Bone Mineral Content
BMD	Becker Muscular Dystrophy
BP	Blood Pressure
C3a	Split fragment of complement factor C3
CK	Creatine Kinase
CRF	Case Report Forms
CRO	Contract Research Organisation
CRP	C-Reactive Protein
DEXA	Dual Energy X-ray Absorptiometry
DLT	Dose Limiting Toxicity
DMC	Data Monitoring Committee
DMD	Duchenne Muscular Dystrophy
ECG	Electrocardiogram
FEV_1	Forced Expiratory Volume in the 1st second of exhalation
FVC	Forced Vital Capacity
FTM	Fat Tissue Mass
γ-GT	Gamma Glutamyl Transferase
GLDH	Glutamate dehydrogenase
HR	Heart Rate
IB	Investigator’s Brochure
ICH-GCP	International Conference on Harmonisation – Good Clinical Practice
IDMC	Independent Data Monitoring Committee
IEC	Independent Ethics Committee
IL-6	Interleukin-6
INR	International Normalized Ratio
ISAB	Internal Safety Assessment Board
ITT	Intention to Treat
LDH	Lactate DeHydrogenase
LTM	Lean Tissue Mass
LUMC	Leiden University Medical Centre
MCH	Mean Corpuscular Hemoglobin
MCHC	Mean Corpuscular Hemoglobin Concentrations
MCP-1	Monocyte Chemotactic Protein-1
MTD	Maximum Tolerated Dose
NOAEL	No Adverse Effect Level
OTC	Over The Counter
PB-MNC	Peripheral blood mononuclear cells
PTT	Partial Thromboplastin Time
RT-PCR	Reverse Transcription-Polymerase Chain Reaction
SAE	Serious Adverse Event
SC5b-9	Split fragment of complement factor C5
SD	Standard Deviation
SPM	Study Procedures Manual
TNF-α	Tumor Necrosis Factor-α
ULN	Upper Limit of Normal
KIM-1	Kidney Injury Molecule-1

Study addresses

Sponsor primary contact	[redacted]
Contact for reporting Serious Adverse Events	[redacted]
Sponsor contact for Adverse Events (Medical Officer)	[redacted]
Coordinating investigator	[redacted]
CRO for Monitoring	[redacted]
Central Laboratory	[redacted]

Sponsor signature page

A phase I/II, open label, escalating dose, pilot study to assess the effect, safety, tolerability and pharmacokinetics of multiple subcutaneous doses of PRO051 in patients with Duchenne muscular dystrophy

On behalf of the sponsor

GlaxoSmithKline
Iron Bridge Road
Stockley Park West, Uxbridge, Middlesex, UB11 1BU, UK
Telephone: [redacted]

[redacted]

6/6/2012
Date

Vice President Clinical

GSK Rare Diseases

[redacted]

Clinical Research Manager
On behalf of Prosensa Therapeutics B.V.

[redacted]

18/6/2012
Date

Signature of principal investigator

A phase I/II, open label, escalating dose, pilot study to assess the effect, safety, tolerability and pharmacokinetics of multiple subcutaneous doses of PRO051 in patients with Duchenne muscular dystrophy

By my signature below I agree to conduct this clinical trial in accordance with the protocol, ICH-GCP, the Declaration of Helsinki, government regulations and state/local customs or laws, including those applying to institutional/ethics review and informed consent.

I agree to the terms of this study protocol

____
Centre

____
Name (print)

____ ________________
Signature Date

Copyright 2011 the GlaxoSmithKline group of companies. All rights reserved. Unauthorised copying or use of this information is prohibited.

Signature of co-investigator/sub-investigator

A phase I/II, open label, escalating dose, pilot study to assess the effect, safety, tolerability and pharmacokinetics of multiple subcutaneous doses of PRO051 in patients with Duchenne muscular dystrophy

By my signature below I agree to conduct this clinical trial in accordance with the protocol, ICH-GCP, the Declaration of Helsinki, government regulations and state/local customs or laws, including those applying to institutional/ethics review and informed consent.

I agree to the terms of this study protocol

Centre

Name (print)

Signature

Date

Copyright 2011 the GlaxoSmithKline group of companies. All rights reserved. Unauthorised copying or use of this information is prohibited.

1 Introduction

1.1 Background on Duchenne muscular dystrophy

Duchenne muscular dystrophy (DMD) is the most frequent inheritable lethal childhood's disease with an incidence of 1 in 3,500 newborn boys[1]. In DMD, boys begin to show signs of muscle weakness as early as age 2. The disease gradually weakens the skeletal muscles in arms, legs and trunk. By the early teens or even earlier, the boys' heart and respiratory muscles will also be affected. Most DMD patients are wheelchair-bound before the age of 12 and prior to the introduction of ventilatory support died typically around the age of twenty years. To date, no effective, clinically applicable, treatment is available. A variety of therapeutic strategies for DMD have been studied, including muscle or stem cell transplantation, pharmacological compounds, and gene construct addition through viral or non-viral delivery of dystrophin-encoding sequences to muscle tissue. However, the large size of the DMD gene, the physical properties of muscle tissue, and immunological effects have hindered straightforward progress towards clinical applications.

Dystrophin is an essential component of the dystrophin-glycoprotein complex maintaining the membrane integrity of muscle fibers[2,3]. The absence of dystrophin induces a continuous loss of muscle fibers, which eventually causes premature death in adolescence[4-6].

Many aberrations in the DMD gene have been identified, in particular deletions of one or more exons (~60%), but also duplications (~6%), translocations and point mutations have been found[7]. In general, mutations that disrupt the translational reading frame of the dystrophin transcript, lead to a prematurely aborted dystrophin synthesis, and thus cause DMD.

A milder form is detected in a number of patients who have in-frame deletions in the DMD gene, resulting in a shorter form of the protein which is still partly functional and subsequently leads to relatively mild clinical symptoms[2,8]. This form is known as Becker Muscular Dystrophy (BMD) and these patients show a better life expectancy and quality of life when compared to the DMD patients. This indicates that part of the dystrophin protein is not essential for its functions.

1.2 Therapeutic concept of exon skipping

Antisense-induced exon skipping is gaining attention as a novel and promising tool for the restoration of the reading frame. The aim is to manipulate splicing in such a manner that the targeted exon is skipped (through binding of the AONs to pre-mRNA) and a slightly shorter but in-frame transcript is generated. This would allow the synthesis of a BMD-like dystrophin protein that may significantly alleviate progression of the disease (Figure 1).

1.3 Rationale for exon skipping in Duchenne muscular dystrophy

1.3.1 Non-clinical pharmacology data with exon skipping

Several non-clinical studies have shown the therapeutic potential of the exon skipping strategy for restoring dystrophin production in cultured patient-derived muscle cells *in vitro*, and in *mdx* mouse muscle tissue *in vivo* (*mdx* mice are dystrophin-deficient)[9-13]. In these studies, AONs were targeted either to splice site consensus sequences (involved in removing non-coding sequences (introns) from the pre-mRNA) or to exon-internal sequences. In particular, it has been demonstrated that the binding of an oligoribonucleotide to exon-internal sequences *per se* is sufficient to induce exon skipping, that many exons in the DMD gene are "skippable", and thus that exon skipping, in principle, is applicable to the majority of mutations (>90%), including deletions, duplications, or nonsense mutations. In human muscle cells LUMC has identified a set of human-specific AONs efficiently mediating the skipping of 39 different DMD exons: exons 2, 8, 17, 19, 29, 33, 40 to 64, and 71 to 78. The broad therapeutic potential of AONs was shown in cultured muscle cells from a series of

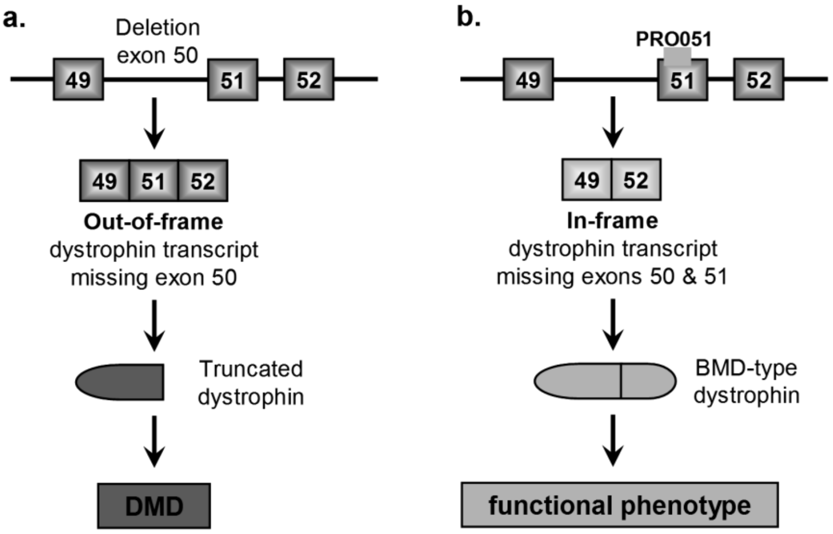

Figure 1. Schematic representation of the exon-skipping strategy. ***a*** *In DMD patients with a deletion of exon 50, an out-of-frame transcript is generated in which exon 49 is spliced to exon 51. This results in a frame-shift and prematurely aborted dystrophin synthesis.* ***b*** *Antisense oligoribonucleotide PRO051, directed to an exon-internal sequence in exon 51, can induce the skipping of this exon. Accordingly, the transcript is back in-frame and a BMD-like dystrophin can be synthesized.*

DMD patients affected by different mutations[9,10,14,15]. In all cases the targeted exon was skipped specifically and with relatively high levels of up to ~90%, which induced the synthesis of significant levels of dystrophin in over 75% of treated cells. These dystrophins located appropriately to the sarcolemma and restored the dystrophin-glycoprotein complex, a strong indication of functional restoration.

Moreover, the high sequence-specificity of the exon skipping strategy has been demonstrated when exon-internal sequences are used. For this reason, such AONs are expected to be safe therapeutic agents with respect to off-target effects.

In two systemic *in vivo* studies[11,12], AONs were applied intravenously in *mdx* mice, a DMD mouse model carrying a nonsense mutation in exon 23. First, an exon 23 skipping 2OMePS AON (20-mer (+02-18)) was administered through the tail vein[12], at a dose of 2 mg per injection (~100 mg/kg). After three weekly injections, dystrophin expression up to 1%-5% of normal was detected in all muscle groups analyzed except heart, with highest levels in the gastrocnemius, intercostal muscles and the diaphragm. Analysis of blood parameters and histology of liver, kidney, lung and muscles did not reveal any tissue damage caused by the 2OMePS treatment.

In a second study[11], a similar but longer and overlapping AON sequence (25-mer, (+07-18)) was applied with a modified chemical (morpholino) structure. After seven weekly intravenous injections of an equal dose of AON (2 mg per injection) in 6 weeks old *mdx* mice, dystrophin protein was detected at levels up to 50% of normal in gastrocnemius and quadriceps muscles, and up to 10-20% in tibialis, intercostal, abdominal and triceps muscles. The significant force improvement of the tibialis anterior muscle, with only <20% dystrophin levels, and the reduced serum creatine kinase levels in treated mdx mice, indicated that the dystrophin-restoring treatment indeed significantly restored the membrane integrity and function of dystrophic myofibers.

In a preliminary pilot study, LUMC and Prosensa have recently employed subcutaneous administration of the exon 23 2OMePS AON (20-mer (+02-18)) in series of mdx mice. Mice were treated for either 4 or 8 weeks, with two subcutaneous injections of 100 mg/kg per week. Accumulating exon skipping levels were observed in body-wide skeletal muscle

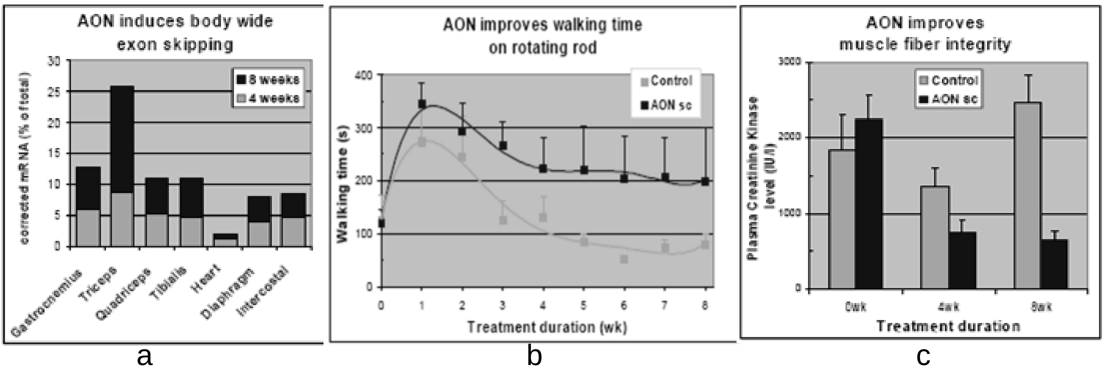

a b c

Figure 2. Subcutaneous administration of the exon 23 2OMePS AON (20-mer (+02-18)) in series of mdx mice. ***a*** *Exon skipping levels in body-wide skeletal muscle groups.* ***b*** *Walking time on rotating rod.* ***c*** *Muscle fiber integrity.*

groups (up to 25%), and in heart (2%) (Figure 2a). This resulted in novel dystrophin expression in all samples analyzed; up to 10% and 4% of control in the tibialis anterior and heart muscle respectively, after 8 weeks of treatment. In addition, improvement was observed in both muscle function, as shown by longer rotarod running durations, and muscle fiber integrity, as indicated by lower plasma creatine kinase levels (Figure 2b,c).

Towards the development of a human-specific protocol, LUMC has further applied the exon skipping strategy in transgenic hDMD mice carrying an integrated, complete copy of the full-length (2.3 Mb) human DMD gene that is expressed in their muscle tissue. This model uniquely allows pre-clinical validation of human sequence-specific exon skipping *in vivo*. In fact, it was possible to induce the skipping of various exons, including exons 44 and 51, from the human gene specifically in mouse muscle tissue *in vivo*[16].

In conclusion, pre-clinical data generated in cells derived from a series of DMD patients and in the *mdx* mouse model using different antisense oligoribonucleotides to induce exon skipping demonstrated that targeted exon skipping is a promising approach to restore dystrophin synthesis and improve muscle fiber integrity and function.

1.3.2 Non-clinical toxicology data with PRO051

As a class, the toxicity of oligonucleotides has been well characterized in laboratory animals and the dose-response and concentration-response relationships for the toxicities have been defined[17,18]. More than 3000 subjects have been dosed with antisense phosphorothioate oligonucleotide drugs and the relationships between non-clinical toxicities and their clinical manifestations are well understood[19]. Phosphorothioate oligonucleotides as a class have pharmacokinetic and toxicity profiles that are within relatively narrow limits similar from sequence to sequence. The basis for this consistency is that the interactions of oligonucleotides with plasma proteins, cell-surface receptor, or uptake and accumulation within cells are dependent on the physical chemical characteristics of the oligonucleotides. Pharmacokinetics in the typical laboratory animal models, particularly monkeys, are highly predictive of those in man, and doses can be extrapolated on the basis of body weights[20].

The most common effects identified for oligonucleotides in animal studies are presented below (Table 1) with their clinical relevance (or adverse events observed in past clinical studies) with first and second generation phosphorothioate oligonucleotides[17,19].

The pattern of toxicity observed with PRO051 shares many of the characteristics with other phosphorothioate oligonucleotides. The effects of PRO051 have been described below and a more detailed summary and discussion is provided in the Investigator's Brochure.

Table 1. General experience with phosphorothioate oligonucleotides

Class toxicities in animals	Clinically correlates with
Prolongation of aPTT<br>(due to protein binding in plasma)	Transient prolongation related to plasma level (no signs of increased bleeding or bruising)
Activation of the alternative complement pathway in monkeys	Not clinically significant in man
Renal tubular changes: granules and degeneration/regeneration (highest steady state tissue levels)	No renal effects observed to date
Pro-inflammatory effects<br>- systemic primarily in rodents<br>- site of administration in monkeys (cytokine release after activation of monocyte and/or dendritic cells)	Fever and chills at high doses or rapid infusion rates (especially for unmodified phosphorothioate oligonucleotides)<br>Injection site erythema
Thrombocytopenia<br>(sequestration)	Occasionally observed (sequence dependent & predicted by monkeys)
Liver has the largest amount and second highest tissue levels of oligonucleotides<br>Mild ALAT/ASAT increase in mice related to pro-inflammatory effects	Elevations in serum transaminases or other signs of hepatoxicity largely absent.<br>Incidental findings with likely alternative causes.

On the basis of acute and subchronic studies in monkeys, PRO051 prolongs the intrinsic clotting pathway (aPTT) and increases complement split products. Levels of split products only approach clinically significant levels at relatively high plasma concentrations, which are not expected to be reached at the proposed dose levels in the clinical protocol. These acute effects of treatment are transient and follow the PRO051 plasma profile. Due to rapid distribution, PRO051 plasma levels decline quickly after reaching peak levels at approximately 3h for subcutaneous administration to less than 1/10th of the maximum concentration at 24h. Following distribution, 2' modified oligonucleotides present in tissues are very stable with an estimated tissue half life of 1 week (kidney) to 4 weeks (liver) for PRO051. Trough plasma levels are less than 1/1000th of the maximum plasma concentration and decline with a terminal half life of 27 days. Indeed peak plasma levels of PRO051 and related acute effects do not appear to increase over repeated administration. However, there will be continued tissue exposure at once weekly dosing intervals.

The most prominent target organ findings for PRO051 in the monkey has been the presence of granules in the proximal tubular epithelial cells, most likely incorporating reabsorbed PRO051. Importantly, no signs of tubular degeneration were noted at the highest tested dose (140 mg/kg/wk). At the injection site, minimal pro-inflammatory changes have been observed in monkeys and these are accompanied by minimal transient increases in plasma MCP-1. No signs of systemic immunostimulatory effects and no thrombocytopenia have been observed in monkeys treated up to 140 mg/kg/wk. Systemic pro-inflammatory responses to PRO051 have been observed in rodents (≥4 mg/kg/wk in rats). At higher dose levels (≥175 mg/kg/wk for mice and ≥56 mg/kg/wk for rats), pro-inflammatory effects are associated with signs of hepatoxicity and in rats some renal dysfunction is also noted. Past clinical experience with phosphorothioate oligonucleotides, has demonstrated that the pro-inflammatory responses in rodents are less relevant to humans and results from monkey studies have been more predictive for man. Considering the aggressive dosing regimens used in these toxicity studies with PRO051, the toxicities observed for PRO051 are relatively mild compared to other phosphorothioate oligonucleotides in clinical studies[19]. This toxicity profile for PRO051 should encourage further development and advancement into longer duration clinical trials.

NOAEL levels for subcutaneous treatment with PRO051 have been established in the animal studies at 18 mg/kg/wk for monkeys and 18 mg/kg/wk for mice and in rats by intravenous

administration at 4 mg/kg/wk. It has been demonstrated for oligonucleotides that the pharmacokinetics between species extrapolates on the basis of body weight, rather than body surface area[20] Therefore, a species conversion factor is not required for calculating the starting dose in humans and this exception has been acknowledged in a FDA draft guidance document on estimating the maximum safe starting dose (FDA, 2005). In the previous clinical study, 0.8 mg (0.013-0.024 mg/kg) has been administered intramuscularly. The proposed dose level range for this study is 0.5-10 mg/kg/wk by subcutaneous administration. For the starting dose, this provides a safety margin of 35x compared to the NOAEL in monkeys and mice and 8x to in rats. These safety margins are considered appropriate and conservative, because (i) monkeys are considered more relevant than rodents, in particular the absolute pro-inflammatory effects in rodents have been less relevant for predicting dose limiting toxicities in humans for oligonucleotides; (ii) the subchronic monkey and mouse more closely mimic the intended clinical study in route and frequency of administration and duration of treatment; (iii) no test-item related systemic adverse event has been observed in the previous clinical study and a safety margin of >10x compared to the monkey NOAEL, the most relevant species, is considered sufficiently appropriate for a study in this population (DMD patients of 5-16 years).

Based on preliminary plasma pharmacokinetic data and non-compartmental analysis in monkeys, the maximum plasma concentration should remain below 150 µg/ml to limit acute effects. The AUC0-7d is approximately 920 µg.h/ml based on the NOAEL of 18 mg/kg/wk. In the proposed clinical study employing subcutaneous administration, the maximum dose of 10 mg/kg is expected to yield plasma peak concentration of approximately 50 µg/ml and an AUC0-7d of approximately 511 µg.h/ml.

Local reactions to subcutaneous injection (up to 250 mg) during the clinical study may occur, but these are not expected to be dose limiting. Past experience, especially with the first generation phosphorothioate oligonucleotides, indicate that fever and chills may be dose limiting toxicities. These reactions have been dose dependent, transient and well treatable.

1.3.3 Clinical data with PRO051

Study PRO051-01 assessed the local dystrophin production restoring effect and safety and tolerability of a single intramuscular dose of 0.8 mg PRO051 in Duchenne muscular dystrophy patients. This study showed for the first time efficient restoration of dystrophin expression in muscle of four DMD patients. Each patient showed specific skipping of exon 51 and sarcolemmal dystrophin in 64% to 97% of myofibers[21]. The treatment was well tolerated, most frequently reported adverse events were related to the muscle biopsy rather than to PRO051 administration. Only one adverse event possibly related to study drug administration was recorded, i.e. mild local pain after intramuscular injection.

From this study it was concluded that PRO051 induces dystrophin protein synthesis when applied intramuscularly in DMD patients with suitable mutations. Also, PRO051 is safe and well-tolerated at this dose level and route of administration.

Refer to Investigator Brochure for summary on PRO051 results.

1.3.4 Rationale for development

LUMC has optimized target sequences within exon 51. The most effective antisense oligoribonucleotide to induce the skipping of exon 51 is named PRO051. It is highly sequence-specific, exhibiting no 100% homology to any other sequence in the human genome and is sensitive for mismatches. Its therapeutic efficiency in cultured muscle cells from a series of DMD patients affected by a deletion of exons 45-50, exons 48-50, exons 49-50, exon 50, or exon 52 has been demonstrated[10,21]. In addition, its molecular therapeutic effect, i.e. exon 51 skipping and dystrophin restoration, has also been shown in a clinical setting following local administration of PRO051 into the muscle[21].

1.4 Dose justification

Subcutaneous administration is considered to have similar potency as the intravenous route, but has higher patient convenience and is less susceptible to plasma peak level related adverse events. As PRO051 is very stable with a long half life (as determined in monkeys), a weekly administration is sufficient to obtain continuous tissue exposure. The minimum and maximum dose levels to be applied are based on the results from the various animal studies investigating the toxicity and/or efficacy of PRO051 (please refer to the Investigator’s Brochure for further details on the results from those animal studies).

2 Study objective(s)

To preliminarily assess the effect of PRO051 at different dose levels in patients with Duchenne muscular dystrophy

To assess the safety and tolerability of PRO051 at different dose levels in patients with Duchenne muscular dystrophy

To determine the pharmacokinetics of PRO051 at different dose levels after subcutaneous administration in patients with Duchenne muscular dystrophy.

3 Study design

3.1 Overall study design

This study has been designed as an open label, multiple dose, escalating dose study. Depending on the MTD level, twelve to eighteen patients will be studied in four groups.

The study flow chart in Figure 3 outlines the dose escalation design of this study. Patients in the first group will receive 0.5 mg/kg, in the second group maximally 2 mg/kg, in the third group maximally 6 mg/kg and in the fourth group maximally 10 mg/kg.

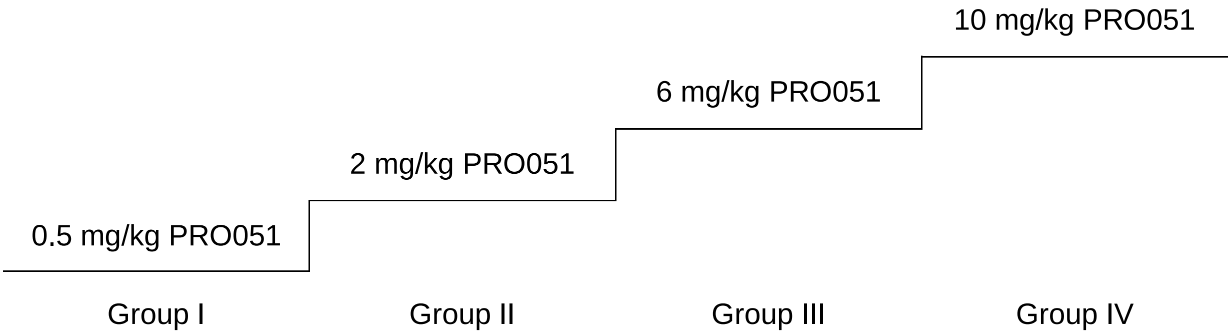

Figure 3. Study flow chart with maximum dose levels to be administered.

Patients will receive one dose of PRO051 per week for 5 weeks. Each group will initially consist of 3 patients. Interim safety review for dose escalation will take place after review of all safety data that have been collected from at least up to and including the first three administrations of study medication (i.e. at least up to and including visit 4) for each patient and no prohibitive adverse events have been observed. In case of potentially dose limiting toxicity (DLT) in one of three patients in one group, this group will be expanded to six patients. In case of similar dose limiting toxicity in two or more patients, the inclusion of patients will be stopped at that dose level. Further, it will be decided if inclusion of new subjects should be stopped, or if the study should continue with a new lower dose group, or if the study should continue by expanding the previous group to six subjects. The dose level of the group which has a dose level one step below the group with similar dose limiting toxicity in two or more patients, will be considered the maximum tolerated dose (MTD).

The decision model for discontinuation or modification of dose escalation schedule is shown in Table 2.

Given the limited number of patients affected by DMD correctable by exon 51 skipping, an adaptive design was chosen to ensure that at least at the highest dose levels a reasonable number of patients could be included and that sufficient data on dose limiting toxicities could be collected. Furthermore, it was considered that the safety and tolerability profile warranting further investigations in a larger population could sufficiently be established with these group sizes.

No control group was included due to the limited number of patients available.

Patients will receive one subcutaneous treatment of PRO051 per week for a period of five weeks (i.e. five injections in total). This number of injections was considered sufficient to establish the MTD and a dose level to investigate in future studies prior to embarking on trials with longer treatment duration. After the five weeks of treatment period, the patients will continue with follow up visits till 13 weeks after the last treatment.

Table 2. Decision model for discontinuation or modification of dose escalation schedule[a]

Group	DLT		
If # of subjects is	And if # of subjects with similar DLT is		Action
3	0	Then	Continue with next higher dose group
3	1	Then	Expand group to 6 subjects
3	2 or more	Then	Determine if:<br>– this group should be expanded to 6 subjects<br>– previous group should be expanded to 6 subjects<br>– a new group should start with a lower dose[b]<br>– study should be stopped
6	1	Then	Continue with next higher dose group
6	2 or more	Then	Determine if:<br>– study should be stopped<br>– study should continue with next group and determine dose level for that group[c]

a. The nature of the DLT will be taken into account

b. Depending on the nature of the DLT, the next group can in principle receive an intermediate dose between the current dose level and the previous dose level

c. Depending on the nature of the DLT, the next group can in principle receive an intermediate dose between the current dose level and the previous dose level

3.2 Study treatments

3.2.1 Treatments administered

Successive cohorts of 3-6 patients will receive PRO051 by subcutaneous injection on day 1, 8, 15, 22 and 29. The number of injection sites per administration depends on the total dose to be administered but will not exceed two injection sites.

Patients in the first group will receive 0.5 mg/kg, in the second group maximally 2 mg/kg, in the third group maximally 6 mg/kg and in the fourth group maximally 10 mg/kg.

Dose escalation will only take place after review by the investigators and the medical responsible person of the sponsor as described in section 3.1.

If review of the safety data raises concerns about the next dose level to be administered, the dose level may be adjusted downwards. If this occurs, the IEC/IRB and regulatory authorities if applicable will be notified of changes in planned dose levels.

3.2.2 Selection of doses administered

The dose levels for the escalating dose levels were selected on the basis of available animal toxicological data and past clinical experience with chemically related phosphorothioate oligonucleotides (see also section 1.3 and 1.4).

3.2.3 Selection and timing of dosing for each patient

Study medication will be administered five times to each patient with an interval of one week between administrations. Injection will preferably be given in the morning and always at approximately the same time of the day.

3.2.4 Method of assigning subjects to treatment groups

This is an open study in which all study subjects will receive active treatment. Patients will be assigned to a dose level based on the sequence of entering the study.

For safety reasons, there will be an interval of at least 2 days between the start of treatment of each patient within one group.

3.2.5 Blinding

Not applicable.

3.2.6 Treatment compliance

Administration of study medication and any deviation thereof will be recorded on the appropriate CRF.

3.3 Study evaluations

Appendix 2 depicts the schedules of assessments throughout the study. Table 4 shows the assessments to be done during the study period (visit 1 – 12). Table 5 specifies the assessments to be done during each hospitalization visit (visit 2 – 6). In Tables 6 - 14 is depicted the schedule of assessments to be done during the treatment beyond study.

Total blood volume during the study period to assess effect, safety laboratory and pharmacokinetic parameters is maximally 200 ml for each study participant, divided over 13 visits during a 18-week (4 ½ months) period. Main burden will be during the 5-week treatment period.

In the treatment beyond study period, the total blood volume to assess effect, safety laboratory and pharmacokinetic parameters is for each study participant as follows.

- maximally 140 ml during the period of the 1st week visit u/I the 13th week visit. Main burden will be at week 1, 5, 9, and 13.
- maximally 125 ml during the period of the 14th week visit u/I the 25th week visit. Main burden will be at week 17, 21, and 25.
- maximally 135 ml during the period of the 26th week visit u/I the 49th week visit. Main burden will be at week 29, 33, 37, 41, 45, and 49.
- maximally 122 ml during the period of the 50th week visit u/I the 73rd week visit. Main burden will be at week 53, 57, 61, 65, 69, and 73.
- maximally 157 ml during the period of the 74th week visit u/I the 97th week visit. Main burden will be at week 77, 81, 85, 89, 93, and 97.
- maximally 157 ml during the period of the 98th week visit u/I the 121nd week visit. Main burden will be at week 101, 106, 109, 113, 118 and 121.
- maximally 157 ml during the period of the 122nd week visit u/I the 145th week visit. Main burden will be at week 125, 130, 133, 137, 142, and 145.
- maximally 157 ml during the period of the 146th week visit u/I the 169th week visit. Main burden will be at week 148, 154, 157, 161, 166 and 169.
- maximally 157 ml during the period of the 170th week visit u/I the 193rd week visit. Main burden will be at week 172, 177, 180, 184, 189 and 192.
- maximally 157 ml during the period of the 194th week visit u/I the 217th week visit. Main burden will be at week 196, 201, 204, 208, 213 and 216.

All remaining test material (clinical trial samples) will be stored under the required storage conditions for at least 3 months after completion of the Analytical Report, with a maximum of one year. Destruction will be authorized, documented and archived. However, additional

written consent has been obtained for unlimited storage and future use of biological samples for medical scientific research.

3.3.1 Treatment period

Treatment visits take place on day 1, 8, 15, 22 and 29. Subjects will be admitted to the hospital for an overnight stay on the treatment days. Baseline assessments will be done at time point 3 to 0 hours prior to dosing. Then PRO051 is administered by a maximum of 2 subcutaneous injections (depending on the total dose to be administered) in the abdomen located at least 4 cm apart (t=0 day 1, 8, 15, 22 and 29). Subjects will be monitored for approximately 30 hours (one night), during which all safety and laboratory assessments, adverse events and other assessments as indicated in the Schedule of assessments will be completed (Appendix 2: Table 5). Thereafter, the subjects can return home. On the third day after discharge (i.e. t=96±6 hrs after dosing) patients will be contacted by telephone by the investigator to enquire about adverse events and concomitant medication.

3.3.2 Follow-up period during study

Follow-up visits take place on Days 36, 43, 57, 78, 99 and 120.

Assessments as indicated in the Schedule of assessments (Appendix 2: Table 4) will be made during each of these follow-up assessments.

3.3.3 Treatment beyond study period

Provided patient and/or his parents, the investigator and the sponsor all agree that drug administration appears to improve the clinical status of the patient, patients will be offered the option to continue treatment with weekly or alternative dose administrations.

Thus, when subject has completed the treatment and follow up period of the study, treatment with PRO051 may be restarted. All subjects in the treatment beyond study period were started on a dose of 6 mg/kg PRO051. Following review of the ongoing treatment beyond study phase, subclinical nephrotoxicity (mild proteinuria) has been observed after 6 months of treatment in a number of subjects, but particularly in one subject. This subject has the highest weight (62 kg) and therefore also receives the highest dose (366 mg). The subject also had increases in albumin and α1-microglobulin and experienced significant injection site reactions (which are probably related to volume of injective).

It is therefore considered appropriate at this point to start to explore alternative dosing options with the aim of minimizing safety risks while maintaining efficacy. Pharmacokinetic, body fat composition (DEXA) and safety data have all been reviewed, but because of the small group size (12 subjects) the relationship between exposure, lean muscle mass and safety cannot be quantified at this time.

Therefore, it is proposed that initially a weight cap on dosing will be implemented, whereby subjects with a body weight up to 50 kg will continue to be dosed at 6 mg/kg but subjects with a body weight higher than 50 kg will receive a fixed maximal dose of 300 mg. Based on current renal parameter data, capping the dose at a maximum of 300 mg may provide better longer-term safety and prevent subjects from reaching renal (and other) stopping criteria. In addition, this data will be useful in determining the optimal dosing strategy in the long-term for this compound.

Additionally, it is considered appropriate to introduce a drug interruption of 8 weeks for these subjects. This is designed to allow a drug washout of non-target organs including the liver and kidney. It is anticipated that this drug interaction will not compromise efficacy due to retention of drug in muscle and the long half life of dystrophin

All subjects will have an 8 week interruption to drug administration, following completion of Visit 85 (or as soon as Amendment 7 is approved after Visit 85). During the 8 weeks off drug, subjects will be asked to return for Visit 89 where safety parameters and efficacy measures will be made. Additional visits can be arranged depending on the current clinical status of the

subject and the clinical judgement of the investigator. Subjects will then re-start study administration according to a dosing regimen of;

- Subjects will receive one dose of 6 mg/kg PRO051 per week x 8 weeks if their body weight is ≤50 kg. This will be followed by 4 weeks off drug. This will continue in a cyclical manner.
- If a subjects body weight >50 kg, he will receive one maximal dose of 300 mg PRO051 per week x 8 weeks This will be followed by 4 weeks off drug. This will continue in a cyclical manner.

Based on PK/PD modelling this regimen is predicted to result in an overall 12-week exposure that is somewhat lower than the continuous dosing regimen. The dystrophin levels are predicted to fluctuate between about 70% of the current level at the end of the off drug period, to about 80% at the end of the 8 weeks on drug. Although a compromise in efficacy is not anticipated, the impact on response will be closely monitored and if a perceived continuous decline in efficacy is observed, the investigator will discuss the potential benefit for an individual subject to return to continuous weekly dosing at 6mg/kg PRO051 with the Medical Monitor where the safety and tolerability is acceptable. The intermittent dosing regimen may, however, help to minimize organ burden from accumulation of this compound in organs, such as liver and kidney by allowing organs to washout during the off-treatment period. The overall risk benefit profile will be regularly assessed. Any change from intermittent dosing to continuous weekly dosing will be implemented at the start of a 12 week cycle.

The treatment with PRO051 beyond the study period should comply with the following conditions:

- Subject benefits from the PRO051 treatment
- Safety and effect assessments are performed as described in Appendix 2: Table 6, Table 7, Table 8 and Table 9.
- Maximum treatment period is until (whichever comes first):
  - the start of a next trial with PRO051 for which the subject is eligible, or
  - is exposed to safety risks outweighing potential benefits, or
  - PRO051 no longer appears to improve or stabilize the clinical status of the patient, or
  - development program of PRO051 is halted or discontinued, or
  - marketing authorization of PRO051 has been obtained, or
  - sponsor is not able to supply PRO051 anymore, or
  - sponsor decides to stop further clinical development of PRO051 (taking into account a reasonable term of notice), or
  - sponsor decides to stop further production of PRO051 (taking into account a reasonable term of notice).
- If there are possibilities for reimbursement of PRO051 during the treatment beyond study period but prior to marketing authorization, sponsor may ask the support of investigator to help in arranging for such reimbursement, if feasible.

3.3.4 Efficacy evaluations

The following parameters will be used to evaluate efficacy: dystrophin expression in muscle biopsy, PRO051 levels in muscle biopsy, mRNA production in peripheral blood mononuclear cells, timed tests (10-meter walk/run test, timed rising from floor, stair climb), 6-minute walk test, Quantitative Muscle Testing (QMT) and Manual Muscle Testing (MMT).

These evaluations will be performed at visits as shown in Appendix 2.

3.3.5 Safety evaluations

The following parameters will be used to evaluate safety: the assessment of AEs that may have occurred between each of the visits, physical examination, vital signs, ECG, routine biochemistry (sodium, potassium, calcium, urea, creatinine, ASAT, ALAT, γ-GT, LDH, ALP, CK, bilirubin, amylases, total protein, albumin, glucose, cholesterol), routine hematology (hemoglobin, MCV, erythrocyte count, hematocrit, MCH, MCHC, reticulocyte count, thrombocyte count, leukocyte count, leukocyte differential count: basophils, eosinophils, lymphocytes, monocytes, neutrophils), coagulation (aPTT), urinalysis (dipstick and quantitative protein and creatinine), complement activation (C3a, SC5b-9 and Bb), inflammatory response (IL-6, TNF-α and MCP-1) and immune response (dystrophin antibodies in blood), local tolerability at the injection site(s).

These evaluations will be performed at visits as shown in Appendix 2.

3.3.6 Pharmacokinetic evaluations

The pharmacokinetics of PRO051 (AUC, C_{max}, C_{trough}, t_{max}, clearance, volume of distribution and elimination half-life) will be evaluated.

Plasma samples for these evaluations will be collected at visits as shown in Appendix 2.

4 Subject selection

4.1 Subject population

Twelve to eighteen male patients aged at least 5 and not older than 16 years on the first day of drug administration with Duchenne muscular dystrophy resulting from an exon deletion correctable by treatment with PRO051.

4.2 Inclusion criteria

1. Boys aged between 5 and 16 years inclusive.
2. Duchenne muscular dystrophy resulting from a mutation correctable by treatment with PRO051.
3. Not ventilator dependent.
4. Life expectancy of at least 6 months.
5. No previous treatment with investigational medicinal treatment within 6 months prior to the study.
6. Willing and able to adhere to the study visit schedule and other protocol requirements.
7. Written informed consent signed (by parent(s)/legal guardian and/or the patient, according to the local regulations).

4.3 Exclusion criteria

1. Aberrant RNA splicing and/or aberrant response to PRO051, detected by *in vitro* PRO051 assay during screening.
2. Known presence of dystrophin in ≥ 5% of fibers in a pre-study diagnostic muscle biopsy.
3. Severe muscle abnormalities defined as increased signal intensity in >50% of the tibialis anterior muscle at MRI.
4. FEV_1 and/or FVC < 60% of predicted.
5. Current or history of liver or renal disease.
6. Acute illness within 4 weeks prior to treatment (Day 1) which may interfere with the measurements.
7. Severe mental retardation which in the opinion of the investigator prohibits participation in this study.
8. Severe cardiac myopathy which in the opinion of the investigator prohibits participation in this study.
9. Need for mechanical ventilation.
10. Creatinine concentration above 1.5 times the upper limit of normal (age corrected).
11. Serum ASAT and/or ALAT concentration(s) which suggest hepatic impairment.
12. Use of anticoagulants, antithrombotics or antiplatelet agents.
13. Subject has donated blood less than 90 days before the start of the study.
14. Current or history of drug and/or alcohol abuse.
15. Participation in another trial with an investigational product.

4.4 Concomitant medication/therapies

Anticoagulants, antithrombotics and antiplatelet agents are not allowed during the study. Other concomitant medication including glucocorticosteroids is allowed. However, if possible medication usage should not change during the study. Medication (including OTC medication, homeopathic remedies and herbal preparations) used and changes in the use of medication should be carefully recorded in the CRF.

Glucocorticosteriod use should be stable for at least 2 months prior to enrolment, and should be kept constant during the study unless medical reasons dictate otherwise.

4.5 Subject withdrawal criteria

A withdrawal is a subject who stops prematurely (for any reason).

In case a serious adverse event related to the study medication occurs, the subject must be withdrawn, unless doing so would harm the subject in the opinion of the investigator.

In case study medication is discontinued (for any reason), the subject may be withdrawn from the study.

If, during the course of the study, there is deterioration in the subject's wellbeing or the investigator feels that it is in the subject's best interest to be withdrawn from the study, the subject can be withdrawn.

The subject has the right to withdraw from the study at any time and for any reason, without affecting his future management and treatment. Reasons for subject withdrawal will be documented on the CRF.

4.5.1 Withdrawal during study period

A clear, concise reason should be recorded in the CRF for any subject prematurely ending the study. In addition, all subjects discontinuing should have a final visit including physical examination, vital signs measurement, ECG, safety biochemistry and hematology lab, urinalysis, dystrophin antibodies sample, pharmacokinetic sampling, muscle biopsy (if not yet collected at the time points as described in Table 4 in Appendix 2), muscle function and muscle strength (including FVC/FEV_1 assessment) measurement.

In case a complete final visit is not possible, at least all safety assessments should be performed. All study data from withdrawals should be retained.

4.5.2 Withdrawal during treatment beyond study period

A clear, concise reason should be recorded in the CRF for any subject prematurely ending the study. In addition, all subjects being withdrawn from the study should have a final visit including the following assessments (depending on the timing of withdrawal)

- Withdrawal at any time: physical examination, vital signs, safety biochemistry and hematology lab, urinalysis (dipstick, quantitative, alpha-1-microglobulin), cystatin C, muscle function and muscle strength (including spirometry assessment) measurement.
- Withdrawal prior to visit 37: aPTT, complement factor C3, pharmacokinetic sampling, muscle biopsy, ECG, dystrophin antibodies sample.
- Withdrawal prior to visit 61: ECG, dystrophin antibodies sample.
- Withdrawal prior to visit 85: dystrophin antibodies sample.

In case a complete final visit is not possible, at least all safety assessments should be performed. All study data from withdrawals should be retained.

5 Study plan

All equipment used for assessments of effect, safety and pharmacodynamic variables should be calibrated and maintained at least yearly. Documentation regarding the calibration and maintenance has to be available for review by the monitor.

5.1 Screening procedures

5.1.1 Informed consent procedure

This study is conducted in male subjects between 5 and 16 years of age. As for the informed consent procedure a distinction is made between subjects of twelve years of age and older and subjects under twelve years of age.

Subjects under 12 years of age

Both the subject and his parents (if they are the legal guardians) or legal guardian will receive oral and written information concerning the study. The subject and his parents/legal guardian should be given adequate time to read the information leaflet and an opportunity to ask the investigator any questions.

Following the provision of subject information, written consent will be obtained from the subject's parents or legal guardian prior to any study-specific procedure. The subject is not to enter the study if his parents/legal guardian have/has not understood the written and verbal information provided and/or has not personally signed and dated the consent form. A copy of the subject information leaflet and the signed informed consent will be provided to the subject's parents/legal guardian and a copy will be retained by the investigator.

Subjects between 12 and 18 years of age

Both the subject and his parents (if they are the legal guardians) or legal guardian will receive oral and written information concerning the study. The subject and his parents/legal guardian should be given adequate time to read the information leaflet and an opportunity to ask the investigator any questions.

Following the provision of subject information, written consent will be obtained from the subject and his parents or legal guardian prior to any study-specific (screening) procedure. The subject is not to enter the study if he or his parents/legal guardian have/has not understood the written and verbal information provided and/or if the subject or his parents/legal guardian has not personally signed and dated the consent form. A copy of the subject information leaflet and the signed informed consent will be provided to the subject and his parents/legal guardian and a copy will be retained by the investigator.

5.1.2 Screening of subjects

Patient selection will in first instance be based upon a search in a coded database, containing DNA diagnostic data of Duchenne patients. Patients will then be approached and asked for consent. Subsequently, they will be screened according to the inclusion and exclusion criteria. Screening will include evaluation of full DNA diagnostic report, an interview and the assessments as described in the schedule of assessment (Appendix 2: Table 4). Those who meet the selection criteria will be allocated a subject number (subject identification code) which, together with his initials, will ensure unambiguous identification throughout the study.

A skin biopsy will be taken during the screening period for isolation of fibroblasts. These fibroblasts will be converted into myogenic cells, which is a routine procedure in the Prosensa laboratory. For most patients the genetic diagnosis has been assessed on DNA level only, and RNA analysis has typically not been performed. To exclude unexpected effects on splicing and to confirm the mutation on RNA level, RNA will be isolated from the converted cells and analyzed by RT-PCR and sequencing. The cells will also be treated with study drug PRO051 to confirm that the anticipated exon 51 skipping can indeed be obtained.

An alternative and exploratory assay for this subject screening on molecular level will be applied in parallel. It is based on confirmation of patient's mutation and PRO051 response on RNA level using peripheral blood mononuclear cells isolated from small volume (4 ml) blood samples. Upon validation in this study, this relatively simple assay may significantly reduce the molecular screening procedure in future clinical studies and/or applications from 4-6 weeks to 1-2 weeks.

Subject identification codes and initials will be recorded on a subject identification code list, which will be kept on file at the study-site.

5.1.3 MRI

At screening, an MRI will be performed to assess the quality of the muscle in which the biopsy is planned. The MRI is to be performed within 6 weeks prior to the first study medication administration. Subjects will be positioned in the MRI with the legs first. This will avoid discomfort of the subject as this position allows the head of the subject to stay outside the MRI tunnel or at the border of the tunnel.

Abnormalities of the muscle will be assessed using T1-weighted MRI as described by Mercuri et al. and are quantified as mild, moderate or severe[22]. The following criteria apply:

- Mild – traces of increased signal intensity in an otherwise preserved muscle
- Moderate – less than 50% of the muscle show increased intensity
- Severe – at least 50% of the muscle shows increased intensity

5.1.4 Skin biopsy – *in vitro* dystrophin mRNA

If not previously already performed, a skin biopsy will be taken at screening to confirm the patient’s original DNA diagnosis on RNA level, being a mutation correctable by exon 51 skipping and allowing a positive response to PRO051. The skin biopsy procedure is to be performed at 6 weeks prior to the first study medication administration in order to allow sufficient time to perform the diagnosis on RNA level.

The skin punch biopsy is collected, stored and transported in sterile cell proliferation culture medium at ambient temperature. Transportation of the sample to the Prosensa Laboratory is within 24 hours by courier mail.

For analytical methods refer to Appendix 4.

5.1.5 Peripheral blood mononuclear cells (PB-MNC) – *in vitro* dystrophin mRNA (exploratory)

At the Prosensa laboratory, an alternative and exploratory assay has been developed which may be used as a straightforward and fast method for confirming patient’s original DNA diagnosis and positive response to PRO051 on RNA level. This assay applies peripheral blood mononuclear cells instead of differentiated muscle cell (myotube) cultures derived from a skin biopsy.

Whole blood samples (4 ml) will be collected into EDTA tubes at screening and should be stored at room temperature and sent at room temperature to Prosensa Laboratory within 24 hours by courier mail.

For analytical methods refer to Appendix 4.

5.1.6 Pre-study diagnostic muscle biopsy

If a diagnostic muscle biopsy has been performed at any time prior to the signing of the informed consent, the result for presence of dystrophin in fibers will be collected (in % of fibers positive for dystrophin).

5.2 Effect parameters during study period

5.2.1 mRNA production, dystrophin expression and PRO051 levels in muscle biopsy

A muscle biopsy taken from the tibialis anterior muscle will be taken according to a sparse sampling schedule in order to limit the number of biopsies taken per subject. In cohort 1, a muscle biopsy will be taken at visit 1 (screening) and at visit 8 (day 43). In the other cohorts, a muscle biopsy will be taken at visit 8 (day 43) and at visit 10 (day 78).

The biopsy instrument to be employed is a conchotome (a forceps with two sharp-edged jaws)[23]. This instrument is suitable for muscle biopsies in children and does not require full anesthesia. The child can drink or eat normally before the procedure.

At the biopsy site 1 g of a local anesthetic cream (EMLA cream, a hydrophilic cream containing 25 mg lidocaine and 25 mg prilocaine per g) will be applied and covered by a plastic plaster to enhance absorption. The cream will be applied one hour prior to the biopsy to assure good penetration of the skin. A sedative may also be given prior to the biopsy.

After removal of the cream the skin is locally anaesthetized by subcutaneous injection of 3-4 ml of lidocaine 1% without epinephrin. The skin above the anterior tibial muscle is cut parallel to the tibial bone over a length of approximately 1.5 cm. Next a similar incision is made in the muscle fascia. The closed bit of the forceps is introduced through these incisions. The bit is opened just below the muscle fascia to allow muscle tissue to enter the bit. The bit is closed thereby cutting a small piece of muscle of approximately 0.4 x 0.4 x 0.4 cm. The closed forceps is removed and the muscle biopsy is immediately frozen in liquid nitrogen-cooled 2-methylbutane and stored at -80°C till shipment.

After sufficient pieces of muscle tissue (2 to 3 pieces of 100 to 200 milligram each) have been collected, the wound is closed using three sterile adhesive strips. After five days the strips can be removed and replaced by a plaster.

5.2.1.1 mRNA production

Endogenous production of the expected mRNA will be assessed at the Prosensa laboratory in muscle tissue collected via the muscle biopsy.

For analytical methods refer to Appendix 4.

5.2.1.2 Dystrophin expression

Dystrophin expression will be assessed at the Prosensa laboratory in the muscle biopsies by immunofluorescence analyses of cross-sections and by western blot analyses of total protein extracts.

For analytical methods refer to Appendix 4.

5.2.1.3 PRO051 level

PRO051 levels in muscle tissue will be assessed at the Prosensa laboratory in the left-over material from the muscle biopsy after the results have been obtained for the mRNA production and dystrophin expression in the muscle tissue.

For analytical methods refer to Appendix 4.

5.2.2 mRNA production in peripheral blood mononuclear cells

Whole blood samples (4 ml) will be collected into EDTA tubes at visit 2, 3, 5, 7 and 8 to assess exon skipping in mononuclear blood cells. The blood samples should be stored at room temperature and sent at room temperature to Prosensa Laboratory within 24 hours by courier mail.

For analytical methods refer to Appendix 4.

5.2.3 Muscle function

5.2.3.1 Timed tests

The timed tests consist of 10-meter walk/run test, timed rising from floor and stair climb. This combination of tests will be performed at visit 1, visit 1a, visit 2, visit 3, visit 4, visit 5, visit 6, visit 7, visit 8, visit 10 and visit 12.

10-Meter walk/run test

The subject will be asked to traverse a marked 10-meter measured walkway as quickly as he safely can. The 10-meter walk/run test should be performed preferably barefoot without shoes or orthoses. If not possible, testing can be done with shoes/orthoses and any use of shoes/orthoses should be documented. Time is recorded with stop watch from when his first foot crosses the start line till when the second foot crosses the finish line. If wall is touched, it should be noted how often. Care needs to be taken to ensure that the patient is safe when completing this test. The assessor can walk nearby to provide ‘emergency’ help should it be needed, but must not support or provide manual assistance for the patient in any way.

Timed rising from floor

The subject will be told to stand up as quickly as possible from supine position with his arms by his side. The patient is allowed to use his arms for support while rising from the floor. Time is recorded with a stopwatch from the initiation of movement until the assumption of upright standing. The area should be free from furniture and the patient should not be wearing orthoses or using any aids.

Stair climb

The subject will need to ascend four steps. Time is recorded with a stopwatch from the initiation of movement until the subject stands on the fourth step. If a flight of steps with handrail is available these should be used. If not, a box step (approximately 15cm high) should be used. A plinth or other immovable object may need to be available to provide support.

5.2.4 6-Minute walk test

Subjects will be requested to walk for 6-minutes at visit 1, visit 8 and visit 12. The subject will be asked to walk at his own preferred speed up and down the fixed distance of 25 meters until they are told to stop after 6 minutes. The test should be performed preferably barefoot and without aid. If not possible, testing can be done with shoes/orthoses/aid and any use of shoes/orthoses/aid should be documented. The subjects are warned of the time and are told that they may stop earlier if they feel unable to continue. The total distance walked within 6 minutes (or until the subjects stopped in case of early termination of the test) will be collected in meters.

5.2.5 Muscle strength

5.2.5.1 Quantitative Muscle Testing (QMT)

Quantitative muscle testing including spirometry will be performed visit 1, visit 1a, visit 2, visit 3, visit 4, visit 5, visit 6, visit 7, visit 8, visit 10 and visit 12, using the CINRG Quantitative Measuring System (CQMS)[24,25].

5.2.5.2 Manual Muscle Testing (MMT)

Manual muscle testing will be assessed at visit 1, visit 8 and visit 12. The Medical Research Council Scale is composed of a rating of 0-5 assigned to each muscle group tested. The Medical Research Council Scale has been modified and formalized to account for more grades of muscle weakness using a plus or minus designation (see Table 3).

Table 3. Manual Muscle testing using a modified Medical Research Council Scale.

Grade	*Description*
5	Normal strength
5-	Barely detectable weakness
4+	Muscle is weak, but moves the joint against a combination of gravity and mod-to-max resistance
4	Muscle is weak, but moves the joint against a combination of gravity and moderate resistance
4-	Muscle is weak, but moves the joint against a combination of gravity and minimal resistance
3+	Joint is moved against gravity and a small amount of resistance. Muscle is capable of transient resistance but collapses abruptly. Not to be used for muscles capable of sustained resistance throughout the whole range of movement.
3	Joint is moved through the full available range of motion against gravity but cannot accept resistance.
3-	Joint is moved against gravity but not through the full available range of motion.
2	Joint is moved when the affects of gravity are minimized with a position change.
1	A flicker of activity is seen or palpated in the muscle.
0	No palpable muscle activity.

5.3 Safety parameters during study period

5.3.1 Physical examination

A full physical examination will be carried out at visit 1, visit 2, visit 3, visit 4, visit 5, visit 6, visit 8, visit 10 and visit 12. The following body systems will be examined: general appearance; ear, nose, throat; cardiovascular; pulmonary; gastrointestinal; urogenital; nervous system; musculoskeletal; extremities; other. In addition, weight, height and Body Mass Index will be assessed at visit 1, visit 8 (day 43) and visit 12 (day 120).

5.3.2 Vital signs

At visit 1, visit 2, visit 3, visit 4, visit 5, visit 6, visit 7, visit 8, visit 10 and visit 12, systolic and diastolic blood pressure, pulse rate and respiratory rate will be recorded after five minutes rest in semi-recumbent position. At visit 2, visit 3, visit 4, visit 5 and visit 6 the measurements will be done at the time points indicated in Table 5 (Appendix 2).

Measures of blood pressure will be made using an automatic device. The same equipment will be used throughout the trial. Measurements will always be performed at the same arm.

5.3.3 ECG

A 12-lead ECG will be made at visit 1, visit 2 (at time points as indicated in Table 5 in Appendix 2), visit 6 (at time points as indicated in Table 5 in Appendix 2) and visit 12.

The following parameters will be assessed: heart rate, PR-interval, QRS-interval, QT-interval, QT-c interval (Bazett). In addition, an assessment of abnormal morphology (rhythm, axis, P-wave, QRS complex, ST-T segment, T-wave) will be made.

5.3.4 Safety laboratory (routine biochemistry and hematology, coagulation, urinalysis, complement activation, inflammatory and immune response)

5.3.4.1 Routine biochemistry and hematology

Routine biochemistry and hematology will be performed at visit 1, visit 1a, visit 2 prior to the first injection, visit 3, visit 4, visit 5, visit 6, visit 7, visit 9 and visit 12.

The parameters to be assessed for routine biochemistry and hematology are described in Appendix 3. There is no food restriction prior to blood sampling. The samples will be collected and processed according to standard hospital procedures and assessed by the local laboratory of the participating sites.

Special attention is required for the liver related enzymes in the clinical biochemistry assessments. A number of these liver enzymes (ASAT, ALAT and LDH) are also present in muscle and hence elevated levels are found in plasma (or serum) upon muscle damage, as in DMD patients. Indeed, these ASAT and ALAT findings in DMD patients have been found to correlate quantitatively with muscle specific CK levels in plasma (or serum) in DMD patients. Therefore, changes in ASAT and ALAT should be monitored in relation to CK for the evaluation of hepatotoxicity. If the changes in ASAT and ALAT levels (of over 3xULN) is disproportional to the CK changes, further evaluation is required. Dependent on the findings, this can include (i) further data analysis (e.g. graphical evaluation of trends in ALAT, ASAT and CK over time and compared to pre-treatment levels and/or regression analysis between ALAT/ASAT and CK levels for all samples of the patient), (ii) taking an additional confirmatory sample, (iii) starting more frequent monitoring and/or (iv) evaluating other alternative explanations. The sponsor should be notified of such concern. If deemed necessary, drug administration will be discontinued.

Other measures of hepatic and biliary function, including bilirubin, γGT and ALP are not affected by the disease. However, ALP may be influenced by age (ALP from bone).

5.3.4.2 Coagulation

A blood sample for determining the aPTT will be collected at visit 2 and visit 6 at time points as described in Table 5 in Appendix 2. The samples will be collected and processed according to standard hospital procedures and assessed by the local laboratory of the participating sites.

5.3.4.3 Urinalysis

A midstream sample of urine is collected at visit 1, visit 1a, visit 2, visit 3, visit 4, visit 5, visit 6, visit 7, visit 9 and visit 12 in order to assess the urinalysis parameters (dipstick and quantitative) as described in Appendix 3.

The urine samples will be collected and processed according to standard hospital procedures and assessed by the local laboratory of the participating sites.

A 4 ml aliquot of all urine samples will be stored at ≤-60°C in case future analysis is needed of other urinary markers of renal function in DMD patients.

5.3.4.4 Complement activation

Determination of complement activation by means of measuring complement split factors C3a, SC5b-9 and Bb will be performed at visit 2 and visit 6 at time points as described in Table 5 in Appendix 2. The collection, processing and shipping of these samples to the central laboratory will be described in the study specific laboratory manual.

5.3.4.5 Inflammatory response

Inflammatory response will be determined by measuring IL-6, TNF-α and MCP-1 at visit 2 and visit 6 at time points as described in Table 5 in Appendix 2. The collection, processing

and shipping of these samples to the central laboratory will be described in the study specific laboratory manual.

5.3.4.6 Dystrophin antibodies in blood

As DMD patients lack the dystrophin protein, it cannot entirely be excluded that a marked production of this protein by DMD patients may cause an immune response. It is not considered likely because spontaneous (natural) exon skipping occurs in DMD patients to some extent. This results in so-called revertant fibers expressing the (shortened) dystrophin protein. The frequency of such revertant fibers is generally far less than 1-2%, but nevertheless this could have been sufficient for immunological tolerance. This notion is supported by the absence of circulating antibodies to dystrophin in a study in *mdx* mice after induction of exon skipping and dystrophin protein expression by systemic application of oligoribonucleotides[13].

As an autoimmune response needs to develop over time and it is likely that antibodies will remain detectable for months, blood samples are only taken before administration, at the expected dystrophin peak levels and at the end of study. The assay is able to detect potential IgM and IgG antibodies against dystrophin. In case of a positive response, confirmation of specificity will be obtained by employing existing competing dystrophin antibodies.

For analytical methods refer to Appendix 4.

Formation of antibodies to dystrophin in blood will be determined at visit 2, visit 8 and visit 12. The collection, processing and shipping of these blood samples to the Prosensa laboratory will be described in the study specific laboratory manual.

5.3.5 Adverse events

The investigator will determine whether any AEs have occurred between visits. The subjects will be questioned using non leading questions and no specific symptoms will be suggested. Refer to Section 7 for further information on adverse event reporting.

5.3.6 Local tolerability

Local tolerability at the injection site(s) will be established by assessing the absence or presence and severity of erythema, edema, pain, increased temperature, induration and bruising on a scale of 0 to 3 where:

0= absent
1= mild
2= moderate
3= severe

5.3.7 Concomitant medication

The investigator should record the use of all concomitant medications, both prescribed and over-the-counter, on the CRF. This includes drugs used on a chronic and as-needed basis. The following will be recorded on the CRF: name of medication, reason for taking concomitant medication, dose, unit, regimen, route of administration, start date and end date (if applicable).

5.4 Pharmacokinetic parameters during study period

Phosphorothioate oligoribonucleotides distribute fast to tissues but remain stable with an expected terminal half life of approximately 4 weeks. This results in low plasma levels indicative of tissue redistribution at steady state. A sensitive ELISA has been set up that is able to measure such low plasma concentrations. This will allow the determination of trough plasma values and to determine overall tissue elimination rate for further evaluation of dose response relations.

Pharmacokinetic sampling will be done at all visits except visit 1 and visit 1a. Sampling has to be done prior to the administration of PRO051, and for visit 2 and visit 6 at time points as indicated in Table 5 (Appendix 2).

Per sample a volume of 0.6 ml venous blood will be collected to obtain at least 0.2 ml plasma. Samples will be taken from an intravenous cannula during the hospitalization visits and via venous puncture at the other visits.

Whole blood samples (0.6 ml) will be collected into EDTA tubes. After collection the samples should be centrifuged for 10 minutes at 1500G at 4°C. Plasma will be stored in screw cap polypropylene tubes and kept deeply frozen at –20°C until shipment.

At the Prosensa laboratory, PRO051 levels will be measured in plasma using GLP validated assay. For the analytical methods refer to Appendix 4.

5.5 Effect parameters during treatment beyond study period

5.5.1 mRNA production and dystrophin expression in muscle biopsy

A muscle biopsy taken from the tibialis anterior muscle will be taken at visit 37 and an optional muscle biopsy will be taken at visit 65 (week 52) or as soon as this protocol is implemented (Amendment 6) and convenient to the investigative site and subject. The optional muscle biopsy will be performed to track the effectiveness of PRO051 administration over a 12 month period. The muscle biopsy samples will be collected by open biopsy or with the conchotome method according to standard hospital procedures for obtaining muscle biopsies from children. The minimum amount of muscle tissue required is a small piece of muscle of AT LEAST 0.5 x 0.5 x 0.5 cm. The muscle tissue is immediately frozen in liquid nitrogen-cooled 2-methylbutane and stored at -80°C or -70ºC till shipment. The collection, processing and shipment of these muscle biopsy samples to the Prosensa laboratory will be described in detail in the study specific laboratory manual.

The child can drink or eat normally before the procedure.

5.5.1.1 mRNA production

Endogenous production of the expected mRNA will be assessed at the Prosensa laboratory in muscle tissue collected via the muscle biopsy.

Small samples of the muscle biopsy will be stored for retrospective analysis. These will only be analysed in the event that any subject does not respond, to determine the reasons why. No additional material will be taken from the subjects.

Total muscle RNA will be isolated from muscle tissue sections and will be analyzed by RT-PCR [21].

RT-PCR analysis focusing on the area flanking the targeted exon 51 will be performed to detect specific exon 51 skipping in muscle. Depending on the subject’s mutation different sets of DMD-gene specific RT and PCR primers will be used. Sequence analysis will be performed on isolated PCR products to confirm specific exon 51 skipping.

For analytical methods refer to Appendix 4.

5.5.1.2 Dystrophin expression

Dystrophin expression will be assessed at the Prosensa laboratory in the muscle biopsies by immunofluorescence analyses of cross-sections and by western blot analyses of total protein extracts.

For analytical methods refer to Appendix 4.

5.5.1.3 Storage of muscle biopsy samples

Muscle biopsy samples will be stored appropriately for potential scientific investigations in the future, with appropriate informed consent by the subjects and /or their parents/guardians. All

subjects have the right to change their mind regarding the use of stored samples for research at any time by notifying their doctor.

5.5.2 Muscle function

5.5.2.1 Timed tests

The timed tests consist of 10-meter walk/run test, timed rising from floor and stair climb. This combination of tests will be performed at selected visits. Refer to Tables 6 - 14 in Appendix 2 for exact time points, and refer to section 5.2.3.1 for execution instructions.

10-Meter walk/run test

The subject will be asked to traverse a marked 10-meter measured walkway as quickly as he safely can. The 10-meter walk/run test should be performed preferably barefoot without shoes or orthoses. If not possible, testing can be done with shoes/orthoses and any use of shoes/orthoses should be documented. Time is recorded with stop watch from when his first foot crosses the start line till when the second foot crosses the finish line. If wall is touched, it should be noted how often. Care needs to be taken to ensure that the patient is safe when completing this test. The assessor can walk nearby to provide ‘emergency’ help should it be needed, but must not support or provide manual assistance for the patient in any way.

Timed rising from floor

The subject will be told to stand up as quickly as possible from supine position with his arms by his side. The patient is allowed to use his arms for support while rising from the floor. Time is recorded with a stopwatch from the initiation of movement until the assumption of upright standing. The area should be free from furniture and the patient should not be wearing orthoses or using any aids.

Stair climb

The subject will need to ascend four steps. Time is recorded with a stopwatch from the initiation of movement until the subject stands on the fourth step. If a flight of steps with handrail is available these should be used. If not, a box step (approximately 15cm high) should be used. A plinth or other immovable object may need to be available to provide support.

5.5.3 6-Minute walk test

Subjects will be requested to walk for 6-minutes at selected visits, refer to Tables 6 - 14 in Appendix 2 for exact time points. The subject will be asked to walk at his own preferred speed up and down the fixed distance of 25 meters until they are told to stop after 6 minutes. The test should be performed preferably barefoot and without aid. If not possible, testing can be done with shoes/orthoses/aid and any use of shoes/orthoses/aid should be documented. The subjects are warned of the time and are told that they may stop earlier if they feel unable to continue. The total distance walked within 6 minutes (or until the subjects stopped in case of early termination of the test) will be collected in meters. In addition, the distance walked at 1, 2, 3, 4 and 5 minutes will be collected in meters. Also, the time needed to walk the distance of 25 meters is recorded for every 25 meter.

5.5.4 Muscle strength

5.5.4.1 Handheld myometry

The strength of the knee flexors, knee extensors, elbow flexors, elbow extensors, shoulder abductors and wrist dorsiflexors will be assessed at selected visits (Tables 6 - 14 in Appendix 2 indicate the exact time points), using a microFET dynamometer (Biometrics BV, Almere, the Netherlands).

5.5.4.2 Spirometry

Spirometry will be done at selected visits, refer to Tables 6 - 14 in Appendix 2 for the exact time points. The following parameters will be assessed: FVC, FEV_1, mouth pressure inhale, mouth pressure exhale, peak flow, peak cough flow.

5.5.4.3 Parent Questionnaire

With the implementation of protocol Amendment 6, a short questionnaire will be completed by the subject's parents or legal guardian once every 6 months to capture the following:

- loss of any skills or daily activities
- improvements in daily activities
- development of new skills.

5.6 Safety parameters during treatment beyond study period

5.6.1 Physical examination

A full physical examination, weight, height and Body Mass Index will be assessed at selected visits, refer to Tables 6 - 14 in Appendix 2 for the exact time points. The following body systems will be examined: general appearance; ear, nose, throat; cardiovascular; pulmonary; gastrointestinal; urogenital; nervous system; musculoskeletal; extremities; dermatology; other.

Particular attention will be paid to the local tolerability at the site of the subcutaneous injections. Major events notable for disease progression that do not constitute an AE should also be noted on physical examination.

5.6.2 Vital signs

At selected visits prior to study medication administration and at t=3hr after study medication administration (refer to Tables 6 - 14 in Appendix 2 for the exact time points), systolic and diastolic blood pressure, pulse rate and respiratory rate will be recorded after five minutes rest in semi-recumbent position.

Measures of blood pressure will be made using an automatic device. The same equipment will be used throughout the trial. Measurements will always be performed at the same arm.

5.6.3 ECG

A 12-lead ECG will be made at selected visits, refer to Tables 6 - 14 in Appendix 2 for the exact time points.

The following parameters will be assessed: heart rate, PR-interval, QRS-interval, QT-interval, QT-c interval (Bazett). In addition, an assessment of abnormal morphology (rhythm, axis, P-wave, QRS complex, ST-T segment, T-wave) will be made.

5.6.4 Echocardiography

An echocardiography will be made at selected visits, refer to Tables 6 - 14 in Appendix 2 for the exact time points.

The following parameters will be derived from M-mode measurements: left ventricular ejection fraction, fractional shortening, left ventricular posterior wall thickness, left ventricular septum wall thickness, left ventricular end-systolic diameter, left ventricular end-diastolic diameter, and left ventricular ejection fraction modified Simpson's.

5.6.5 Safety laboratory

5.6.5.1 Routine biochemistry and hematology

Routine biochemistry and hematology will be performed at selected visits, refer to Tables 6 - 14 in Appendix 2 for the exact time points.

The parameters to be assessed for routine biochemistry and hematology are described in Appendix 3. There is no food restriction prior to blood sampling. The samples will be collected and processed according to standard hospital procedures and assessed by the local laboratory of the participating sites.

Non-clinical studies with PRO051 suggest that it potentially may have hepatotoxic and nephrotoxic properties as it accumulates in the liver and kidney and is also associated with thrombocytopenia.

Therefore, monitoring of hepatic and renal function as well as thrombocyte count is of paramount importance. However, standard markers of hepatic and renal function may be confounded by the effects of DMD e.g. DMD subjects may have elevated ALAT, ASAT and CK levels (common markers of hepatic function) due to ‘leaky’ sarcolemmal membranes around muscle fibers, and serum and urinary creatinine concentrations (markers of renal function) may be variable due to changes in muscle mass. With this information in mind, the following parameters will be used for monitoring hepatic and renal function:

Hepatic function: ALAT, ASAT, ALP, total bilirubin, bilirubin fractions (direct bilirubin), γ-GT, GLDH and LDH.

Renal function: Urinalysis (including microscopy), serum and urine cystatin C, KIM-1, alpha-1-microglobulin and serum creatinine.

Urinalysis will be conducted on the first morning specimen after 8 hours of not drinking.

5.6.5.2 Thrombocyte Count

Thrombocyte count will be performed on a two weekly basis during the treatment part of each 12 week cycle, and the following monitoring and discontinuation criteria apply:

- If thrombocytes are below the lower level of the normal range, thrombocytes will be checked prior to the next scheduled dose of study medication. Study medication will only be administered if thrombocytes are >75 x 10^9/L;
- If thrombocytes are still <75 x 10^9/L, they will be checked on alternate days until they reach >100 x 10^9/L;
- If thrombocytes are <75 x 10^9/L, study medication will be stopped and will only be considered for re-challenge should a clear alternative (non-drug related) cause be found after discussion between the Sponsor and Investigator;
- If the thrombocyte count has fallen >25% from the previous count and is <100 x 10^9/L then study medication should not be administered until thrombocytes return to >100 x 10^9/L;
- Investigators should be alert to trends for a consistent decrease in thrombocytes over two or more visits that may not meet the above criteria.

5.6.5.3 Complement activation

Determination of complement activation (usage) by measuring the pool for complement factor C3 will be performed at selected visits; refer to Tables 6 - 14 in Appendix 2 for the exact time points. The samples will be collected and processed according to standard hospital procedures and assessed by the local laboratory of the participating sites.

5.6.5.4 Troponin

The requirement for measuring troponin was removed with the implementation of this protocol amendment (Amendment 6).

5.6.5.5 Inflammatory response

Determination of inflammatory response by measuring C-reactive protein (CRP), fibrinogen, haptoglobin, MCP-1 and the albumin/globulin ratio will be performed at selected visits; refer to Tables 6 - 14 in Appendix 2 for the exact time points. The samples will be collected and processed according to standard hospital procedures and assessed by either the local laboratory of the participating sites or by the central laboratory (MCP-1).

Monitoring and stopping criteria for inflammation;

CRP >2 x ULN and 2 x reference value taken at Visit 29 (first time CRP was measured in Extension phase of PRO051-02), (along with symptoms/signs of inflammation in the absence of any other likely causative factors)

If criterion is met, test should be repeated immediately. If the result still meets the criterion, the study drug should be discontinued until further investigations are performed to establish the cause.

OR

• MCP-1 >2 x reference value taken at Visit 85 (first time MCP-1 was measured in Extension phase of PRO051-02) and Complement C3 < LLN (along with symptoms/signs of inflammation in the absence of any other likely causative factors.)

If criteria are met, tests should be repeated immediately. If the results still meet the criteria, the study drug should be discontinued until further investigations are performed to establish the cause.

OR

• CRP, MCP-1, or total C3 are persistently (i.e., on more than 2 consecutive assessments) beyond the threshold levels described above in the absence of inflammation signs/symptoms, or are associated with elevations in fibrinogen or haptoglobin > 2 x ULN

If the investigator believes and/or investigations show other likely causative factors (e.g., acute influenza, acute trauma) are responsible; and/or abnormal laboratory results along with signs/symptoms of inflammation have resolved, these must be discussed with and agreed by the Medical Monitor prior to re-starting study drug. In situations where no alternative cause is established and abnormal laboratory results with the presence of inflammatory signs and symptoms persist, study drug should be permanently discontinued.

5.6.5.6 Dystrophin antibodies in blood

Formation of antibodies to dystrophin in blood will be determined at selected visits, refer to Tables 6 - 14 in Appendix 2 for the exact time points. The collection, processing and shipping of these blood samples to the Prosensa laboratory will be described in the study specific laboratory manual.

For analytical methods refer to Appendix 4.

5.6.5.7 Coagulation

A blood sample for determining the aPTT will be collected at selected visits, refer to Tables 6 - 14 in Appendix 2 for the exact time points. The samples will be collected and processed

according to standard hospital procedures and assessed by the local laboratory of the participating sites. Any subject reaching a threshold value of aPTT of ≥1.5x ULN should be immediately withdrawn.

5.6.5.8 Cystatin C

A blood sample for determining cystatin C will be collected at selected visits, refer to Tables 6 - 14 in Appendix 2 for the exact time points. The samples will be collected and processed according to standard hospital procedures and assessed by the local laboratory of the participating sites. In case the assessment is not available at the local laboratory of a participating site, the samples will be analyzed at an external laboratory. The collection, processing and shipping of these blood samples to the external laboratory will be described in the study specific laboratory manual.

5.6.5.9 Urinalysis

A first morning urine specimen is collected at selected visits (refer to Tables 6 - 14 in Appendix 2 for the exact time points) in order to assess the urinalysis parameters as described in Appendix 3.

At visit 13, 25, 37, 49, 61 and 4 weekly thereafter an additional first morning sample of urine is collected for alpha-1-microglobulin, KIM-1 and cystatin C assessment.

- If during treatment any of the following occur, study drug must be stopped and a 24-hour urinalysis performed as soon as possible but at the latest by the next scheduled visit: urinary protein concentration ≥0.2 g/L and <0.45g/L on two consecutive samples;
    - NOTE: if urinary protein concentration is ≥0.45g/L on urinalysis, stop study drug and repeat protein analysis. If the repeat urinary protein value is ≥0.2g/L, continue to withhold study drug and perform a 24 hour urinalysis. If the repeat urinary protein is <0.2g/L, study drug may be restarted.
- protein/creatinine ratio in the morning sample is >0.5 on two consecutive samples;
- serum concentration of cystatin C is above the normal range and 50% above the baseline value.

24-hour urinalysis to include creatinine concentrations for creatinine clearance assessment, total protein and protein electrophoresis.

If the results of the 24-hour urinalysis triggered by any of the above do not meet criteria defined in section 5.6.8.2, study drug may be restarted.

The urine samples will be collected and processed according to standard hospital procedures and assessed by the local laboratory of the participating sites.

5.6.5.10 Disseminated Intravascular Coagulation

If either aPTT ≥1.5 x ULN, or thrombocytes <75 x 10^9/L or fibrinogen <50% of LLN are reached, the tests for fibrin split products and D-dimer should be performed. If fibrin split products and/or D-dimers are >ULN, and the aPTT or thrombocytes or fibrinogen remain outside of protocol limits as described above, study drug must be stopped and the subject should be immediately withdrawn from the study if not already discontinued. An elevated d-dimer or fibrin split products in the absence of aPTT >1.5x ULN, or thrombocytes <75 x 10^9/L or fibrinogen <50% of LLN does not require stopping study drug unless it is associated with clinical signs or symptoms (e.g. bleeding).

5.6.6 Adverse events

The investigator will determine whether any AEs have occurred between visits. The subjects will be questioned using non leading questions and no specific symptoms will be suggested. Refer to section 7 for further information on adverse event reporting.

5.6.7 Concomitant medication

The investigator should record the use of all concomitant medications, both prescribed and over-the-counter, on the CRF. This includes drugs used on a chronic and as-needed basis. The following will be recorded on the CRF: name of medication, reason for taking concomitant medication, dose, unit, regimen, route of administration, start date and end date (if applicable).

5.6.8 Laboratory Safety Parameter Stopping and Follow-up Criteria

5.6.8.1 Hepatic Toxicity

Laboratory safety assessments including liver chemistries will be performed at selected visits, refer to Tables 6 - 14 in Appendix 2 for exact time points.

- ALAT ≥8x ULN and total bilirubin 2x ULN (plus >35% ‘direct’ bilirubin) or ALAT ≥8x ULN and INR >1.5.
- γ-GT >2x ULN and bilirubin 2x ULN (plus >35% ‘direct’ bilirubin) or γ-GT >2x ULN and INR>1.5

  If a subject’s results meet either of these criteria, the tests should be repeated immediately. If the results still meet these criteria, the study medication should be stopped until such time that the Sponsor (in discussion with the Investigator) considers that the benefit of further treatment with PRO051 outweighs the risk to the individual subject.

  NOTE: if serum bilirubin fractionation is not immediately available, study medication should be discontinued if ALAT ≥8x ULN and total bilirubin ≥2x ULN. Serum bilirubin fractionation should be performed if testing is available. If testing is unavailable, record presence of detectable urinary bilirubin on dipstick, indicating direct bilirubin elevations and suggesting liver injury.
- ALAT ≥8x ULN associated with symptoms of hepatitis (e.g. onset or worsening of nausea, anorexia, jaundice or abdominal pain) or hypersensitivity (fever, rash, eosinophilia).

  If a subject’s results meet this criterion, the test should be repeated immediately alongside a discussion with the Medical Monitor. Depending on the result of the re-test and general clinical picture, appropriate action with regards to discontinuing study medication will be decided between the Sponsor and Investigator.

Given the progressive and fatal nature of this disease, it is felt important that the hepatic stopping criteria allow the flexibility for the potential to re-start dosing in this subject population following an individual subject benefit: risk assessment.

However, if a subject’s results meet the criterion below, the tests should be repeated immediately. If the results still meet this criterion, the study drug should be permanently stopped.

ALT >2x baseline values and bilirubin 2x ULN (plus >35% ‘direct’ bilirubin) or ALT >2x baseline values and INR >1.5.

Standard liver follow-up will be implemented following repeat testing confirmation of any of liver findings as described above. If a subject’s results meet any of these criteria the following processes should be completed:

- Report the event to Prosensa within 24 hours of learning of its occurrence;

- Complete the appropriate case report form (CRF) and SAE data collection tool. Note:all events mentioned above must be reported as an SAE.;
- Complete the liver imaging and/or liver biopsy CRFs if these tests are performed;
- Make every reasonable attempt to have subjects return to clinic within 24 hours for repeat liver chemistries, liver event follow up assessments (see below), and close monitoring;
- A specialist or hepatology consultation is recommended;
- Monitor subjects twice weekly until liver chemistries (ALAT, ASAT, alkaline phosphatase, bilirubin) resolve, stabilize or return to within baseline values.

In addition, make every attempt to carry out the liver event follow up assessments described below:

- Viral hepatitis serology including:
  - Hepatitis A immunoglobulin M (IgM) antibody;
  - Hepatitis B surface antigen and Hepatitis B Core Antibody (IgM);
  - Hepatitis C RNA;
  - Cytomegalovirus IgM antibody;
  - Epstein-Barr viral capsid antigen IgM antibody (or if unavailable, obtain heterophile antibody or monospot testing);
  - Hepatitis E IgM antibody;
- Serum creatine kinase (CK), glutamate dehydrogenase (GLDH) and lactate dehydrogenase (LDH);
- Fractionate bilirubin, if total bilirubin ≥2x ULN;
- Obtain complete blood count with differential to assess eosinophilia;
- Collect blood sample for measurement of drug concentration within one month of last dose. Record the date/time of this blood sample draw and the date/time of the last dose of study medication prior to blood sample draw on the CRF. If the date/time of the last dose is unclear, provide the subject’s best approximation. If the date/time of the last dose cannot be approximated OR a PK sample cannot be collected in the time period indicated above, do not obtain a PK sample. Instructions for sample handling and shipping are provided in the SPM;
- Record the appearance or worsening of clinical symptoms of hepatitis or hypersensitivity, such as fatigue, nausea, vomiting, right upper quadrant pain or tenderness, fever rash or eosinophilia as relevant on the AE report form;
- Record use of concomitant medications, acetaminophen (=paracetamol), herbal remedies, other over-the-counter medications, or putative hepatotoxins, on the concomitant medications report form;
- Record alcohol use on the liver event alcohol intake CRF.

The following are required for subjects with ALAT ≥8x ULN and total bilirubin ≥2x ULN (>35% direct) but are optional for other abnormal liver chemistries:

- Anti-nuclear antibody, anti-smooth muscle antibody, and Type 1 anti-liver kidney microsomal antibodies;
- Liver imaging (ultrasound, magnetic resonance, or computerized tomography) to evaluate liver disease.

5.6.8.2 Renal Toxicity

Urinalysis (as described in Section 5.6.5.9) will be conducted every 2 weeks during the treatment part of each 12 week cycle. In addition, 24-hour urinalysis will be conducted if any of the criteria described in Section 5.6.5.9 are met on two consecutive weekly samples.

If a subject meets any of the following criteria, study medication will be stopped until such time that the Sponsor (in discussions with the Investigator) considers that the benefit of further treatment with PRO051 outweighs the risk to the individual subject. Given the progressive and fatal nature of this disease, it is important that the renal stopping criteria allow the flexibility for the potential to re-start dosing in this population following an individual subject benefit: risk assessment.

- Proteinuria in 24-hour urine sample is either >300 mg/day or 8 $mg/m^2/h$ (whichever is reached first); or
- Albumin excretion in 24-hour urine sample is >50 mg/day; or
- New appearance of casts (other than hyaline casts up to 200/mL) or erythrocytes (≥5 cells/hpf). An additional fresh spot urine sample will be required for this analysis

Routine non-invasive renal imaging will not be performed for subjects participating in the study. However, if a subject experiences persistent and progressive renal impairment, further investigations which may include non-invasive imaging and renal biopsy will be considered on an individual subject basis.

An aliquot of urine for storage will be collected for potential future analysis for any subject that meets renal stopping and follow-up criteria at any time during the study. Additionally, at Visit 85 (week 68), Visit 117 (week 104) and yearly thereafter or early withdrawal, an aliquot of urine will be obtained and frozen for each subject to allow for storage and potential future analysis of emerging renal biomarkers

5.7 Pharmacokinetic parameters during treatment beyond study period

Pharmacokinetic sampling prior to study medication administration and at t = 3 hr (± 15 min) after study medication administration will be done at visit 13, 17, 21, 25, 29 and 37. Pharmacokinetic sampling prior to study medication administration will be taken at Visit 85, 89, 93, 97, 101, 105 and 109.

Pharmacokinetic sampling prior to study medication administration and at t = 0.5 hr (± 5 min), 2 hr (± 15 min), 3 hr (± 15 min), 4 hr (± 15 min), 6 hr (± 15 min), 9 hr (± 30 min), 12 hr (± 30 min) and 24 hr (± 3 hrs) after study medication administration will be done at visit 33. Subjects will be admitted to the hospital for an overnight stay at visit 33.

A pharmacokinetic sample will be taken if a subject has to have an interruption to drug administration. PRO051 levels will be measured and the results databased.

Per sample a volume of 2 ml venous blood will be collected to obtain plasma. Samples will be taken from an intravenous cannula during the hospitalization visit and via venous puncture at the other visits.

At Eurofins, PRO051 levels will be measured in plasma using GLP validated assay. For the analytical methods refer to Appendix 4.

5.8 Compliance with the protocol

Any deviation from the protocol must be explained and the reason entered on the CRF. Any subject who is entered into the study but is found not to meet the protocol entry criteria will be considered for withdrawal as a protocol violator after consultation with the sponsor.

5.9 Termination of the study

If the clinical observations in the study suggest that it may be unwise to continue, the sponsor may terminate the study. Additionally, the sponsor may terminate the trial at any time for safety or administrative reasons.

Should the trial be terminated prematurely, a written statement fully documenting the reasons for any termination will be provided to the IEC/IRB by the investigator.

5.10 DEXA Scan

A DEXA scan will be performed at visit 65 or as soon as this protocol is implemented (Amendment 6) and convenient to the investigative site and subject. A DEXA scan will be scheduled yearly thereafter. However, a DEXA scan may also be performed at any visit if deemed clinically necessary by the Investigator (i.e. rapid weight gain).

A DEXA total-body scanner will be used to obtain regional and whole body composition measurements using a three-compartment model of body composition: lean tissue mass (LTM), fat tissue mass (FTM), and bone mineral content (BMC). LTM, FTM and BMC will be determined using software algorithms based on derived regression equations. Percentage of body fat by DEXA for total body will be calculated using the formula: 100•FTM/ (FTM + LTM + BMC). In addition to body composition, area (in square centimeters) and bone mineral density (in grams per square centimeter) will be measured for each anatomical region. For regional body composition measurements, subdivision of the whole body data will be made into trunk, entire arm, upper arm, entire leg, thigh, and lower leg.

6 Study medication

6.1 Study agents

6.1.1 During treatment period

Product: 2'-O-methyl-phosphorothioate antisense oligoribonucleotide

Formulation: 5' UCA AGG AAG AUG GCA UUU CU 3'

Strength: 00 mg/ml

Vial content: 0.5 ml glass vial containing 50 mg

6.1.2 During treatment beyond period

Product: 2'-O-methyl-phosphorothioate antisense oligoribonucleotide

Formulation: 5' UCA AGG AAG AUG GCA UUU CU 3'

Strength: 200 mg/ml

Vial content: 1 ml glass vial containing 200 mg

6.2 Study medication manufacture, packaging, labeling and storage

The study medication GSK2402968 (formerly 2'O-methyl phosphorothioate oligonucleotide PRO051) is manufactured by GlaxoSmithKline, UK and prepared as a solution for injection. Manufacturing operations are performed in accordance with current Good Manufacturing Practice.

All medication used in this study will be prepared and labeled according to the rules of Good Manufacturing Practice, ICH-GCP and local regulatory requirements.

The medication should be stored in a dry and dark place at a temperature between 2 and 8°C.

6.3 Study medication administration

6.3.1 During treatment period

The study medication will be delivered as solution for subcutaneous injection in vials containing 0.5 ml with 100 mg/ml PRO051. Medication will be injected by qualified site personnel in the abdominal subcutis with at least 4 cm between injection places in case two or more injections are necessary. In case the calculated volume to be injected is less than 1 ml, a final volume of 1 ml should be prepared by adding saline.

6.3.2 During treatment beyond period

The study medication will be delivered as solution for subcutaneous injection in vials containing 1 ml of 200 mg/ml of GSK2402968 (formerly PRO051). Before drawing the solution, the vials need to be at ambient temperature and subsequently shaken manually for at least 10 seconds. Medication will be injected by qualified personnel in the abdominal subcutis. As there is the potential for injection site reactions with repeated study drug administration, it is strongly recommended to try and minimize any skin reactions by rotation of the injection site. Detailed instructions for the rotation of injection sites are provided in the Investigator Manual. The drug product should be administered within 4 hours after preparation. If the Investigator is confident that there are no medical concerns, subjects may receive some injections at home administered by a qualified person approved by the Investigator. All subjects must continue to attend the investigative site at least once a month

for each full assessment visit and all efficacy assessments must be done in the clinic. If the qualified person administering the injections has any concerns on subject wellbeing, the Investigator should be contacted by the qualified person to discuss and the subject may need to attend the clinic for the following injection.

6.4 Discontinuation of study medication

In case the study medication is. stopped for any reason, the Investigator will discuss the reasons with the Sponsor and a decision will be made as to whether the subject is withdrawn from the study or receives further treatment with PRO051 following an individual subject risk: benefit assessment.

6.5 Study medication accountability

The sponsor or designate will request the investigator or responsible pharmacist to sign a receipt for the study drugs. All study medication supplied for the study should be kept in a locked secure place with appropriate pharmaceutical precautions.

A “Drug Accountability” record should be maintained by the person responsible for dispensing the trial medication to the subject. This should record which supplies are issued to which subjects and any drugs returned unused. Details of any supplies that are inadvertently damaged should be given on this record, which will be collected by the monitor at the end of the study.

All unused study medication should be kept and added to the drug accountability record. All study medication in these categories will be inventoried by the monitor during and at the conclusion of the study. The monitor will arrange for their secure disposal at the end of the study.

The drugs supplied for this study are only intended for use by subjects in this study. They must not be diverted for use by others.

7 Adverse event reporting and follow up

7.1 Definitions

7.1.1 Adverse event

Any untoward medical occurrence in a subject or clinical investigation subject, temporally associated with the use of a medicinal product, whether or not considered related to the medicinal product.

Note: An AE can therefore be any unfavorable and unintended sign (including an abnormal laboratory finding), symptom, or disease (new or exacerbated) temporally associated with the use of a medicinal product. For marketed medicinal products, this also includes failure to produce expected benefits (i.e., lack of efficacy), abuse or misuse.

Events meeting the definition of an AE include:

- Exacerbation of a chronic or intermittent pre-existing condition including either an increase in frequency and/or intensity of the condition
- New conditions detected or diagnosed after investigational product administration even though it may have been present prior to the start of the study
- Signs, symptoms, or the clinical sequelae of a suspected interaction
- Signs, symptoms, or the clinical sequelae of a suspected overdose of either investigational product or a concomitant medication (overdose per se will not be reported as an AE/SAE).
- “Lack of efficacy” or “failure of expected pharmacological action” per se will not be reported as an AE or SAE. However, the signs and symptoms and/or clinical sequelae resulting from lack of efficacy will be reported if they fulfil the definition of an AE or SAE.

Events that **do not** meet the definition of an AE include:

- Medical or surgical procedure (e.g., endoscopy, appendectomy); the condition that leads to the procedure is an AE
- Situations where an untoward medical occurrence did not occur (social and/or convenience admission to a hospital)
- Anticipated day-to-day fluctuations of pre-existing disease(s) or condition(s) present or detected at the start of the study that do not worsen
- The disease/disorder being studied, or expected progression, signs, or symptoms of the disease/disorder being studied, unless more severe than expected for the subject’s condition.

7.1.2 Serious Adverse Events

A serious adverse event is any untoward medical occurrence that, at any dose:

a. Results in death

b. Is life-threatening

NOTE: The term 'life-threatening' in the definition of 'serious' refers to an event in which the subject was at risk of death at the time of the event. It does not refer to an event, which hypothetically might have caused death, if it were more severe.

c. Requires hospitalization or prolongation of existing hospitalization

NOTE: In general, hospitalization signifies that the subject has been detained (usually involving at least an overnight stay) at the hospital or emergency ward for observation and/or treatment that would not have been appropriate in the physician's office or out-patient setting. Complications that occur during hospitalization are AEs. If a complication prolongs hospitalization or fulfills any other serious criteria, the event is serious. When in doubt as to whether "hospitalization" occurred or was necessary, the AE should be considered serious.
Hospitalization for elective treatment of a pre-existing condition that did not worsen from baseline is not considered an AE.

d. Results in disability/incapacity, or

NOTE: The term disability means a substantial disruption of a person's ability to conduct normal life functions. This definition is not intended to include experiences of relatively minor medical significance such as uncomplicated headache, nausea, vomiting, diarrhea, influenza, and accidental trauma (e.g. sprained ankle) which may interfere or prevent everyday life functions but do not constitute a substantial disruption.

e. Is a congenital anomaly/birth defect

f. Medical or scientific judgement should be exercised in deciding whether reporting is appropriate in other situations, such as important medical events that may not be immediately life-threatening or result in death or hospitalization but may jeopardize the subject or may require medical or surgical intervention to prevent one of the other outcomes listed in the above definition. These should also be considered serious. Examples of such events are invasive or malignant cancers, intensive treatment in an emergency room or at home for allergic bronchospasm, blood dyscrasias or convulsions that do not result in hospitalization, or development of drug dependency or drug abuse.

g. All events of possible drug-induced liver injury as stated in section 5.6.8.1

Medical and scientific judgment should be exercised in deciding whether expedited reporting is appropriate in other situations, such as important medical events that may not be immediately life-threatening or result in death or hospitalization but may jeopardize the subject or may require intervention to prevent one of the other outcomes listed in the definition above. These should also be considered serious.

A death occurring during the study or which comes to the attention of the investigator within 30 days of study drug administration, whether considered treatment related or not, must be reported.

In the event of an SAE the investigator may immediately stop treatment if it is considered in the best interest of the subject. In case a serious adverse event related to the study medication occurs, the subject must be withdrawn, unless doing so would harm the subject in the opinion of the investigator.

7.1.3 Suspected Unexpected Serious Adverse Reactions (SUSAR)

A SUSAR is any adverse reaction that is classed as serious and is suspected to be caused by the investigational medicinal product and is ***NOT*** consistent with the information about the investigational medicinal product in either the IB or SmPC.

The process for reporting of SUSARs is described in the study specific pharmacovigilance working instructions describing the details and persons involved.

7.2 Reporting of adverse events

At each evaluation, the investigator will determine whether any adverse events have occurred. If any adverse events have occurred, they will be recorded on the adverse event report pages of the CRF. If known, the diagnosis should be recorded, in preference to the listing of individual signs and symptoms.

Any adverse event experienced by the subject from the time of signing the informed consent until 30 days after the end of the study will be recorded on the CRF.

7.2.1 Evaluation of adverse events

7.2.1.1 Intensity/severity grade

The investigator will be asked to assess the severity of the adverse event using the following categories: mild, moderate and severe. This assessment is subjective and the investigator should use medical judgment to compare the reported adverse event to similar type events observed in clinical practice.

Below are listed guidelines for severity assessment:

- **Mild**: Symptom(s) barely noticeable to the subject or does not make the subject uncomfortable. The adverse event does not influence performance or functioning. Prescription drugs are not ordinarily needed for relief of symptom(s).
- **Moderate**: Symptom(s) of a sufficient severity to make the subject uncomfortable. Performance of daily activities is influenced. Treatment of symptom(s) may be needed.
- **Severe**: Symptom(s) of a sufficient severity to cause the subject severe discomfort. Severity may cause cessation of treatment with the study drug. Treatment for symptom(s) may be given.

7.2.1.2 Assessment of Causality

The investigator will make a judgment regarding whether or not, in his/her opinion, the adverse event was related to study agent. The investigator will also evaluate any changes in laboratory values, make a determination as to whether the change is clinically important, and whether or not the changes were related to study drug. However, even if the investigator feels there is no relationship to the study drug, the adverse event or laboratory abnormality must be recorded in the CRF.

Causality criteria are defined as follows:

- **Not related**: the adverse event must definitely be caused by the subject's clinical state, or the study procedure/conditions, or concomitant medication.
- **Remotely/unlikely related**: the study agent is not likely to have any reasonable association with the adverse event.
- **Possibly related**: an adverse event could have been produced by the study agent.
- **Probably related**: the adverse event follows a reasonable temporal sequence from the time of study agent administration, abates upon discontinuation of the study agent and cannot be reasonably explained by the known characteristics of the subject's clinical state.
- **Definitely related**: the adverse event follows a reasonable temporal sequence from the time of study agent administration, abates upon discontinuation of the study agent and reappears when the study agent is introduced.

7.3 Reporting of serious adverse events

Investigators are obliged to notify, by fax (or e-mail), to the sponsor's delegate all serious adverse events **IMMEDIATELY** (within **24 hours** of the investigator becoming aware of the event) by faxing in or e-mail the SAE report form and its applicable attachments. The investigator will be requested to supply as much detailed information regarding the event that is available at the time of the initial contact. The investigator is also required to submit follow-up reports until the adverse event has resolved or in the case of permanent impairment, until the adverse event stabilizes.

ANY SERIOUS ADVERSE EVENT WHETHER OR NOT RELATED TO THE STUDY DRUG MUST BE REPORTED IMMEDIATELY TO THE FOLLOWING PERSON:

Contact name: ***TFS Trial Form Support***

Fax: ***+46 (0)46 31 33 88***

E-mail: ***safety.tfs@trialformsupport.com***

Telephone: ***+46 (0)46 31 32 00***

For all **fatal or life-threatening** SAEs, a complete, fully legible and signed SAE report form (in English) as well as all mandatory CRF pages (AE (if not sent within 24 hours), Medical History, Concomitant Medication, and laboratory), and other relevant CRF pages and anonymized copies of relevant subject records, autopsy reports, and other documents should be sent to the above mentioned fax number or e-mail address, within **3 calendar days** after first becoming aware of the event.

For all **other** SAEs, a complete, fully legible and signed SAE report form (in English) as well as all mandatory CRF pages (AE (if not sent within 24 hours), Medical History, Concomitant Medication, and laboratory), and other relevant CRF pages and anonymized copies of relevant subject records, autopsy reports, and other documents should be sent to the above mentioned fax number or e-mail address, within **6 calendar days** after first becoming aware of the event.

The responsible pharmacovigilance officer will immediately evaluate the SAEs for reporting to the appropriate regulatory agencies in consultation with the medical responsible person from the sponsor.

All serious, unexpected and related adverse events must also be reported to the reviewing IEC/IRB by the investigator. Confirmation that these reports have been submitted to the ethics committees must be forwarded to the monitor.

7.4 Adverse event follow up

If any adverse events considered to be possibly or probably related to study drug are present when the subject completes or is withdrawn from the study, the subject will be re-evaluated until resolution or until the adverse event has stabilized.

8 Data management, statistical analysis and reporting

8.1 Recording of data

All data will be recorded in a timely manner (e.g. within 24 hrs) in the Case Report Form (CRF), which will be provided by the sponsor.

Conducting a trial and the related use of CRFs should not detract from the routine data recording in the source documents. It should be clearly marked in the medical records of the subject at the trial site that the subject is participating in a particular clinical trial. Source data must be available to document the existence of the subject and substantiate integrity of trial data collected. Source data must include the original documents related to the trial (e.g. ECG traces, laboratory prints), to the medical treatment, and history of subject.

The following information should be included in the source medical records:

- demographic data
- medical history and physical examination details
- adverse events and concurrent treatment(s)
- biochemistry/hematology/urinalysis data
- effect data
- visit dates and dispensing of study medication
- information on the subject's treatment
- FVC, FEV_1, + in Extension part of study; mouth pressure exhale, mouth pressure inhale, peak flow, peak cough flow

The following information may be recorded directly on the CRF and should be considered as source data:

- vital signs
- local tolerability
- MMT (Dose escalation part of study only)

8.2 Study monitoring

The sponsor of this study is responsible according to ICH-GCP guidelines for assuring proper study conduct with regard to protocol adherence and validity of the data recorded on the CRFs.

The sponsor has therefore assigned a study monitor to this study. His/her duties are to assist the investigator in the maintenance of complete, legible, well-organized, and easily retrievable data. In addition, the monitor will ensure that the investigator understands all applicable regulations concerning the clinical evaluation of an investigational drug, as laid down in ICH GCP guidelines.

The investigator agrees to allow the monitor direct access to the study drug dispensing and storage area and to all clinical data of the study subjects for the above purposes and agrees to assist the monitor in these activities. The investigator accepts that the monitor will visit the centre at regular intervals to review and verify the data collected. The monitor will regard all information that is supplied to him or her as strictly confidential.

8.3 Safety monitoring

Safety monitoring will be performed by the internal medical monitor throughout the entire study and by the external Data Monitoring Committee (DMC) during the core part of the study. Any safety concerns will be communicated to the Sponsor. The DMC will be an

independent committee of 3 to 6 members. None of the members will be participating in the current trial. The major function of this committee will be to monitor the safety of the study agent. The DMC will periodically review tabulated safety summaries and any additional safety data the DMC may request during the conduct of the trial. Prior to any DMC review, the DMC charter will define and document the content of the safety summaries, the DMC's role and responsibilities, and the general procedures (including communications).

Following completion of the core part of the study the DMC was superseded by the Internal Safety Assessment Board (ISAB).

Independent Data Monitoring Committee (IDMC)

Subsequent to the core dose escalation phase, the extension phase data has been regularly reviewed in conjunction with the investigators, the medical safety monitor and the clinical project manager. In addition, an Internal Safety Assessment Board is in place, comprising medical, clinical and statistical representatives from Prosensa and GSK. The Board meets on a monthly basis to review key safety data. An Independent Data Monitoring Committee (IDMC) will be utilized in the GSK2402968 (originally PRO051) study program to ensure the external objective medical and/or statistical review of safety issues in order to protect the ethical and safety interests of subjects and to protect the scientific validity of the entire GSK2402968 (originally PRO051) development program. The IDMC comprises of independent consultants with relevant expertise, who meet to review the safety data across the programme on a regular basis. Members of this committee include a pediatrician with expertise in DMD, a professor of rare diseases, a statistician with expertise in DMD and a pediatric nephrologist.

8.4 Data management

Queries will be issued, e.g. on missing data, inconsistencies, illegible data, illegal values and unclearly corrected items. Resolution of queries will be implemented in the database.

The sponsor may resolve the following type of queries:

- Incorrect rounding of figures, unless rounding influences other data.
- Spelling and translation errors, if obvious.

Any other queries will be resolved by the investigator.

8.5 Statistical methods

8.5.1 General statistical methods

Subjects will be used as their own reference comparing pre- (baseline) and post-treatment.

Descriptive statistics will be used for description of the frequency and nature of the adverse events. The number and frequency of all adverse events and the adverse events considered possibly or probably related to study will be listed by dose, cumulative dose, body system and by coded term. In addition, the number of subjects experiencing an adverse event will be listed by dose, cumulative dose, body system and by coded term.

Quantitative (incl. biochemical, hematology, muscle strength) parameters will be expressed as mean ± SD, median, minimum and maximum per visit.

Pharmacokinetics and dose response relations will be evaluated using population and sparse data analysis.

The analyses will be performed according to the Statistical Analyses Plan that will be available before the database is locked. Deviations from the Statistical Analyses Plan will be justified in the study report.

8.5.2 Interim analyses

Not applicable.

8.5.3 Sample size justification

The sample size has been chosen on scientific/medical grounds rather than on statistical grounds, because of the low prevalence of DMD subjects carrying a mutation correctable by PRO051.

8.5.4 Evaluability of data

All subjects receiving at least one dose of study medication will be included in the safety analysis population.

All subjects receiving at least one dose of study medication, and in whom at least one effect parameter post baseline is available will be included in the ITT analysis population.

All subjects completing the protocol and in whom no major protocol violations have been noticed will be included in the per protocol population.

All subjects receiving at least one dose of study medication, and in whom at least one PK sample post baseline is available will be included in the pharmacokinetic analysis population.

8.5.5 Statistical analysis

A Statistical Analyses Plan will be prepared prior to database lock describing the data analysis in more detail.

8.5.5.1 Demographic and background variables

Baseline demographic and background variables will be summarized for all subjects. For categorical variables, frequencies and percentages will be provided. For continuous variables, descriptive statistics including the sample size, mean, median, standard deviation and range, will be presented.

8.5.5.2 Subject accountability

All subjects enrolled will be included in a summary of subject accountability. The frequency and percentage of subjects enrolled in the study, received treatment, discontinued before completing the study, and completing the study will be summarized.

8.5.5.3 Study medication compliance

A summary of study medication usage and compliance will be provided for all subjects by treatment group.

8.5.5.4 Safety analysis

- Adverse events (AEs) will be coded and presented using MedDRA. The overall incidence, the incidence of possibly or probably related as well as the incidence of serious adverse events will be summarized per body system in tables. An adverse event that occurred during the study medication period or at follow-up will only be counted as an adverse event if it was either not present at baseline or it was present at baseline but increased in severity during the treatment period.
- Vital Signs (supine blood pressure and heart rate): Descriptive statistics (n, mean, standard deviation, median, minimum, and maximum) will be provided on blood pressure along with heart rate at baseline and all post-dose assessments.
- Hematology, urinalysis and clinical chemistry parameters: Descriptive statistics (n, mean, standard deviation, median, minimum, and maximum) will be provided on values at baseline and all post-dose assessments. Descriptive statistics will be calculated on the change from baseline measurements. Out-of-normal-range values will be indicated for each parameter. The change in out-of-normal-range values after administration and at follow up versus baseline will be summarized by means of shift-tables.
- ECG: Descriptive statistics (frequency, mean, standard deviation, median, minimum, and maximum) on ECG parameters will be provided at baseline and all post-baseline

assessments. Changes from baseline measurements will be calculated and descriptive statistics will be computed. Shift tables will also be provided to indicate changes in ECG parameters over time.

8.5.5.5 Pharmacodynamic analysis

Endogenous production of the expected mRNA (assessed by RT-PCR on RNA isolated from muscle biopsy and mononuclear blood cells) will be reported by means of RT-PCR and gel electrophoresis analysis (photograph), including sequence analysis of the resulting products to confirm the precise exon 51 skipping.

The presence and localization of novel dystrophin protein (assessed by immunofluorescence analysis and western blot analysis) will be reported. No formal analyses will be performed for dystrophin presence and localization.

All other effect parameters will be reported using descriptive statistics (frequency, mean, standard deviation, median, minimum, and maximum). Changes from baseline measurements will be calculated and descriptive statistics will be computed.

8.5.5.6 Pharmacokinetic analysis

Plasma concentration vs. time profiles of PRO051 will be analyzed. Pharmacokinetic parameters AUC, C_{max}, C_{trough}, t_{max}, clearance, volume of distribution and elimination half-life will be obtained. Linearity of PRO051 pharmacokinetics will be evaluated using ANOVA on log-transformed, dose normalized PK parameters.

8.5.6 Study parameters

8.5.6.1 Effect parameters

The following effect parameters will be assessed:

- Production of exon skip 51 mRNA (assessed by RT-PCR on RNA isolated from muscle biopsy and from mononuclear blood cells)
- Presence of dystrophin expression (assessed by immunofluorescence analysis of cross-sections derived from the muscle biopsy and by Western blot analysis of total protein extracts from the muscle biopsy)
- Muscle function (distance walked in 6 minutes and timed tests: 10-meter walk/run, rising from floor, stair climb)
- Muscle strength (QMT, MMT)

8.5.6.2 Safety parameters

The following safety parameters will be assessed:

- Frequency and number of adverse events
- Local tolerability
- Safety hematology and biochemistry parameters
- Coagulation (aPTT)
- Urinalysis
- Complement activation (complement split products C3a, SC5b-9, Bb)
- Inflammatory response (IL-6, TNF-α, MCP-1)
- Immune response to dystrophin (antibodies)
- ECG, heart rate and blood pressure

8.5.6.3 Pharmacokinetic parameters

The following pharmacokinetic parameters will be assessed:

- $t_{1/2}$
- AUC: 0-24h, 24h-7d, 0-∞
- $C_{max,}$ $C_{trough\ 7d}$
- t_{max}
- distribution volume
- clearance

8.6 Study report

At the conclusion of the study period, after the data are analyzed, the sponsor will prepare an integrated study report. A draft copy of the report will be available for review by the principal investigators.

At the conclusion of the treatment beyond study period, after the data are analyzed, the sponsor will prepare an additional study report. A draft copy of the report will be available for review by the principal investigators.

9 Administrative procedures

9.1 Basic principles

The study will be performed in accordance with the International Conference on Harmonisation Good Clinical Practice (ICH-GCP) requirements, the Declaration of Helsinki (see Appendix 1), current FDA regulations and guidelines, the European Clinical Trials Directive and associated guidelines, as well as all other applicable national and local laws. Written informed consent will be obtained for this study by the principal investigator or his/her designee from all subjects prior to any study-specific procedures

9.2 Ethical considerations

9.2.1 Independent Ethics Committee/Institutional Review Board approval

Written approval must be obtained from the IEC/IRB prior to release of clinical supplies and commencement of the study.

The protocol, the IB, IMPD, the subject information sheet, the consent form, the investigator’s curriculum vitae and the advertisements (if any) will be submitted to the applicable Independent Ethics Committee (IEC) or Institutional Review Board (IRB). The IEC/IRB must be constituted and working in compliance with ICH-GCP.

A copy of the documented approval must be included in the Trial Master File.

Any (non) substantial amendment to the protocol or subject information and consent form will be submitted to the IEC/IRB of the investigational centre and written approval will be obtained for substantial amendments prior to implementation.

9.2.2 Regulatory requirements

The study will only start after authorization has been received from the relevant Competent Authorities.

9.2.3 Insurance of the subject

The sponsor will cover this study by means of an adequate insurance of the subject which will be in place prior to the start of the study. As evidence of this insurance, a copy of the Insurance statement will be provided to the investigator for storage in the Investigator File.

9.2.4 Informed consent procedures

It is the responsibility of the Investigator to obtain written Informed Consent from the subject. Information about the study will be given to the subject both verbally and in writing. The written subject information sheet will explain the objectives of the study, its potential risks and benefits. The investigator must be satisfied that the subject has understood the information provided before written consent is obtained.

It should be made clear that refusal to participate or withdrawal from the trial at any stage is without any prejudice to the subject’s subsequent care. No subject should be obliged to participate in the trial. The subject must be given ample opportunity to enquire about details of the trial. If there is any doubt as to whether the subject has understood the written and verbal information, the subject should not enter the study.

The subject must be made aware that the monitors, auditors, the IEC/IRB and regulatory authorities will be granted direct access to the study subjects source medical records without violating subject confidentiality, and to the extent permitted by applicable regulations.

If the subject agrees to participate in the study he will be asked to sign and date a consent form which will be kept by the Investigator. The subject information leaflet and a copy of the signed informed consent will be provided to the subject.

The signed consent forms will be retained by the investigator and made available (for review only) to the study monitor and auditor on request.

9.2.5 Confidentiality of subject records

Clinical research personnel assigned by the sponsor will require direct access to the subject’s notes for source data verification. The confidentiality of all the subjects’ identities will be maintained.

Only subjects’ initials, date of birth and study number will be used on CRFs and in all study correspondence. No material bearing a subject’s name will be kept on file by the sponsor or clinical research personnel assigned by the sponsor.

All information disclosed to the investigator by the sponsor or persons assigned by the sponsor shall be treated by the investigator as strictly confidential. The Investigator shall only use such information for the purpose of conducting the clinical trial described in this protocol and the investigator agrees not to disclose such information to any third party except those of his colleagues and employees who are assisting in the conduct of the study and who are bound by the obligations of confidentiality.

Information concerning the study drug, patent applications, processes, unpublished scientific data, the Investigator's Brochure and other pertinent information is confidential and remains the property of the sponsor. Details should be disclosed only to the persons involved in the approval or conduct of the study. The investigator may use this information for the purpose of the study only. It is understood by the investigator that the sponsor will use the information obtained during the clinical study in connection with the development of the drug and therefore may disclose it as required to other clinical investigators or to regulatory agencies. In order to allow for the use of the information derived from this clinical study, the investigator understands that he has an obligation to provide the sponsor with all data obtained during the study.

9.3 Financial disclosure

In compliance with 21 CFR part 54, any listed or identified investigator or sub-investigator (including the spouse and any dependent children of said individuals) directly involved in the treatment or evaluation of research subjects will disclose the following information for the time period during which the investigator is participating in the study and for 1 year following completion of the study:

- Any financial arrangement entered into between the sponsor(s) and the investigator, whereby the value of the compensation to the investigator for conducting the study could be influenced by the outcome of the study;
- Any other significant payments totaling > € 5,000.00, exclusive of the costs of conducting this or other clinical studies, by the Sponsors such as a grant to fund ongoing research, compensation in the form of equipment, retainer for ongoing consultation, or honoraria;
- Any proprietary interest in the product being evaluated;
- Any significant equity interest, including ownership interest, stock options, or other financial interest whose value cannot be determined through reference to public prices or any equity interest in Prosensa Holding B.V. that exceeds € 5,000.00.

9.4 Publication policy

The study may be considered for publication or presentation at (scientific) symposia and congresses.

The investigator will be entitled to publish or disclose the results, only after review and approval of the sponsor. The sponsor will review all transcripts, texts of presentations and abstracts related to the study at least 30 days prior to the intended submission for publication or any other disclosure. This is necessary to prevent premature disclosure of trade secrets or

patent-protected information and is in no way intended to restrict publication of facts or opinions formulated by the investigator.

The sponsor will inform the investigator of any objection or question arising within 90 days of receipt of the proposed publication. Hereafter, the manuscript is free for publication.

Further details are documented in the Clinical Trial Agreement.

9.5 Record retention

The investigator will maintain a study file, which he/she should use to file the IB, protocol, drug accountability records, correspondence with the IEC/IRB, the sponsor and other study-related documents. A dedicated binder will be provided by the sponsor.

The investigator's copy of the CRFs, or print of the eCRFs, study file, consent forms, drug accountability records and the subject identification list must be kept by the investigator for at least 15 years after completion of the study. These documents should be retained for a longer period, however, if required by the sponsor or applicable regulatory requirements. It is the responsibility of the sponsor to inform the investigator/institution as to when the documents no longer need to be retained. In addition the investigator must make provision for the subjects' records to be kept for the same period of time.

The sponsor will archive and retain all documents pertaining to the study for at least 15 years after the last approval.

9.6 Protocol amendments

Any changes to the study, which arise after approval of the protocol, must be documented as protocol amendments. Protocol amendments affecting the safety of the subject, the scope of the study and/or the scientific quality (i.e. substantial amendments) should be submitted to regulatory authorities and to the IEC/IRB. For substantial amendments, the changes will become effective only after approval by the sponsor, the responsible investigator, IEC/IRB and competent authorities. All other amendments (i.e. non-significant or administrative amendments) will be submitted to the IEC/IRB and competent authorities for notification.

9.7 Quality control and quality assurance

The sponsor may conduct periodical audits of the study by reviewing the data obtained as well as the procedural aspects. This may include on-site inspections and source data checks. Direct access to source documents is required for the purpose of these periodical inspections. Any such access will be confidential and the identity of the subject will not appear on any copies made of the records.

9.8 Regulatory inspections

Regulatory authorities may also wish to have direct access to the medical records of subjects participating in the study for the purpose inspections. Any such access will be confidential and the identity of the subject will not appear on any copies made of the records. The inspection may occur during the study, or at any time following the study.

10 References

1. Emery AEH. Duchenne muscular dystrophy,. 2nd edn. ed. Oxford,: Oxford University Press; 1993.
2. Hoffman EP, Fischbeck KH, Brown RH, et al. Characterization of dystrophin in muscle-biopsy specimens from patients with Duchenne's or Becker's muscular dystrophy. N Engl J Med 1988;318(21):1363-8.
3. Koenig M, Monaco AP, Kunkel LM. The complete sequence of dystrophin predicts a rod-shaped cytoskeletal protein. Cell 1988;53(2):219-26.
4. Hoffman EP, Brown RH, Jr., Kunkel LM. Dystrophin: the protein product of the Duchenne muscular dystrophy locus. Cell 1987;51(6):919-28.
5. Koenig M, Beggs AH, Moyer M, et al. The molecular basis for Duchenne versus Becker muscular dystrophy: correlation of severity with type of deletion. Am J Hum Genet 1989;45(4):498-506.
6. Ervasti JM, Ohlendieck K, Kahl SD, Gaver MG, Campbell KP. Deficiency of a glycoprotein component of the dystrophin complex in dystrophic muscle. Nature 1990;345(6273):315-9.
7. Aartsma-Rus A, Van Deutekom JC, Fokkema IF, Van Ommen GJ, Den Dunnen JT. Entries in the Leiden Duchenne muscular dystrophy mutation database: an overview of mutation types and paradoxical cases that confirm the reading-frame rule. Muscle Nerve 2006;34(2):135-44.
8. Monaco AP, Bertelson CJ, Liechti-Gallati S, Moser H, Kunkel LM. An explanation for the phenotypic differences between patients bearing partial deletions of the DMD locus. Genomics 1988;2(1):90-5.
9. Pramono ZA, Takeshima Y, Alimsardjono H, Ishii A, Takeda S, Matsuo M. Induction of exon skipping of the dystrophin transcript in lymphoblastoid cells by transfecting an antisense oligodeoxynucleotide complementary to an exon recognition sequence. Biochem Biophys Res Commun 1996;226(2):445-9.
10. Aartsma-Rus A, Janson AA, Kaman WE, et al. Therapeutic antisense-induced exon skipping in cultured muscle cells from six different DMD patients. Hum Mol Genet 2003;12(8):907-14.
11. Alter J, Lou F, Rabinowitz A, et al. Systemic delivery of morpholino oligonucleotide restores dystrophin expression bodywide and improves dystrophic pathology. Nat Med 2006;12(2):175-7. Epub 2006 Jan 29.
12. Lu QL, Mann CJ, Lou F, et al. Functional amounts of dystrophin produced by skipping the mutated exon in the mdx dystrophic mouse. Nat Med 2003;6:6.
13. Lu QL, Rabinowitz A, Chen YC, et al. Systemic delivery of antisense oligoribonucleotide restores dystrophin expression in body-wide skeletal muscles. Proc Natl Acad Sci U S A 2005;102(1):198-203.
14. Aartsma-Rus A, Janson AA, Kaman WE, et al. Antisense-induced multiexon skipping for Duchenne muscular dystrophy makes more sense. Am J Hum Genet 2004;74(1):83-92. Epub 2003 Dec 16.
15. Aartsma-Rus A, Kaman WE, Weij R, den Dunnen JT, van Ommen GJ, van Deutekom JC. Exploring the frontiers of therapeutic exon skipping for Duchenne muscular dystrophy by double targeting within one or multiple exons. Mol Ther 2006;14(3):401-7. Epub 2006 Jun 6.
16. Bremmer-Bout M, Aartsma-Rus A, de Meijer EJ, et al. Targeted exon skipping in transgenic hDMD mice: A model for direct preclinical screening of human-specific antisense oligonucleotides. Mol Ther 2004;10(2):232-40.
17. Henry SP, Kim T-W, Kramer-Stickland K, Zanardi TA, Fey RA. Toxicologic Properties of 2’-Methoxyethyl Chimeric Antisense Inhibitors in Animals and Man. . In: Crooke ST, ed. Antisense Drug Technology: Principles Strategies, and Applications. Boca Raton: CRC Press, Taylor & Francis group; 2008:327-64.
18. Levin AA, Henry SP, Monteith D, Templin M. Toxicity of Antisense Oligonucleotides. In: Crooke ST, ed. Antisense Drug Technology: Principles, Strategies, and Applications. New York: Marcel Dekker, Inc.; 2001:201-67.

19. Kwoh JT. An Overview of the Clinical Safety Experience of First and Second Generation Antisense Oligonucleotides In: Crooke ST, ed. Antisense Drug Technology: Principles, Strategies, and Applications. 2nd ed. Boca Raton: CRC Press, Taylor & Francis Group; 2008:365-400.
20. Yu RZ, Geary RS, Ushiro-Watanabe T, Levin AA, Schoenfeld SL. Pharmacokinetic properties in humans. In: Crooke ST, ed. Antisense Drug Technology: Principles, Strategies, and Applications. New York: Marcel Dekker, Inc.; 2001:183-200.
21. van Deutekom J, Janson J, Ginjaar H, et al. Local Dystrophin Restoration with Antisense Oligonucleotide PRO051 N Engl J Med 2007;357(26):2677-86.
22. Mercuri E, Bushby K, Ricci E, et al. Muscle MRI findings in patients with limb girdle muscular dystrophy with calpain 3 deficiency (LGMD2A) and early contractures. Neuromuscul Disord 2005;15(2):164-71. Epub 2004 Nov 26.
23. Dorph C, Nennesmo I, Lundberg IE. Percutaneous conchotome muscle biopsy. A useful diagnostic and assessment tool. The Journal of rheumatology 2001;28(7):1591-9.
24. Escolar DM, Henricson EK, Mayhew J, et al. Clinical evaluator reliability for quantitative and manual muscle testing measures of strength in children. Muscle Nerve 2001;24(6):787-93.
25. Mayhew JE, Florence JM, Mayhew TP, et al. Reliable surrogate outcome measures in multicenter clinical trials of Duchenne muscular dystrophy. Muscle Nerve 2007;35(1):36-42.
26. Havenga MJ, Lemckert AA, Ophorst OJ, et al. Exploiting the natural diversity in adenovirus tropism for therapy and prevention of disease. J Virol 2002;76(9):4612-20.
27. Roest PA, Roberts RG, van der Tuijn AC, Heikoop JC, van Ommen GJ, den Dunnen JT. Protein truncation test (PTT) to rapidly screen the DMD gene for translation terminating mutations. Neuromuscul Disord 1993;3(5-6):391-4.
28. Aartsma-Rus A, Bremmer-Bout M, Janson A, den Dunnen J, van Ommen G, van Deutekom J. Targeted exon skipping as a potential gene correction therapy for Duchenne muscular dystrophy. Neuromuscul Disord 2002;12 Suppl:S71.
29. Braun S, Thioudellet C, Rodriguez P, et al. Immune rejection of human dystrophin following intramuscular injections of naked DNA in mdx mice. Gene Ther 2000;7(17):1447-58.

Appendix 1 Declaration of Helsinki

Adopted by the 18th WMA General Assembly, Helsinki, Finland, June 1964, and amended by the
29th WMA General Assembly, Tokyo, Japan, October 1975
35th WMA General Assembly, Venice, Italy, October 1983
41st WMA General Assembly, Hong Kong, September 1989
48th WMA General Assembly, Somerset West, Republic of South Africa, October 1996
52nd WMA General Assembly, Edinburgh, Scotland, October 2000
53rd WMA General Assembly, Washington 2002 (Note of Clarification on Paragraph 29 added)
55th WMA General Assembly, Tokyo 2004 (Note of Clarification on Paragraph 30 added)
59th WMA General Assembly, Seoul, October 2008

Also refer to http://www.wma.net/e/ for the most recent version.

A INTRODUCTION

1 The World Medical Association (WMA) has developed the Declaration of Helsinki as a statement of ethical principles for medical research involving human subjects, including research on identifiable human material and data.

The Declaration is intended to be read as a whole and each of its constituent paragraphs should not be applied without consideration of all other relevant paragraphs.

2 Although the Declaration is addressed primarily to physicians, the WMA encourages other participants in medical research involving human subjects to adopt these principles.

3 It is the duty of the physician to promote and safeguard the health of patients, including those who are involved in medical research. The physician's knowledge and conscience are dedicated to the fulfillment of this duty.

4 The Declaration of Geneva of the World Medical Association binds the physician with the words, "The health of my patient will be my first consideration," and the International Code of Medical Ethics declares that, "A physician shall act only in the patient's best interest when providing medical care."

5 Medical progress is based on research that ultimately must include studies involving human subjects. Populations that are underrepresented in medical research should be provided appropriate access to participation in research.

6 In medical research involving human subjects, the well-being of the individual research subject must take precedence over all other interests.

7 The primary purpose of medical research involving human subjects is to understand the causes, development and effects of diseases and improve preventive, diagnostic and therapeutic interventions (methods, procedures and treatments). Even the best current interventions must be evaluated continually through research for their safety, effectiveness, efficiency, accessibility and quality.

8 In medical practice and in medical research, most interventions involve risks and burdens.

9 Medical research is subject to ethical standards that promote respect for all human subjects and protect their health and rights. Some research populations are particularly vulnerable and need special protection. These include those who cannot give or refuse consent for themselves and those who may be vulnerable to coercion or undue influence.

10 Physicians should consider the ethical, legal and regulatory norms and standards for research involving human subjects in their own countries as well as applicable international norms and standards. No national or international ethical, legal or regulatory requirement should reduce or eliminate any of the protections for research subjects set forth in this Declaration.

B BASIC PRINCIPLES FOR ALL MEDICAL RESEARCH

11 It is the duty of the physicians who participate in medical research to protect the life, health, dignity, integrity, right to self-determination, privacy, and confidentiality of personal information of research subjects.

12 Medical research involving human subjects must conform to generally accepted scientific principles, be based on a thorough knowledge of the scientific literature, other relevant sources of information, and adequate laboratory and, as appropriate, animal experimentation. The welfare of animals used for research must be respected

13 Appropriate caution must be exercised in the conduct of medical research that may harm the environment.

14 The design and performance of each research study involving human subjects must be clearly described in a research protocol. The protocol should contain a statement of the ethical considerations involved and should indicate how the principles in this Declaration have been addressed. The protocol should include information regarding funding, sponsors, institutional affiliations, other potential conflicts of interest, incentives for subjects and provisions for treating and/or compensating subjects who are harmed as a consequence of participation in the research study. The protocol should describe arrangements for post-study access by study subjects to interventions identified as beneficial in the study or access to other appropriate care or benefits

15 The research protocol must be submitted for consideration, comment, guidance and approval to a research ethics committee before the study begins. This committee must be independent of the researcher, the sponsor and any other undue influence. It must take into consideration the laws and regulations of the country or countries in which the research is to be performed as well as applicable international norms and standards but these must not be allowed to reduce or eliminate any of the protections for research subjects set forth in this Declaration. The committee must have the right to monitor ongoing studies. The researcher must provide monitoring information to the committee, especially information about any serious adverse events. No change to the protocol may be made without consideration and approval by the committee.

16 Medical research involving human subjects must be conducted only by individuals with the appropriate scientific training and qualifications. Research on patients or healthy volunteers requires the supervision of a competent and appropriately qualified physician or other health care professional. The responsibility for the protection of research subjects must always rest with the physician or other health care professional and never the research subjects, even though they have given consent.

17 Medical research involving a disadvantaged or vulnerable population or community is only justified if the research is responsive to the health needs and priorities of this population or community and if there is a reasonable likelihood that this population or community stands to benefit from the results of the research.

18 Every medical research study involving human subjects must be preceded by careful assessment of predictable risks and burdens to the individuals and communities involved in the research in comparison with foreseeable benefits to them and to other individuals or communities affected by the condition under investigation.

19 Every clinical trial must be registered in a publicly accessible database before recruitment of the first subject.

20 Physicians may not participate in a research study involving human subjects unless they are confident that the risks involved have been adequately assessed and can be satisfactorily managed. Physicians must immediately stop a study the risks are found to outweigh the potential benefits or when there is conclusive proof of positive and beneficial results.

21 Medical research involving human subjects may only be conducted if the importance of the objective outweighs the inherent risks and burdens to the research subjects.

22 Participation by competent individuals as subjects in medical research must be voluntary. Although it may be appropriate to consult family members or community leaders, no competent individual may be enrolled in a research study unless he or she freely agrees.

23 Every precaution must be taken to protect the privacy of research subjects and the confidentiality of their personal information and to minimize the impact of the study on their physical, mental and social integrity.

24 In medical research involving competent human subjects, each potential subject must be adequately informed of the aims, methods, sources of funding, any possible conflicts of interest, institutional affiliations of the researcher, the anticipated benefits and potential risks of the study and the discomfort it may entail, and any other relevant aspects of the study. The potential subject must be informed of the right to refuse to participate in the study or to withdraw consent to participate at any time without reprisal. Special attention should be given to the specific information needs of individual potential subjects as well as to the methods used to deliver the information. After ensuring that the potential subject has understood the information, the physician or another appropriately qualified individual must then seek the potential subject's freely-given informed consent, preferably in writing. If the consent cannot be expressed in writing, the non-written consent must be formally documented and witnessed.

25 For medical research using identifiable human material or data, physicians must normally seek consent for the collection, analysis, storage and/or reuse. There may be situations where consent would be impossible or impractical to obtain for such research or would pose a threat to the validity of the research. In such situations the research may be done only after consideration and approval of a research ethics committee.

26 When seeking informed consent for participation in a research study the physician should be particularly cautious if the potential subject is in a dependent relationship with the physician or may consent under

duress. In such situations the informed consent should be sought by an appropriately qualified individual who is completely independent of this relationship.

27 For a potential research subject who is deemed incompetent, the physician must seek informed consent from the legally authorized representative. These individuals must not be included in a research study that has no likelihood of benefit for them unless it is intended to promote the health of the population represented by the potential subject, the research cannot instead be performed with competent persons, and the research entails only minimal risk and minimal burden.

28 When a potential research subject who is deemed incompetent is able to give assent to decisions about participation in research, the investigator must seek that assent in addition to the consent of the legally authorized representative. The potential subject’s dissent should be respected.

29 Research involving subjects who are physically or mentally incapable of giving consent, for example, unconscious patients, may be done only if the physical or mental condition that prevents giving informed consent is a necessary characteristic of the research population. In such circumstances the physician should seek informed consent from the legally authorized representative. If no such representative is available and if the research cannot be delayed, the study may proceed without informed consent provided that the specific reasons for involving subjects with a condition that renders them unable to give informed consent have been stated in the research protocol and the study has been approved by a research ethics committee. Consent to remain in the research should be obtained as soon as possible from the subject or a legally authorized representative.

30 Authors, editors and publishers all have ethical obligations with regard to the publication of the results of research. Authors have a duty to make publicly available the results of their research on human subjects and are accountable for the completeness. Negative and inconclusive as well as positive results should be published or otherwise made publicly available. Sources of funding, institutional affiliations and conflicts of interest should be declared in the publication. Reports of research not in accordance with the principles of this Declaration should not be accepted for publication.

C ADDITIONAL PRINCIPLES FOR MEDICAL RESEARCH COMBINED WITH MEDICAL CARE

31 The physician may combine medical research with medical care only to the extent that the research is justified by its potential preventive, diagnostic or therapeutic value and if the physician has good reason to believe that participation in the research study will not adversely affect the health of the patients who serve as research subjects.

32 The benefits, risks, burdens and effectiveness of a new intervention must be tested against those of the best current proven intervention, except in the following circumstances:

- The use of placebo, or no treatment, is acceptable in studies where no current proven intervention exists; or
- Where for compelling and scientifically sound methodological reasons the use of placebo is necessary to determine the efficacy or safety of an intervention and the patients who receive placebo or no treatment will not be subject to any risk of serious or irreversible harm. Extreme care must be taken to avoid abuse of this option.

33 At the conclusion of the study, patients entered into the study are entitled to be informed about the outcome of the study and to share any benefits that result from it, for example, access to interventions identified as beneficial in the study or to other appropriate care or benefits

34 The physician must fully inform the patient which aspects of the care are related to the research. The refusal of a subject to participate in a study or the patient’s decision to withdraw from the study must never interfere with the patient-physician relationship.

35 In the treatment of a patient, where proven interventions do not exist or have been ineffective, the physician, after seeking expert advice, with informed consent from the patient or a legally authorized representative, may use an unproven intervention if in the physician's judgement it offers hope of saving life, re-establishing health or alleviating suffering. Where possible, this intervention should be made the object of research, designed to evaluate its safety and efficacy. In all cases, new information should be recorded and, where appropriate, made publicly available.

Appendix 2 Schedules of assessments

Table 4. Schedule of assessments in study period

Visit	1	1a	2	3	4	5	6	7	8	9	10	11	12	Withdrawal[1]
Day			1	8	15	22	29	36±3	43±3	57±3	78±3	99±3	120±3	
Week			1	2	3	4	5	6	7	9	12	15	18	
Period	Screening[2]		Treatment					Follow up						
Informed consent	x[3]													
Study medication administration			x	x	x	x	x							
Hospitalisation			x	x	x	x	x							
Demographics	x													
Medical history	x													
MRI[4]	x													
Skin biopsy[5,6]	x													
Mononuclear blood cells	x													
Physical examination	x		x	x	x	x	x		x		x		x	x
Weight, height	x								x				x	
Vital signs[7]	x		x	x	x	x	x	x	x		x		x	x
Safety biochemistry	x	x	x	x	x	x	x	x		x			x	x
Safety hematology	x	x	x	x	x	x	x	x		x			x	x
Urinalysis	x	x	x	x	x	x	x	x		x			x	
aPTT			x				x							
C3a / SC5b-9 / Bb			x				x							
IL-6 / TNF-α / MCP-1			x				x							
Dystrophin antibodies			x						x				x	x
Mononuclear blood cells			x	x		x		x	x					
ECG	x		x				x						x	x
Muscle biopsy	x[8]								x[9]		x[10]			x[11]
Timed tests[12]	x	x	x	x	x	x	x	x	x		x		x	x
6-Minutes walk	x								x				x	x
QMT, incl FVC/FEV_1	x	x	x	x	x	x	x	x	x		x		x	x
MMT	x								x				x	x
Local tolerability			x	x	x	x	x	x						
Adverse events	x	x	x	x	x	x	x	x	x	x	x	x	x	x
Concomitant medication	x	x	x	x	x	x	x	x	x	x	x	x	x	x
Pharmacokinetic sampling			x[13]	x[13]	x[13]	x[13]	x[13]	x	x	x	x	x	x	x

1. Visit to be performed if subject stops prematurely (for any reason)
2. All screening assessments are to be completed within 4 weeks prior to the first study medication administration, unless specified otherwise.
3. To be completed prior to any other screening procedure.
4. To be completed within 6 weeks prior to the first study medication administration.
5. To be completed at 6 weeks prior to the first study medication administration.
6. To be performed if a muscle cell or fibroblast culture is not previously established and analyzed at RNA level for presence of DMD gene exon mutation(s) correctable by exon 51 skipping

7. Vital signs include blood pressure, pulse and respiratory rate
8. Cohort 1 only
9. Cohort 1, 2, 3 and 4t
10. Cohort 2, 3 and 4
11. Only if withdrawal is before the two muscle biopsies have been collected
12. Timed tests consist of: 10-meter walk/run, rising from floor, stair climb
13. See Table 5 for the time points

Table 5. Schedule of assessments during each hospitalization (visit 2-6) in study period

Hour	t=-3[1]	t=0	t=0.5	t=2	t=3	t=4	t=6	t=9	t=12	t=24±3	t=96±6
											telephone call[2]
Time window (min)			± 5	± 15	± 15	± 15	± 15	± 30	± 30		
Study medication administration		x									
Physical examination	x									x	
Vital signs[3]	x				x			x		x	
Safety biochemistry	x										
Safety hematology	x										
Urinalysis	x										
aPTT	x[4]				x[4]		x[4]			x[4]	
C4a / SC5b-9 / Bb	x[4]				x[4]		x[4]			x[4]	
IL-6 / TNF-α / MCP-1	x[4]				x[4]		x[4]			x[4]	
Dystrophin antibodies	x[5]										
Mononuclear blood cells	x[6]										
ECG	x[4]				x[4]					x[4,7]	
Timed tests[8]	x[9]									x[4]	
QMT, incl FVC/FEV_1	x[9]									x[4]	
Pharmacokinetic sampling	x		x[4]	x[4]	x[4]	x[4]	x[4]	x[4]	x[4]	x[4]	
Local tolerability	x		x		x					x	
Adverse events	x---x										x
Concomitant medication	x---x										x

1. Baseline assessments will be done at time point 3 to 0 hours prior to dosing
2. The investigator will contact the subject/parent two days after release from the institute to enquiry about the health of the subject and to assess the adverse events and concomitant medication.
3. Vital signs include blood pressure, pulse and respiratory rate
4. At visit 2 (day 1) and visit 6 (day 29) only
5. At visit 2 (day 1) only
6. At visit 2 (day 1), visit 3 (day 8) and visit 5 (day 22) only
7. Optional
8. Timed tests consist of: 10-meter walk/run, rising from floor, stair climb
9. At visit 3 (day 8), visit 4 (day 16) and visit 5 (day 22) only

Table 6. Schedule of assessments for visits 13 – 37 (treatment beyond study period)

**Visit**	**13[1]**	**14**	**15**	**16**	**17**	**18**	**19**	**20**	**21**	**22**	**23**	**24**	**25**	**26**	**27**	**28**	**29**	**30**	**31**	**32**	**33**	**34**	**35**	**36**	**37**	**Withdrawal prior to visit 37**
**Day (window ± 1 day)**	d[1]	d+7	d+14	d+21	d+28	d+35	d+42	d+49	d+56	d+63	d+70	d+77	d+84	d+91	d+98	d+105	d+112	d+119	d+126	d+133	d+140	d+147	d+154	d+161	d+168	
**Week**	w[1]	w+1	w+2	w+3	w+4	w+5	w+6	w+7	w+8	w+9	w+10	w+11	w+12	w+13	w+14	w+15	w+16	w+17	w+18	w+19	w+20	w+21	w+22	w+23	w+24	
Period	Treatment beyond study																									
**Informed consent[2]**	x																									
**Hospitalisation**																					x					
**Study medication administration[3]**	x	x	x	x	x	x	x	x	x	x	x	x	x	x	x	x	x	x	x	x	x	x	x	x	x	
**Medical history**	x																									
**Physical examination**	x				x				x				x				x				x				x	x
**Vital signs[4]**	x[5]				x[5]				x[5]				x[5]				x[5]				x[5]				x[5]	x
**Weight, height**	x												x												x	
**ECG**	x[5]				x				x				x[5]				x				x				x	x
**Echocardiography[6]**	x																								x	x
**Urine dipstick**	x	x	x	x	x	x	x	x	x	x	x	x	x	x	x	x	x		x		x		x		x	x
**Urine protein/creatinine**	x	x	x	x	x	x	x	x	x	x	x	x	x	x	x	x	x		x		x		x		x	x
**Urine α-1-microglobulin**	x												x												x	x
**Safety biochemistry**	x				x				x				x				x				x				x	x
**Safety haematology**	x				x				x				x				x				x				x	x
**Thrombocyte count[7]**			x				x				x				x				x				x			
**Complement (C3)**	x[5]				x[5]				x[5]				x[5]				x[5]				x[5]				x[5]	x
**Troponin**	x				x				x				x				x				x				x	x
**Fibrinogen**	x				x				x				x				x				x				x	x
**Haptoglobin**	x				x				x				x				x				x				x	x
**CRP**	x				x				x				x				x				x				x	x
**aPTT**	x[5]				x[5]				x[5]				x[5]				x[5]				x[5]				x[5]	x
**Cystatin C**	x												x												x	x
**Pharmacokinetic sampling**	x[5]				x[5]				x[5]				x[5]				x[5]				x[8]				x[5]	x
**Dystrophin antibodies**	x												x												x	x
**Muscle biopsy**																									x[9]	x
**Timed tests[10]**	x								x				x				x				x				x	x
**6-Minutes walk**	x												x												x	x
**Handheld myometry**	x								x				x				x				x				x	x
**Spirometry[11]**	x								x				x				x				x				x	x
**Adverse events**	x	x	x	x	x	x	x	x	x	x	x	x	x	x	x	x	x	x	x	x	x	x	x	x	x	x
**Concomitant medication**	x	x	x	x	x	x	x	x	x	x	x	x	x	x	x	x	x	x	x	x	x	x	x	x	x	x
**Phone call / e-mail / SMS[12]**	x	x	x	x	x	x	x	x	x	x	x	x	x	x	x	x	x									

1. Start of the treatment beyond study period will not be prior to patient and/or his parents, the investigator and the sponsor have all agreed that drug administration appears to improve the clinical status of the patient
2. To be completed prior to any other study procedure for the treatment beyond study period.
3. Other assessments to be performed prior to study medication administration unless specified otherwise

4. Vital signs include temperature, blood pressure, pulse and respiratory rate
5. Prior to study medication administration and at t = 3 hr (± 15 min) after study medication administration
6. Parameters will be derived from M-mode measurements and include left ventricular ejection fraction, fractional shortening, left ventricular posterior wall thickness, left ventricular septum wall thickness, left ventricular end systolic diameter, left ventricular end diastolic diameter, and left ventricular ejection fraction modified Simpson's
7. Via finger prick
8. Prior to study medication administration and at t = 0.5 hr (± 5 min), 2 hr (± 15 min), 3 hr (± 15 min), 4 hr (± 15 min), 6 hr (± 15 min), 9 hr (± 30 min), 12 hr (± 30 min) and 24 hr (± 3 hrs) after study medication administration
9. Window is ± 3 days
10. Timed tests consist of: 10-meter walk/run, rising from floor, stair climb
11. Parameters include FVC, FEV_1, mouth pressure inhale, mouth pressure exhale, peak flow, peak cough flow
12. The investigator or study coordinator will contact subject/parent at four days after study medication administration (t = 96 ± 9 hrs) to enquiry about the health of the subject and to assess the adverse events and concomitant medication. In case patient and/or parent do not respond to e-mail/SMS, a phone call is still needed

Table 7. Schedule of assessments for visits 38 – 61 (treatment beyond study period)

**Visit**	**38**	**39**	**40**	**41**	**42**	**43**	**44**	**45**	**46**	**47**	**48**	**49**	**50**	**51**	**52**	**53**	**54**	**55**	**56**	**57**	**58**	**59**	**60**	**61**	**Withdrawal between visit**
**Day (window ± 1 day)**	d+175	d+182	d+189	d+196	d+203	d+210	d+217	d+224	d+231	d+238	d+245	d+252	d+259	d+266	d+273	d+280	d+287	d+294	d+301	d+308	d+315	d+322	d+329	d+336	
**Week**	w+25	w+26	w+27	w+28	w+29	w+30	w+31	w+32	w+33	w+34	w+35	w+36	w+37	w+38	w+39	w+40	w+41	w+42	w+43	w+44	w+45	w+46	w+47	w+48	
Period	Treatment beyond study																								
**Study medication administration**[1]	x	x	x	x	x	x	x	x	x	x	x	x	x	x	x	x	x	x	x	x	x	x	x	x	
**Physical examination**												x												x	x
**Vital signs**[2]												x												x	x
**Weight, height**												x												x	
**ECG**				x				x				x				x				x				x	x
**Echocardiography**[3]																								x	x
**Urine**[4]		x		x		x		x		x		x				x								x	x
**Urine protein/creatinine**		x		x		x		x		x		x				x								x	x
**Urine α-1-microglobulin**												x												x	x
**Safety biochemistry**				x				x				x				x								x	x
**Safety haematology**				x				x				x				x								x	x
**Thrombocyte count**[5]		x				x				x				x				x		x		x		x	
**Complement (C3)**				x				x				x				x				x				x	x
**Troponin**				x				x				x				x				x				x	x
**Fibrinogen**				x				x				x				x				x				x	x
**Haptoglobin**				x				x				x				x				x				x	x
**CRP**				x				x				x				x				x				x	x
**aPTT**				x				x				x				x				x				x	x
**Cystatin C**												x												x	x
**Dystrophin antibodies**																								x	x
**Timed tests**[6]				x								x												x	x
**6-Minutes walk**												x												x	x
**Handheld myometry**				x								x												x	x
**Spirometry**[7]				x								x												x	x
**Adverse events**	x	x	x	x	x	x	x	x	x	x	x	x	x	x	x	x	x	x	x	x	x	x	x	x	x
**Concomitant medication**	x	x	x	x	x	x	x	x	x	x	x	x	x	x	x	x	x	x	x	x	x	x	x	x	x

1. Other assessments to be performed prior to study medication administration unless specified otherwise
2. Vital signs include temperature, blood pressure, pulse and respiratory rate
3. Parameters will be derived from M-mode measurements and include left ventricular ejection fraction, fractional shortening, left ventricular posterior wall thickness, left ventricular septum wall thickness, left ventricular end systolic diameter, left ventricular end diastolic diameter, and left ventricular ejection fraction modified Simpson's
4. Collect a first morning urine specimen
5. Via finger prick
6. Timed tests consist of: 10-meter walk/run, rising from floor, stair climb
7. Parameters include FVC, FEV_1, mouth pressure inhale, mouth pressure exhale, peak flow, peak cough flow

Table 8. Schedule of assessments for visits 62 – 85 (treatment beyond study period)

**Visit**	**62**	**63**	**64**	**65**	**66**	**67**	**68**	**69**	**70**	**71**	**72**	**73**	**74**	**75**	**76**	**77**	**78**	**79**	**80**	**81**	**82**	**83**	**84**	**85**	**Withdrawal between visit**
**Day (window ± 1 day)**	d+343	d+350	d+357	d+364	d+371	d+378	d+385	d+392	d+397	d+406	d+413	d+420	d+427	d+434	d+441	d+448	d+455	d+462	d+469	d+476	d+483	d+490	d+497	d+504	
**Week**	w+49	w+50	w+51	w+52	w+53	w+54	w+55	w+56	w+57	w+58	w+59	w+60	w+61	w+62	w+63	w+64	w+65	w+66	w+67	w+68	w+69	w+70	w+71	w+72	
Period	Treatment beyond study																								
**Study medication administration[1]**	x	x	x	x	x	x	x	x	x	x	x	x	x	x	x	x	x	x	x	x	x	x	x	x	
**Physical examination**												x												x	x
**Vital signs[2]**												x												x	x
**Weight, height**												x												x	
**ECG**				x				x				x				x				x				x	x
**Echocardiography[3]**																								x	x
**Urine[4]**				x		x		x		x		x		x		x		x		x		x		x	x
**Urine protein/creatinine**				x		x[5]		x		x[5]		x		x[5]		x		x[5]		x		x[5]		x	x
**Urine α-1-microglobulin**				x				x				x				x				x				x	x
**Urine cystatin C**																				x				x	x
**KIM-1**																				x				x	x
**Safety biochemistry**				x				x				x				x				x				x	x
**Safety haematology**				x				x				x				x				x				x	x
**blood smear for schistocytes**																				x				x	x
**Thrombocyte count[6]**		x				x				x				x				x				x			
**Complement (C3)**				x				x				x				x				x				x	x
**MCP-1**																								x	
**Troponin**				x[7]				x[7]																	
**Fibrinogen**				x				x				x				x				x				x	x
**Haptoglobin**				x				x				x				x				x				x	x
**CRP**				x				x				x				x				x				x	x
**aPTT/ PTT (INR)**				x				x				x				x				x				x	x
**Cystatin C**												x												x	x
**Pharmacokinetic sampling[12]**																								x	x
**Dystrophin antibodies**																								x	x
**Muscle biopsy (optional)[8]**				x																					
**Timed tests[9]**												x												x	x
**6-Minutes walk**												x												x	x
**Handheld myometry**												x												x	x
**Spirometry[10]**												x												x	x
**DEXA scan[11]**				x	x	x	x	x	x	x	x	x	x	x	x	x	x	x	x	x	x	x	x	x	x
**Adverse events**	x	x	x	x	x	x	x	x	x	x	x	x	x	x	x	x	x	x	x	x	x	x	x	x	x
**Concomitant medication**	x	x	x	x	x	x	x	x	x	x	x	x	x	x	x	x	x	x	x	x	x	x	x	x	x

1. Other assessments to be performed prior to study medication administration unless specified otherwise
2. Vital signs include temperature, blood pressure, pulse and respiratory rate
3. Parameters will be derived from M-mode measurements and include left ventricular ejection fraction, fractional shortening, left ventricular posterior wall thickness, left ventricular septum wall thickness, left ventricular end systolic diameter, left ventricular end diastolic diameter, and left ventricular ejection fraction modified Simpson's
4. Collect a first morning urine specimen
5. Additional measurements at visits 67, 71, 75, 79 and 83 required with the implementation of Amendment 6 of this protocol
6. Via finger prick
7. The requirement for measuring troponin was removed with the implementation of Amendment 6 of this protocol and is not required at this visit
8. Optional muscle biopsy will be taken at visit 65 (week 52) or as soon as Amendment 6 of this protocol is implemented and convenient to the investigative site and subject
9. Timed tests consist of: 10-meter walk/run, rising from floor, stair climb
10. Parameters include FVC, FEV_1, mouth pressure inhale, mouth pressure exhale, peak flow, peak cough flow
11. A DEXA scan will be performed at visit 65 (week 52) or as soon Amendment 6 of this protocol is implemented and convenient to the investigative site and subject. A DEXA scan may be performed at any visit, if deemed clinically necessary by the Investigator (i.e. rapid weight gain)
12. Prior to study medication administration

Table 9. Schedule of assessments for visits 86 – 109 (treatment beyond study period)

Visit	86	87	88	89	90	91	92	93	94	95	96	97	98	99	100	101	102	103	104	105	106	107	108	109	Withdrawal between visit
**Day (window ± 1 day)**	d+511	d+518	d+525	d+532	d+539	d+546	d+553	d+560	d+567	d+574	d+581	d+588	d+595	d+602	d+609	d+616	d+623	d+630	d+637	d+644	d+651	d+658	d+665	d+672	
**Week**	w+73	w+74	w+75	w+76	w+77	w+78	w+79	w+80	w+81	w+82	w+83	w+84	w+85	w+86	w+87	w+88	w+89	w+90	w+91	w+92	w+93	w+94	w+95	w+96	
Period	Treatment beyond study																								
**Study medication administration[1]**									x	x	x	x	x	x	x	x					x	x	x	x	
**Physical examination**												x												x	x
**Vital signs[2]**												x												x	x
**Weight, height**												x												x	
**ECG**				x				x				x				x					x			x	x
**Echocardiography[3]**																								x	x
**Urine[4]**				x				x		x		x		x		x					x	x		x	x
**Urine protein/creatinine**				x				x		x		x		x		x					x	x		x	x
**Urine α-1-microglobulin**				x				x				x				x					x			x	x
**Urine cystatin C**				x				x				x				x					x			x	x
**KIM-1**				x				x				x				x					x			x	x
**Safety biochemistry**				x				x				x				x					x			x	x
**Safety haematology**				x				x				x				x					x			x	x
**Blood smear for schistocytes**				x				x				x				x					x			x	x
**Thrombocyte count[5]**										x				x								x			
**Complement (C3)**				x				x				x				x					x			x	x
**MCP-1**				x				x				x				x					x			x	x
**Fibrinogen**				x				x				x				x					x			x	x
**Haptoglobin**				x				x				x				x					x			x	x
**CRP**				x				x				x				x					x			x	x
**aPTT/ PTT (INR)**				x				x				x				x					x			x	x
**Cystatin C**												x												x	x
**Pharmacokinetic sampling[9]**				x				x				x				x					x			x	x
**Dystrophin antibodies**																								x	
**Timed tests[6]**								x													x				x
**6-Minutes walk**								x													x				x
**Handheld myometry**								x													x				x
**Spirometry[7]**								x													x				x
**DEXA scan[8]**	x	x	x	x	x	x	x	x	x	x	x	x	x	x	x	x	x	x	x	x	x	x	x	x	x
**Adverse events[10]**	x	x	x	x	x	x	x	x	x	x	x	x	x	x	x	x	x	x	x	x	x	x	x	x	x
**Concomitant medication[11]**	x	x	x	x	x	x	x	x	x	x	x	x	x	x	x	x	x	x	x	x	x	x	x	x	x

1. Other assessments to be performed prior to study medication administration unless specified otherwise
2. Vital signs include temperature, blood pressure, pulse and respiratory rate
3. Parameters will be derived from M-mode measurements and include left ventricular ejection fraction, fractional shortening, left ventricular posterior wall thickness, left ventricular septum wall thickness, left ventricular end systolic diameter, left ventricular end diastolic diameter, and left ventricular ejection fraction modified Simpson's

4. Collect a first morning urine specimen
5. Via finger prick
6. Timed tests consist of: 10-meter walk/run, rising from floor, stair climb
7. Parameters include FVC, FEV_1, mouth pressure inhale, mouth pressure exhale, peak flow, peak cough flow
8. A DEXA scan may be performed at any visit, if deemed clinically necessary by the Investigator (i.e. rapid weight gain)
9. Prior to study medication administration
10. Adverse events to be obtained by weekly telephone call to parents by investigator / study nurse during off drug periods
11. Concomitant medication to be obtained by weekly telephone call to parents by investigator / study nurse during off drug periods

Table 10. Schedule of assessments for visits 110 – 133 (treatment beyond study period)

Subjects will not have to return to the hospital for laboratory safety testing during the 4-week treatment break UNLESS there is a medical / safety concern in which case the subject will be asked to return for further monitoring as determined by the investigator

Visit	110	111	112	113	114	115	116	117	118	119	120	121	122	123	124	125	126	127	128	129	130	131	132	133	Withdrawal
**Week**	w+97	w+98	w+99	w+100	w+101	w+102	w+103	w+104	w+105	w+106	w+107	w+108	w+109	w+110	w+111	w+112	w+113	w+114	w+115	w+116	w+117	w+118	w+119	w+120	
**Period**	Treatment beyond study																								
**Day of dosing**	Dosing occurs every 7 days +/- 1 day with reference to Visit 13 (start of extension)																								
**Study medication administration**[1]	x	x	x	x					x	x	x	x	x	x	x	x					x	x	x	x	
**Physical examination**												x												x	x
**Vital signs**[2]												x												x	x
**Weight, height**												x												x	
**ECG**				x					x			x				x					x			x	x
**Echocardiography**[3]																								x	x
**Urine**[4]		x		x					x	x		x		x		x					x	x		x	x
**Urine protein/creatinine**		x		x					x	x		x		x		x					x	x		x	x
**Urine α-1-microglobulin**				x					x			x				x					x			x	x
**Urine cystatin C**				x					x			x				x					x			x	x
**KIM-1**				x					x			x				x					x			x	x
**Safety biochemistry**				x					x			x				x					x			x	x
**Safety haematology**				x					x			x				x					x			x	x
**Blood smear for schistocytes**				x					x			x				x					x			x	x
**Thrombocyte count**[5]		x								x				x								x			
**Complement (C3)**				x					x			x				x					x			x	x
**MCP-1**				x					x			x				x					x			x	x
**Fibrinogen**				x					x			x				x					x			x	x
**Haptoglobin**				x					x			x				x					x			x	x
**CRP**				x					x			x				x					x			x	x
**aPTT/ PTT (INR)**				x					x			x				x					x			x	x
**Cystatin C**				x					x			x				x					x			x	x
**Pharmacokinetic sampling**[9]				x					x			x				x					x			x	x
**Dystrophin antibodies**																								x	
**Timed tests**[6]									x												x				x
**6-Minutes walk**									x												x				x
**Handheld myometry**									x												x				x
**Spirometry**[7]									x												x				x
**DEXA scan**[8]	x	x	x	x	x	x	x	x	x	x	x	x	x	x	x	x	x	x	x	x	x	x	x	x	x
**Adverse events**[10]	x	x	x	x	x	x	x	x	x	x	x	x	x	x	x	x	x	x	x	x	x	x	x	x	x
**Concomitant medication**[11]	x	x	x	x	x	x	x	x	x	x	x	x	x	x	x	x	x	x	x	x	x	x	x	x	x

1. Other assessments to be performed prior to study medication administration unless specified otherwise
2. Vital signs include temperature, blood pressure, pulse and respiratory rate

3. Parameters will be derived from M-mode measurements and include left ventricular ejection fraction, fractional shortening, left ventricular posterior wall thickness, left ventricular septum wall thickness, left ventricular end systolic diameter, left ventricular end diastolic diameter, and left ventricular ejection fraction modified Simpson's
4. Collect a first morning urine specimen
5. Via finger prick
6. Timed tests consist of: 10-meter walk/run, rising from floor, stair climb
7. Parameters include FVC, FEV_1, mouth pressure inhale, mouth pressure exhale, peak flow, peak cough flow
8. A DEXA scan may be performed at any visit, if deemed clinically necessary by the Investigator (i.e. rapid weight gain)
9. Prior to study medication administration
10. Adverse events to be obtained by weekly telephone call to parents by investigator / study nurse during off drug periods
11. Concomitant medication to be obtained by weekly telephone call to parents by investigator / study nurse during off drug periods

Table 11. Schedule of assessments for visits 134 - 157 (treatment beyond study period)

Subjects will not have to return to the hospital for laboratory safety testing during the 4-week treatment break UNLESS there is a medical / safety concern in which case the subject will be asked to return for further monitoring as determined by the investigator

**Visit**	**134**	**135**	**136**	**137**	**138**	**139**	**140**	**141**	**142**	**143**	**144**	**145**	**146**	**147**	**148**	**149**	**150**	**151**	**152**	**153**	**154**	**155**	**156**	**157**	**Withdrawal between visit 85 and 109**
**Week**	w+121	w+122	w+123	w+124	w+125	w+126	w+127	w+128	w+129	w+130	w+131	w+132	w+133	w+134	w+135	w+136	w+137	w+138	w+139	w+140	w+141	w+142	w+143	w+144	
**Period**	Treatment beyond study																								
**Day of dosing**	Dosing occurs every 7 days +/- 1 day with reference to Visit 13 (start of extension)																								
**Study medication administration**[1]	x	x	x	x					x	x	x	x	x	x	x	x					x	x	x	x	
**Physical examination**												x												x	x
**Vital signs**[2]												x												x	x
**Weight, height**												x												x	
**ECG**				x					x			x				x					x			x	x
**Echocardiography**[3]																								x	x
**Urine**[4]		x		x					x	x		x		x		x					x	x		x	x
**Urine protein/creatinine**		x		x					x	x		x		x		x					x	x		x	x
**Urine α-1-microglobulin**				x					x			x				x					x			x	x
**Urine cystatin C**				x					x			x				x					x			x	x
**KIM-1**				x					x			x				x					x			x	x
**Safety biochemistry**				x					x			x				x					x			x	x
**Safety haematology**				x					x			x				x					x			x	x
**Blood smear for schistocytes**				x					x			x				x					x			x	x
**Thrombocyte count**[5]		x								x				x								x			
**Complement (C3)**				x					x			x				x					x			x	x
**MCP-1**				x					x			x				x					x			x	x
**Fibrinogen**				x					x			x				x					x			x	x
**Haptoglobin**				x					x			x				x					x			x	x
**CRP**				x					x			x				x					x			x	x
**aPTT/ PTT (INR)**				x					x			x				x					x			x	x
**Cystatin C**				x					x			x				x					x			x	x
**Pharmacokinetic sampling**[9]				x					x			x				x					x			x	x
**Dystrophin antibodies**																								x	
**Timed tests**[6]									x												x				x
**6-Minutes walk**									x												x				x
**Handheld myometry**									x												x				x
**Spirometry**[7]									x												x				x
**DEXA scan**[8]	x	x	x	x	x	x	x	x	x	x	x	x	x	x	x	x	x	x	x	x	x	x	x	x	x
**Adverse events**[10]	x	x	x	x	x	x	x	x	x	x	x	x	x	x	x	x	x	x	x	x	x	x	x	x	x
**Concomitant medication**[11]	x	x	x	x	x	x	x	x	x	x	x	x	x	x	x	x	x	x	x	x	x	x	x	x	x

1. Other assessments to be performed prior to study medication administration unless specified otherwise
2. Vital signs include temperature, blood pressure, pulse and respiratory rate
3. Parameters will be derived from M-mode measurements and include left ventricular ejection fraction, fractional shortening, left ventricular posterior wall thickness, left ventricular septum wall thickness, left ventricular end systolic diameter, left ventricular end diastolic diameter, and left ventricular ejection fraction modified Simpson's
4. Collect a first morning urine specimen
5. Via finger prick
6. Timed tests consist of: 10-meter walk/run, rising from floor, stair climb
7. Parameters include FVC, FEV1, mouth pressure inhale, mouth pressure exhale, peak flow, peak cough flow
8. A DEXA scan may be performed at any visit, if deemed clinically necessary by the Investigator (i.e. rapid weight gain)
9. Prior to study medication administration
10. Adverse events to be obtained by weekly telephone call to parents by investigator / study nurse during off drug periods
11. Concomitant medication to be obtained by weekly telephone call to parents by investigator / study nurse during off drug periods

Table 12. Schedule of assessments for visits 158 – 181 (treatment beyond study period)

Subjects will not have to return to the hospital for laboratory safety testing during the 4-week treatment break UNLESS there is a medical / safety concern in which case the subject will be asked to return for further monitoring as determined by the investigator

**Visit**	**158**	**159**	**160**	**161**	**162**	**163**	**164**	**165**	**166**	**167**	**168**	**169**	**170**	**171**	**172**	**173**	**174**	**175**	**176**	**177**	**178**	**179**	**180**	**181**	**Withdrawal between visit 85 and 109**
**Week**	**w+145**	**w+146**	**w+147**	**w+148**	**w+149**	**w+150**	**w+151**	**w+152**	**w+153**	**w+154**	**w+155**	**w+156**	**w+157**	**w+158**	**w+159**	**w+160**	**w+161**	**w+162**	**w+163**	**w+164**	**w+165**	**w+166**	**w+167**	**w+168**	
**Period**	Treatment beyond study																								
**Day of dosing**	Dosing occurs every 7 days +/- 1 day with reference to Visit 13 (start of extension)																								
**Study medication administration[1]**	x	x	x	x					x	x	x	x	x	x	x	x					x	x	x	x	
**Weekly dosing option[12]**	x	x	x	x	x	x	x	x	x	x	x	x	x	x	x	x	x	x	x	x	x	x	x	x	
**Physical examination**												x												x	x
**Vital signs[2]**												x												x	x
**Weight, height**												x												x	
**ECG**				x					x			x				x					x			x	x
**Echocardiography[3]**																								x	x
**Urine[4]**		x		x					x	x		x		x		x					x	x		x	x
**Urine protein/creatinine**		x		x					x	x		x		x		x					x	x		x	x
**Urine α-1-microglobulin**				x					x			x				x					x			x	x
**Urine cystatin C**				x					x			x				x					x			x	x
**KIM-1**				x					x			x				x					x			x	x
**Safety biochemistry**				x					x			x				x					x			x	x
**Safety haematology**				x					x			x				x					x			x	x
**Blood smear for schistocytes**				x					x			x				x					x			x	x
**Thrombocyte count[5]**		x								x				x								x			
**Complement (C3)**				x					x			x				x					x			x	x
**MCP-1**				x					x			x				x					x			x	x
**Fibrinogen**				x					x			x				x					x			x	x
**Haptoglobin**				x					x			x				x					x			x	x
**CRP**				x					x			x				x					x			x	x
**aPTT/ PTT (INR)**				x					x			x				x					x			x	x
**Cystatin C**				x					x			x				x					x			x	x
**Pharmacokinetic sampling[9]**				x					x			x				x					x			x	x
**Dystrophin antibodies**																								x	
**Timed tests[6]**									x												x				x
**6-Minutes walk**									x												x				x
**Handheld myometry**									x												x				x
**Spirometry[7]**									x												x				x
**Parent questionnaire**																					x				
**DEXA scan[8]**	x	x	x	x	x	x	x	x	x	x	x	x	x	x	x	x	x	x	x	x	x	x	x	x	x
**Adverse events[10]**	x	x	x	x	x	x	x	x	x	x	x	x	x	x	x	x	x	x	x	x	x	x	x	x	x
**Concomitant medication[11]**	x	x	x	x	x	x	x	x	x	x	x	x	x	x	x	x	x	x	x	x	x	x	x	x	x

1. Other assessments to be performed prior to study medication administration unless specified otherwise

2. Vital signs include temperature, blood pressure, pulse and respiratory rate
3. Parameters will be derived from M-mode measurements and include left ventricular ejection fraction, fractional shortening, left ventricular posterior wall thickness, left ventricular septum wall thickness, left ventricular end systolic diameter, left ventricular end diastolic diameter, and left ventricular ejection fraction modified Simpson's
4. Collect a first morning urine specimen
5. Via finger prick
6. Timed tests consist of: 10-meter walk/run, rising from floor, stair climb
7. Parameters include FVC, FEV1, mouth pressure inhale, mouth pressure exhale, peak flow, peak cough flow
8. A DEXA scan may be performed at any visit, if deemed clinically necessary by the Investigator (i.e. rapid weight gain)
9. Prior to study medication administration
10. Adverse events to be obtained by weekly telephone call to parents by investigator / study nurse during off drug periods
11. Concomitant medication to be obtained by weekly telephone call to parents by investigator / study nurse during off drug periods
12. Weekly dosing: Decision to return to weekly dosing PRO051 will be made after full discussion between principal investigator and medical monitor

Table 13. Schedule of assessments for visits 182 – 205 (treatment beyond study period)

Subjects will not have to return to the hospital for laboratory safety testing during the 4-week treatment break UNLESS there is a medical / safety concern in which case the subject will be asked to return for further monitoring as determined by the investigator

**Visit**	**182**	**183**	**184**	**185**	**186**	**187**	**188**	**189**	**190**	**191**	**192**	**193**	**194**	**195**	**196**	**197**	**198**	**199**	**200**	**201**	**202**	**203**	**204**	**205**	**Withdrawal between visit 85 and 109**
**Week**	w+169	w+170	w+171	w+172	w+173	w+174	w+175	w+176	w+177	w+178	w+179	w+180	w+181	w+182	w+183	w+184	w+185	w+186	w+187	w+188	w+189	w+190	w+191	w+192	
**Period**	Treatment beyond study																								
**Day of dosing**	Dosing occurs every 7 days +/- 1 day with reference to Visit 13 (start of extension)																								
**Study medication administration**[1]	x	x	x	x					x	x	x	x	x	x	x	x					x	x	x	x	
**Weekly dosing option**[12]	x	x	x	x	x	x	x	x	x	x	x	x	x	x	x	x	x	x	x	x	x	x	x	x	
**Physical examination**												x												x	x
**Vital signs**[2]												x												x	x
**Weight, height**												x												x	
**ECG**				x					x			x				x					x			x	x
**Echocardiography**[3]																								x	x
**Urine**[4]		x		x					x	x		x		x		x					x	x		x	x
**Urine protein/creatinine**		x		x					x	x		x		x		x					x	x		x	x
**Urine α-1-microglobulin**				x					x			x				x					x			x	x
**Urine cystatin C**				x					x			x				x					x			x	x
**KIM-1**				x					x			x				x					x			x	x
**Safety biochemistry**				x					x			x				x					x			x	x
**Safety haematology**				x					x			x				x					x			x	x
**Blood smear for schistocytes**				x					x			x				x					x			x	x
**Thrombocyte count**[5]		x								x				x								x			
**Complement (C3)**				x					x			x				x					x			x	x
**MCP-1**				x					x			x				x					x			x	x
**Fibrinogen**				x					x			x				x					x			x	x
**Haptoglobin**				x					x			x				x					x			x	x
**CRP**				x					x			x				x					x			x	x
**aPTT/ PTT (INR)**				x					x			x				x					x			x	x
**Cystatin C**				x					x			x				x					x			x	x
**Pharmacokinetic sampling**[9]				x					x			x				x					x			x	x
**Dystrophin antibodies**																								x	
**Timed tests**[6]									x												x				x
**6-Minutes walk**									x												x				x
**Handheld myometry**									x												x				x
**Spirometry**[7]									x												x				x
**Parent questionnaire**																					x				
**DEXA scan**[8]	x	x	x	x	x	x	x	x	x	x	x	x	x	x	x	x	x	x	x	x	x	x	x	x	x
**Adverse events**[10]	x	x	x	x	x	x	x	x	x	x	x	x	x	x	x	x	x	x	x	x	x	x	x	x	x
**Concomitant medication**[11]	x	x	x	x	x	x	x	x	x	x	x	x	x	x	x	x	x	x	x	x	x	x	x	x	x

1. Other assessments to be performed prior to study medication administration unless specified otherwise

2. Vital signs include temperature, blood pressure, pulse and respiratory rate
3. Parameters will be derived from M-mode measurements and include left ventricular ejection fraction, fractional shortening, left ventricular posterior wall thickness, left ventricular septum wall thickness, left ventricular end systolic diameter, left ventricular end diastolic diameter, and left ventricular ejection fraction modified Simpson's
4. Collect a first morning urine specimen
5. Via finger prick
6. Timed tests consist of: 10-meter walk/run, rising from floor, stair climb
7. Parameters include FVC, FEV1, mouth pressure inhale, mouth pressure exhale, peak flow, peak cough flow
8. A DEXA scan may be performed at any visit, if deemed clinically necessary by the Investigator (i.e. rapid weight gain)
9. Prior to study medication administration
10. Adverse events to be obtained by weekly telephone call to parents by investigator / study nurse during off drug periods
11. Concomitant medication to be obtained by weekly telephone call to parents by investigator / study nurse during off drug periods
12. Weekly dosing: Decision to return to weekly dosing PRO051 will be made after full discussion between principal investigator and medical monitor

Table 14. Schedule of assessments for visits 206 – 229 (treatment beyond study period)

Subjects will not have to return to the hospital for laboratory safety testing during the 4-week treatment break UNLESS there is a medical / safety concern in which case the subject will be asked to return for further monitoring as determined by the investigator

**Visit**	**206**	**207**	**208**	**209**	**210**	**211**	**212**	**213**	**214**	**215**	**216**	**217**	**218**	**219**	**220**	**221**	**222**	**223**	**224**	**225**	**226**	**227**	**228**	**229**	**Withdrawal between visit 85 and 109**
**Week**	w+193	w+194	w+195	w+196	w+197	w+198	w+199	w+200	w+201	w+202	w+203	w+204	w+205	w+206	w+207	w+208	w+209	W+210	w+211	w+212	w+213	w+214	w+215	w+216	
**Period**	Treatment beyond study																								
**Day of dosing**	Dosing occurs every 7 days +/- 1 day with reference to Visit 13 (start of extension)																								
**Study medication administration**[1]	x	x	x	x					x	x	x	x	x	x	x	x					x	x	x	x	
**Weekly dosing option**[12]	x	x	x	x	x	x	x	x	x	x	x	x	x	x	x	x	x	x	x	x	x	x	x	x	
**Physical examination**												x												x	x
**Vital signs**[2]												x												x	x
**Weight, height**												x												x	
**ECG**				x					x			x				x					x			x	x
**Echocardiography**[3]																								x	x
**Urine**[4]		x		x					x	x		x		x		x					x	x		x	x
**Urine protein/creatinine**		x		x					x	x		x		x		x					x	x		x	x
**Urine α-1-microglobulin**				x					x			x				x					x			x	x
**Urine cystatin C**				x					x			x				x					x			x	x
**KIM-1**				x					x			x				x					x			x	x
**Safety biochemistry**				x					x			x				x					x			x	x
**Safety haematology**				x					x			x				x					x			x	x
**Blood smear for schistocytes**				x					x			x				x					x			x	x
**Thrombocyte count**[5]		x								x				x								x			
**Complement (C3)**				x					x			x				x					x			x	x
**MCP-1**				x					x			x				x					x			x	x
**Fibrinogen**				x					x			x				x					x			x	x
**Haptoglobin**				x					x			x				x					x			x	x
**CRP**				x					x			x				x					x			x	x
**aPTT/ PTT (INR)**				x					x			x				x					x			x	x
**Cystatin C**				x					x			x				x					x			x	x
**Pharmacokinetic sampling**[9]				x					x			x				x					x			x	x
**Dystrophin antibodies**																								x	
**Timed tests**[6]									x												x				x
**6-Minutes walk**									x												x				x
**Handheld myometry**									x												x				x
**Spirometry**[7]									x												x				x
**Parent questionnaire**																					x				
**DEXA scan**[8]	x	x	x	x	x	x	x	x	x	x	x	x	x	x	x	x	x	x	x	x	x	x	x	x	x
**Adverse events**[10]	x	x	x	x	x	x	x	x	x	x	x	x	x	x	x	x	x	x	x	x	x	x	x	x	x
**Concomitant medication**[11]	x	x	x	x	x	x	x	x	x	x	x	x	x	x	x	x	x	x	x	x	x	x	x	x	x

1. Other assessments to be performed prior to study medication administration unless specified otherwise

2. Vital signs include temperature, blood pressure, pulse and respiratory rate
3. Parameters will be derived from M-mode measurements and include left ventricular ejection fraction, fractional shortening, left ventricular posterior wall thickness, left ventricular septum wall thickness, left ventricular end systolic diameter, left ventricular end diastolic diameter, and left ventricular ejection fraction modified Simpson's
4. Collect a first morning urine specimen
5. Via finger prick
6. Timed tests consist of: 10-meter walk/run, rising from floor, stair climb
7. Parameters include FVC, FEV1, mouth pressure inhale, mouth pressure exhale, peak flow, peak cough flow
8. A DEXA scan will be scheduled x1 per year. However, a DEXA scan may be performed at any visit, if deemed clinically necessary by the Investigator (i.e. rapid weight gain).
9. Prior to study medication administration
10. Adverse events to be obtained by weekly telephone call to parents by investigator / study nurse during off drug periods
11. Concomitant medication to be obtained by weekly telephone call to parents by investigator / study nurse during off drug periods
12. Weekly dosing: Decision to return to weekly dosing PRO051 will be made after full discussion between principal investigator and medical monitor

Appendix 3 Parameters for routine hematology, biochemistry and urinalysis assessment

Hematology parameters

Hemoglobin
MCV
Erythrocyte count
Hematocrit
MCH (hemoglobin/erythrocyte count)
MCHC (hemoglobin/hematocrit)
Leukocyte count
Leukocyte differential count:
- Basophils
- Eosinophils
- Lymphocytes
- Monocytes
- Neutrophils

Reticulocyte countThrombocyte count
Blood smear for schistocytes

Coagulation parameters (blood)

Activated partial thromboplastin time (aPTT)
PTT (INR)

Biochemistry parameters (blood)

Sodium
Potassium
Calcium
Urea
Creatinine
Aspartate aminotransferase (ASAT)
Alanine aminotransferase (ALAT)
Gamma glutamyl transferase (γ-GT)
Lactate dehydrogenase (LDH)
Alkaline phosphatase (ALP)
Creatinine kinase (CK)
Glutamate dehydrogenase (GLDH)
Bilirubin
Amylases
Total protein
Albumin
Albumin/globulin ratio
Glucose
Cholesterol

MCP-1 (Monocyte chemotactic protein-1)

Cystatin-C

Urinalysis [a]

Glucose
Albumin
Protein
Creatinine

Every 4 weeks include;
α1-microglobulin

Cystatin C

Kidney injury molecule-1 (KIM-1)

Protein/creatinine ratio

Microscopy of urine sediment for erythrocytes, leukocytes and casts

[a]: All parameters are to be measured in the first morning specimen after 8 hours of not drinking

Appendix 4 Analytical Methods

I. Patient Screening

Skin biopsy – *in vitro* dystrophin mRNA

Skin biopsies taken from the patients during the screening period will be stored and transported in cell proliferation culture medium at ambient temperature. Fibroblasts will be isolated, preferably within 48 hours after sampling, by enzymatic dissociation of the skin tissue. After a series of proliferation cycles, approximately $2x10^6$ fibroblasts per ampoule will be stored in culture freezing medium under liquid nitrogen as backup. For the screening assay, proliferating fibroblasts will be plated in a 6-wells dish and converted into differentiating muscle-like cells by infection with an adenoviral vector expressing the MyoD gene (Ad5fib50 MyoD[26]). These MyoD-converted fibroblasts, now expressing dystrophin, will be transfected with 0, 100 and 250 nM PRO051, in duplo, per dish. A duplo 6-wells dish will be similarly processed using cells from a subsequent cell passage. RNA will be isolated from each well, 24 to 48 hours after transfection, stored at -80 ºC, or directly analyzed by RT-PCR analysis using DMD-gene specific primers flanking the patients' mutations. The set of primers may thus differ per patient. All RT-PCR assessments will accordingly be performed in duplo. The duplo dish will be used for back up. Sequence analysis will be performed on isolated PCR products (in duplo per product) to confirm both the mutation and the exon 51 skipping (if occurring) on RNA level.

Peripheral blood mononuclear cells (PB-MNC) **– *in vitro* dystrophin mRNA (exploratory)**

Blood samples taken from the patients collected in EDTA tubes (ambient) will be layered on top of a HistoPaque gradient within 48 hrs after sampling. Upon centrifugation, four layers become visible. The second layer (from top to bottom) contains the mononuclear cells that will be collected, washed, and centrifuged again. The cell pellet will be resuspended in proliferation culturing medium, counted, and either frozen in culture freezing medium or directly processed for further analysis. For this analysis, $2\text{-}8x10^6$ cells per well will be plated in 6-wells plates and cultured for 3 hrs. The cells will be transfected with 0, 100 and/or 250 nM PRO051 (depending on the total amount of cells isolated), in duplo, per dish. RNA will be isolated 72 hrs after transfection, stored at -80 ºC, or directly analyzed by RT-PCR analysis using DMD-gene specific primers flanking the patients' mutations. The set of primers may thus differ per patient. All RT-PCR assessments will be performed in duplo. Due to the limited amount of cells that can be isolated from a blood sample another duplo dish will not be included. Sequence analysis will be performed on isolated PCR products (in duplo per product) to confirm both the mutation and the exon 51 skipping (if occurring) on RNA level.

Note: The assay's applicability and reproducibility for the indicated purposes is under consideration. Based on the results in this study the assay may replace the original method based on MyoD-conversion of patient-derived fibroblasts.

II. Study Sample Screening

mRNA production in muscle tissue

Muscle biopsies will be taken from the DMD patient at different study time points, to be stored at -80 ºC, and sectioned using a pre-cooled cryotome (approximately –25 ºC). Total muscle RNA will be isolated from a series of sections of 50 μm up to a total weight of 10 to15 mg of muscle tissue, and analyzed by semi-quantitative RT-PCR (as recently described[21]). Two different screening procedures will be performed:

1. RT-PCR analysis focusing on the area flanking the targeted exon 51 will be performed to detect specific exon 51 skipping in muscle. Depending on the patient's mutation different sets of DMD-gene specific RT and PCR primers will be used. The

relative skipping efficiencies will be assessed through semi-quantitative LabChip analysis of the PCR products. Sequence analysis will be performed on isolated PCR products to confirm specific exon 51 skipping. All assessments will be performed in triplo.

2. To ensure the sequence-specificity of PRO051, the splicing throughout the entire DMD transcript will be screened for each patient. Total muscle RNA (from one time point only) will be analyzed by RT-PCR with a series of primer sets spanning the DMD gene as described for, or modified from, the Protein Truncation Test[27].

Dystrophin expression in muscle tissue

The patient muscle biopsies (stored at -80 ºC) will be sectioned using a pre-cooled cryotome (approximately –25 ºC). Dystrophin expression will be assessed by 1) immunofluorescence analyses of cross-sections and by 2) western blot analyses of total protein extracts, similar to the procedures recently described in van Deutekom et al., 2007).

1. 8 μm cross-sections will be collected on glass slides (two per slide, 10 slides) for immunofluorescence analysis and detection of novel dystrophin expression at the myofiber membranes. The glass slides and remainder of biopsy (embedded in Tissue-Tek) will be stored at -80 ºC. The cross-sections will be acetone-fixed and hybridized to a series of primary antibodies, either raised against human dystrophin (MANDYS106 and NCL-DYS2), or against human control muscle proteins such as laminin-alpha2. After hybridization to specific alexa fluor secondary antibodies, an integrated Leica microscope, digital camera and software (LAS) system will be used for signal detection and semi-quantitative measurement of signal intensities. All assessments will be performed in triplo.
2. total muscle protein extracts will be isolated from a series of sections of 50 μm up to a total weight of 20-30 mg of muscle tissue, and analyzed by Western blot analysis to detect and confirm novel dystrophin expression. Different amounts of protein (10 to 100 μg) will be applied per patient. The western blots will be hybridized to primary antibodies against human dystrophin (NCL-DYS1 or MANDYS106) and a human control muscle protein such as dysferlin. An IRDye 800CW secondary antibody will be applied appropriate for signal detection by the Odyssey Infrared Imaging System. Signal intensities will be measured using supplementary Odyssey software. All assessments will be performed in triplo.

mRNA production in peripheral blood mononuclear cells

Before PRO051 dosing, blood samples will be taken from the DMD patient at specific study time points to explore the feasibility of monitoring exon 51 skipping in blood during and after PRO051 treatment. Blood samples collected in EDTA tubes (ambient) will be layered on top of a HistoPaque gradient within 24-48 hrs after sampling. Upon centrifugation, four layers become visible. The second layer (from top to bottom) contains the mononuclear cells that will be collected, washed, and centrifuged again. The cell pellet will be used to isolate RNA for RT-PCR analysis using DMD-gene specific primers flanking the patients' mutations. The set of primers may thus differ per patient, but all primer sets and sequences are described[10,28].

The RT-PCR conditions are specifically optimized to detect low levels of DMD transcripts and exon 51 skipping in non-muscle cells. All assessments will be performed in triplo. The relative skipping efficiencies will be assessed through semi-quantitative LabChip analysis of the PCR products. Sequence analysis will be performed on isolated PCR products to confirm specific exon 51 skipping.

PRO051 level in plasma or muscle tissue

This assay to determine the concentration of the 2'O-methyl phosphorothioate oligoribonucleotide PRO051 (h51AON23) is based on the binding of PRO051 to a template capture probe. After immobilization of the complex on solid phase, a digoxigenin (DIG)-

labeled signal probe (ligation probe) is ligated to the complex followed by binding of horseradish peroxidase (HRPO)-labeled anti-DIG detection antibodies. The amount of enzyme (=peroxidase) activity in the wells is determined by the conversion of 3,3',5,5'-tetramethylbenzidine (TMB)/H_2O_2 through absorbance at 450 nm, which is directly related to the amount of PRO051 in the assay. Calibration curves and dilutions are prepared in 10% control pooled human plasma in PBS. This assay has been GLP validated. Muscle samples will be measured according to the same assay after homogenation and incorporating a muscle matrix correction. This procedure has been applied successfully before with muscle tissue from mice and monkeys.

Dystrophin antibodies in blood

Diluted serum samples (derived from blood samples) will be hybridized to pre-made western blots containing a series of control, and human dystrophin-positive, protein extracts (15-30 μg) to detect putative IgM and/or IgG antibodies against the novel dystrophin. The assay is adapted from a method described[29]. The control protein extracts include those isolated from transgenic mouse muscle expressing the full length human dystrophin, and from patient-specific myotube cultures before and after PRO051 transfection (then expressing the novel epitope that arises from the splicing of non-adjacent exons on RNA level). From all patients a pre-treatment sample will be included in which no dystrophin antibodies are expected. As a positive control the blots will also be hybridized to a dystrophin antibody that will detect human dystrophin in the relevant samples. An anti-human IgG/IgM IRDye 800CW secondary antibody will be used appropriate for signal detection by the Odyssey Infrared Imaging System. Signal intensities will be measured using supplementary Odyssey software. All assessments will be performed in triplo. In case of a positive response, confirmation of specificity will be obtained by employing existing competing dystrophin antibodies.
