# Supplementary material for: Long-Term Efficacy, Safety, and Pharmacokinetics of Drisapersen in Duchenne Muscular Dystrophy: Results from an Open-Label Extension Study
Source: PLoS One. 2016 Sep 2;11(9):e0161955. doi: 10.1371/journal.pone.0161955 (PMC5010191; doi:10.1371/journal.pone.0161955)
Supplement: S1 Results — Overall compliance was calculated to be 92.1%, and the mean (range) dose was 5.93 (5.10–6.02) mg/kg. (DOCX) [file pone.0161955.s004.docx]

## S1 Results

### Subjects

Overall compliance was calculated to be 92.1%, and the mean (range) dose was 5.93 (5.10–6.02) mg/kg.
