# Supplementary material for: Long-Term Efficacy, Safety, and Pharmacokinetics of Drisapersen in Duchenne Muscular Dystrophy: Results from an Open-Label Extension Study
Source: PLoS One. 2016 Sep 2;11(9):e0161955. doi: 10.1371/journal.pone.0161955 (PMC5010191; doi:10.1371/journal.pone.0161955)
Supplement: S1 Table — Missing values replaced by zero for subjects who became unable to complete the test at later visits. aTwo subjects were not able to complete the 6MWD test at extension study baseline; two additional subjects did not complete the 6MWD test at week 60. 6MWD: six-minute walk distance; SD: standard deviation. (DOCX) [file pone.0161955.s005.docx]

## S1 Table. Actual distance walked and change from baseline in the 6MWD test for all subjects and by disease status at extension study baseline (intent-to-treat population): subjects able to complete the 6MWD test at extension study baseline.

|  |  | **Actual distance walked (meters)** | | **Change from extension study baseline in distance walked (meters)** | | **Change from original study baseline in distance walked (meters)** | |
| --- | --- | --- | --- | --- | --- | --- | --- |
|  | **Subjects (N=12)^a^** | **Mean (SD)** | **Median (range)** | **Mean (SD)** | **Median (range)** | **Mean (SD)** | **Median (range)** |
| **Original study baseline** | 10 | 401.8 (72.96) | 394 (300 to 545) | – | – | – | – |
| **Extension study baseline** | 10 | 383.9 (121.22) | 362 (243 to 647) | – | – | −17.9 (58.66) | −16 (−88 to 102) |
| **Week 12** | 10 | 419.1 (122.12) | 423 (237 to 675) | 35.2 (28.69) | 39 (−6 to 69) | 17.3 (60.97) | 32 (−72 to130) |
| **Week 24** | 10 | 420.7 (126.46) | 460 (184 to 644) | 36.8 (59.78) | 37 (−59 to 115) | 18.9 (69.26) | 30 (−116 to 110) |
| **Week 36** | 10 | 408.9 (140.29) | 439 (187 to 675) | 25.0 (53.84) | 21 (−56 to 114) | 7.1 (82.03) | 20 (−113 to 130) |
| **Week 48** | 10 | 412.5 (166.22) | 463 (146 to 688) | 28.6 (79.74) | 46 (−110 to 127) | 10.7 (110.31) | 19 (−184 to 143) |
| **Week 60** | 10 | 410.1 (195.42) | 483 (75 to 694) | 26.2 (114.99) | 62 (−185 to 147) | 8.3 (142.90) | 57 (−259 to 149) |
| **Week 72** | 10 | 381.6 (228.42) | 470 (0 to 688) | −2.3 (144.25) | 44 (−263 to 135) | −20.2 (173.85) | 49 (−337 to 143) |
| **Week 80** | 10 | 394.6 (228.68) | 463 (0 to 690) | 10.7 (156.23) | 56 (−263 to 183) | −7.2 (179.52) | 66 (−337 to 178) |
| **Week 93** | 10 | 395.0 (233.35) | 467 (0 to 700) | 11.1 (157.19) | 63 (−263 to 190) | −6.8 (184.23) | 57 (−337 to 185) |
| **Week 105** | 10 | 389.8 (224.76) | 475 (0 to 642) | 5.9 (160.83) | 63 (–263 to 169) | –12.0 (180.18) | 70.5 (–337 to 159) |
| **Week 117** | 10 | 396.8 (231.75) | 494 (0 to 695) | 12.9 (163.52) | 64 (–263 to 188) | –5.0 (186.05) | 84 (–337 to 183) |
| **Week 129** | 10 | 383.3 (233.40) | 478 (0 to 691) | -0.6 (164.75) | 59 (–263 to 192) | –18.5 (188.69) | 73 (–337 to 187) |
| **Week 141** | 10 | 375.7 (224.73) | 464 (0 to 651) | -8.2 (160.91) | 20 (–263 to 201) | –26.1 (182.02) | 57 (–337 to 196) |
| **Week 153** | 10 | 379.8 (227.88) | 463 (0 to 645) | –4.1 (167.87) | 10 (–263 to 180) | –22.0 (185.12) | 69 (–337 to 175) |
| **Week 165** | 10 | 360.9 (217.97) | 426 (0 to 630) | –23.0 (154.48) | 7 (–263 to 165) | –40.9 (173.18) | 39 (–337 to 160) |
| **Week 177** | 10 | 359.4 (219.75) | 454 (0 to 625) | –24.5 (160.79) | 8 (–263 to 163) | –42.4 (178.27) | 63 (–337 to 158) |
| **Subjects walking ≥330 m at extension study baseline (N=7)** | | | | | | | |
| **Original study baseline** | 7 | 429.4 (67.43) | 426 (345 to 545) | – | – | – | – |
| **Extension study baseline** | 7 | 435.1 (108.03) | 406 (340 to 647) | – | – | 5.7 (53.97) | −5 (−74 to 102) |
| **Week 12** | 7 | 477.0 (93.67) | 450 (400 to 675) | 41.9 (28.40) | 50 (−6 to 69) | 47.6 (42.48) | 39 (−9 to 130) |
| **Week 48** | 7 | 498.3 (94.19) | 485 (381 to 688) | 63.1 (47.24) | 79 (−15 to 127) | 68.9 (55.58) | 59 (11 to 143) |
| **Week 93** | 7 | 523.3 (92.35) | 524 (422 to 700) | 88.1 (82.67) | 95 (−67 to 190) | 93.9 (74.1) | 111 (−35 to 185) |
| **Week 105** | 7 | 511.6 (83.32) | 504 (385 to 642) | 76.4 (102.59) | 96 (–115 to 169) | 82.1 (81.09) | 97 (–83 to 159) |
| **Week 117** | 7 | 522.1 (92.63) | 520 (384 to 695) | 87.0 (100.37) | 128 (–116 to 188) | 92.7 (85.82) | 115 (–84 to 183) |
| **Week 129** | 7 | 511.7 (102.87) | 505 (350 to 691) | 76.6 (110.85) | 104 (–150 to 192) | 82.3 (97.91) | 93 (–118 to 187) |
| **Week 141** | 7 | 501.0 (88.06) | 511 (362 to 651) | 65.9 (110.86) | 109 (–138 to 201) | 71.6 (91.22) | 89 (–106 to 196) |
| **Week 153** | 7 | 505.7 (94.03) | 520 (347 to 645) | 70.6 (122.38) | 125 (–153 to 180) | 76.3 (95.97) | 106 (–121 to 175) |
| **Week 165** | 7 | 480.1 (95.60) | 505 (337 to 630) | 45.0 (112.65) | 42 (–163 to 165) | 50.7 (90.33) | 67 (–131 to 160) |
| **Week 177** | 7 | 480.4 (97.69) | 503 (302 to 625) | 45.3 (124.47) | 91 (–198 to 163) | 51.0 (101.24) | 79 (–166 to 158) |
| **Subjects walking <330 m at extension study baseline (N=5)** | | | | | | | |
| **Original study baseline** | 3 | 337.3 (37.50) | 337 (300 to 375) | – | – | – | – |
| **Extension study baseline** | 3 | 264.3 (22.03) | 263 (243 to 287) | – | – | −73.0 (15.52) | −74 (−88 to −57) |
| **Week 12** | 3 | 284.0 (40.95) | 303 (237 to 312) | 19.7 (27.68) | 16 (−6 to 49) | −53.3 (24.95) | −63 (−72 to −25) |
| **Week 48** | 3 | 212.3 (108.89) | 153 (146 to 338) | −52.0 (89.44) | −97 (−110 to 51) | −125.0 (77.67) | −154 (−184 to −37) |
| **Week 93** | 3 | 95.7 (165.70) | 0 (0 to 287) | −168.7 (146.41) | −243 (−263 to 0) | −241.7 (134.36) | −300 (−337 to −88) |
| **Week 105** | 3 | 105.7 (183.02) | 0 (0 to 317) | –158.7 (163.70) | –243 (–263 to 30) | –231.7 (151.53) | –300 (–337 to –58) |
| **Week 117** | 3 | 104.3 (180.71) | 0 (0 to 313) | –160.0 (161.39) | –243 (–263 to 26) | –233.0 (149.24) | –300 (–337 to –62) |
| **Week 129** | 3 | 83.7 (144.92) | 0 (0 to 251) | –180.7 (125.68) | –243 (–263 to –36) | –253.7 (113.81) | –300 (–337 to –124) |
| **Week 141** | 3 | 83.3 (144.34) | 0 (0 to 250) | –181.0 (125.11) | –243 (–263 to –37) | –254.0 (113.24) | –300 (–337 to –125) |
| **Week 153** | 3 | 86.0 (148.96) | 0 (0 to 258) | –178.3 (129.71) | –243 (–263 to –29) | –251.3 (117.80) | –300 (–337 to –117) |
| **Week 165** | 3 | 82.7 (143.18) | 0 (0 to 248) | –181.7 (123.96) | –243 (–263 to –39) | ­–254.7 (112.10) | –300 (–337 to –127) |
| **Week 177** | 3 | 77.0 (133.37) | 0 (0 to 231) | –187.3 (114.18) | –243 (–263 to –56) | –260.3 (102.43) | –300 (–337 to –144) |

Missing values replaced by zero for subjects who became unable to complete the test at later visits.

^a^Two subjects were not able to complete the 6MWD test at extension study baseline; two additional subjects did not complete the 6MWD test at week 60.

6MWD, six-minute walking distance; SD, standard deviation.
