# Supplementary material for: Long-Term Efficacy, Safety, and Pharmacokinetics of Drisapersen in Duchenne Muscular Dystrophy: Results from an Open-Label Extension Study
Source: PLoS One. 2016 Sep 2;11(9):e0161955. doi: 10.1371/journal.pone.0161955 (PMC5010191; doi:10.1371/journal.pone.0161955)
Supplement: S2 Table — FEV1: forced expiratory volume in 1 second; FVC: forced vital capacity; MEP: maximal expiratory pressure; MIP: maximal inspiratory pressure; PCF: peak cough flow; PF: peak flow; SD: standard deviation. (DOCX) [file pone.0161955.s006.docx]

## S2 Table. Summary of spirometry parameters over 177 weeks: absolute values and changes from extension study baseline (intent-to-treat population).

| **Parameters** | **Time points** | **Subjects (N=12)** | **Absolute values** | | **Change from extension study baseline** | |
| --- | --- | --- | --- | --- | --- | --- |
|  |  |  | **Mean (SD)** | **Median** | **Mean (SD)** | **Median** |
| **FVC (L)** | Extension study baseline | 12 | 1.673 (0.353) | 1.705 | – | – |
|  | Week 48 | 12 | 1.752 (0.299) | 1.840 | 0.079 (0.255) | 0.140 |
|  | Week 93 | 12 | 1.823 (0.283) | 1.795 | 0.150 (0.190) | 0.160 |
|  | Week 141 | 11 | 1.798 (0.271) | 1.680 | 0.053 (0.217) | 0.110 |
|  | Week 177 | 12 | 1.729 (0.288) | 1.740 | 0.057 (0.289) | 0.060 |
| **FVC %-predicted** | Extension study baseline | 12 | 102.2 (18.4) | 98.0 | – | – |
|  | Week 48 | 12 | 102.3 (22.8) | 103.5 | 0.2 (14.3) | 4.0 |
|  | Week 93 | 12 | 100.6 (21.6) | 104.0 | –1.6 (10.9) | –3.5 |
|  | Week 141 | 11 | 89.2 (18.9) | 87.0 | –13.4 (16.3) | –13.0 |
|  | Week 177 | 12 | 88.3 (21.1) | 87.0 | –13.9 (13.5) | –12.5 |
| **FEV_1_ (L)** | Extension study baseline | 12 | 1.517 (0.340) | 1.600 | – | – |
|  | Week 48 | 12 | 1.583 (0.317) | 1.620 | 0.066 (0.262) | 0.065 |
|  | Week 93 | 12 | 1.651 (0.283) | 1.595 | 0.134 (0.179) | 0.150 |
|  | Week 141 | 11 | 1.640 (0.239) | 1.540 | 0.052 (0.167) | 0.100 |
|  | Week 177 | 12 | 1.582 (0.301) | 1.545 | 0.065 (0.288) | 0.120 |
| **FEV_1_ %-predicted** | Extension study baseline | 12 | 103.3 (19.7) | 94.5 | – | – |
|  | Week 48 | 12 | 102.8 (26.6) | 108.0 | –0.5 (17.7) | –2.0 |
|  | Week 93 | 12 | 100.1 (22.1) | 105.5 | –3.2 (11.9) | –6.5 |
|  | Week 141 | 11 | 88.9 (18.4) | 82.0 | –15.1 (13.2) | –14.0 |
|  | Week 177 | 12 | 87.8 (21.5) | 86.0 | –15.4 (16.9) | –11.0 |
| **MIP (cm H_2_O)** | Extension study baseline | 12 | –47.8 (16.5) | –44.5 | – | – |
|  | Week 48 | 12 | ­–51.0 (15.9) | –45.0 | –3.3 (12.0) | –2.5 |
|  | Week 93 | 12 | –55.4 (18.4) | –47.5 | –7.7 (17.6) | –2.5 |
|  | Week 141 | 11 | –42.9 (17.1) | –40.0 | 5.5 (19.2) | 3.0 |
|  | Week 177 | 12 | –44.1 (22.6) | –38.0 | 3.7 (22.0) | -2.0 |
| **MIP %-predicted** | Extension study baseline | 12 | 60.8 (21.9) | 61.8 | – | – |
|  | Week 48 | 12 | 61.7 (20.9) | 56.7 | 0.9 (16.2) | –0.4 |
|  | Week 93 | 12 | 64.7 (24.5) | 58.7 | 3.8 (21.5) | –1.8 |
|  | Week 141 | 11 | 45.9 (21.6) | 42.8 | –13.9 (22.8) | –16.3 |
|  | Week 177 | 12 | 47.9 (28.5) | 38.7 | –12.9 (23.9) | –10.9 |
| **MEP (cm H_2_O)** | Extension study baseline | 12 | 56.6 (21.0) | 57.5 | – | – |
|  | Week 48 | 12 | 47.9 (13.2) | 45.0 | –8.7 (16.0) | ­–8.5 |
|  | Week 93 | 12 | 55.8 (21.6) | 52.5 | –0.8 (13.2) | –1.0 |
|  | Week 141 | 11 | 44.5 (13.7) | 42.0 | –12.7 (17.7) | –8.0 |
|  | Week 177 | 12 | 45.2 (20.1) | 40.5 | –11.4 (16.7) | –11.0 |
| **MEP %-predicted** | Extension study baseline | 12 | 59.1 (25.2) | 62.2 | – | – |
|  | Week 48 | 12 | 45.3 (13.9) | 43.6 | –13.8 (17.6) | –13.2 |
|  | Week 93 | 12 | 50.2 (22.2) | 47.7 | ­–9.0 (13.2) | –9.4 |
|  | Week 141 | 11 | 35.3 (13.3) | 33.7 | –21.5 (18.1) | –23.8 |
|  | Week 177 | 12 | 36.1 (18.5) | 35.7 | –23.1 (15.6) | –24.1 |
| **PF (L/min)** | Extension study baseline | 12 | 218.0 (51.0) | 203.5 | – | – |
|  | Week 48 | 12 | 230.9 (69.7) | 207.5 | 12.9 (54.0) | 4.0 |
|  | Week 93 | 12 | 244.1 (49.3) | 250.0 | 26.1 (44.7) | 33.5 |
|  | Week 141 | 11 | 259.4 (45.7) | 250.0 | 33.5 (34.2) | 29.0 |
|  | Week 177 | 12 | 227.4 (72.2) | 247.0 | 9.4 (73.1) | 42.0 |
| **PF %-predicted** | Extension study baseline | 12 | 99.1 (25.3) | 99.1 | – | – |
|  | Week 48 | 12 | 100.3 (31.8) | 95.4 | 1.2 (21.0) | –3.6 |
|  | Week 93 | 12 | 103.4 (24.0) | 103.0 | 4.3 (17.4) | 5.2 |
|  | Week 141 | 11 | 97.9 (21.3) | 97.4 | 2.7 (16.6) | 0.8 |
|  | Week 177 | 12 | 89.2 (33.6) | 94.2 | –9.8 (26.3) | –0.8 |
| **PCF (L/min)** | Extension study baseline | 12 | 168.3 (45.1) | 160.0 | – | – |
|  | Week 48 | 12 | 187.5 (49.2) | 195.0 | 19.2 (55.8) | 30.0 |
|  | Week 93 | 12 | 200.0 (35.7) | 200.0 | 31.7 (53.1) | 35.0 |
|  | Week 141 | 11 | 222.7 (48.4) | 250.0 | 50.0 (59.3) | 50.0 |
|  | Week 177 | 12 | 190.8 (77.4) | 200.0 | 22.5 (81.1) | 40.0 |

FEV_1_, forced expiratory volume in 1 second; FVC, forced vital capacity; MEP, maximal expiratory pressure; MIP, maximal inspiratory pressure; PCF, peak cough flow; PF, peak flow; SD, standard deviation.
