# Supplementary material for: Long-Term Efficacy, Safety, and Pharmacokinetics of Drisapersen in Duchenne Muscular Dystrophy: Results from an Open-Label Extension Study
Source: PLoS One. 2016 Sep 2;11(9):e0161955. doi: 10.1371/journal.pone.0161955 (PMC5010191; doi:10.1371/journal.pone.0161955)
Supplement: S3 Table — aIncludes all AEs (classified as both treatment related and unrelated) ocurring in at least two subjects in either the continuous or the intermittent treatment phases. bReported as an “injection-site reaction” with no specific description. cOnly two of these three subjects were described by the investigator as having ulcers; the third subject was reported as having wounds at the injection site, which the investigator has confirmed were not ulcers. AE: adverse events; MCP1: monocyte chemotactic protein-1. (DOCX) [file pone.0161955.s007.docx]

## S3 Table. AEs^a^ that occurred in at least two subjects during the 188-week extension phase.

| **System-organ class** | **Adverse events**  **N (%)** | **Continuous**  **treatment phase (up to week 72)**  **(N=12)** | **Treatment interruption and intermittent treatment phases**  **(after week 72)**  **(N=12)** |
| --- | --- | --- | --- |
| **Blood and lymphatic system disorders** | Thrombocytopenia | 4 (33) | 2 (17) |
|  | Leukopenia | 2 (17) | 1 (8) |
| **Gastrointestinal disorders** | Vomiting | 4 (33) | 8 (67) |
|  | Diarrhea | 3 (25) | 4 (33) |
|  | Upper abdominal pain | 2 (17) | 3 (25) |
|  | Constipation | 2 (17) | 2 (17) |
|  | Gastritis | 2 (17) | 0 |
|  | Nausea | 2 (17) | 4 (33) |
|  | Hemorrhoids | 0 | 2 (17) |
| **General disorders and administration-site conditions** | Injection-site induration | 12 (100) | 10 (83) |
|  | Injection-site erythema | 10 (83) | 7 (58) |
|  | Injection-site hematoma | 10 (83) | 7 (58) |
|  | Injection-site discoloration | 8 (67) | 8 (67) |
|  | Injection-site pain | 8 (67) | 7 (58) |
|  | Pyrexia | 8 (67) | 5 (42) |
|  | Fatigue | 3 (25) | 2 (17) |
|  | Influenza-like illness | 1 (8) | 2 (17) |
|  | Injection-site dryness | 3 (25) | 1 (8) |
|  | Injection-site inflammation | 3 (25) | 1 (8) |
|  | Injection-site irritation | 3 (25) | 0 |
|  | Injection-site pruritus | 3 (25) | 3 (25) |
|  | Injection-site reaction^b^ | 2 (17) | 1 (8) |
|  | Injection-site ulcer | 1 (8) | 3 (25)^c^ |
|  | Injection-site atrophy | 0 | 6 (50) |
|  | Chest pain | 0 | 2 (17) |
| **Infections and infestations** | Gastroenteritis | 7 (58) | 6 (50) |
|  | Nasopharyngitis | 6 (50) | 6 (50) |
|  | Rhinitis | 4 (33) | 3 (25) |
|  | Fungal infection | 3 (25) | 0 |
|  | Respiratory tract infection | 2 (17) | 1 (8) |
|  | Upper respiratory tract infection | 1 (8) | 6 (50) |
|  | Influenza | 1 (8) | 2 (17) |
|  | Otitis media | 1 (8) | 2 (17) |
|  | Virus infection | 1 (8) | 2 (17) |
|  | Injection-site infection | 0 | 2 (17) |
| **Injury, poisoning and procedural complications** | Joint injury | 0 | 2 (17) |
|  | Joint sprain | 0 | 2 (17) |
| **Investigations** | α_1_-microglobulin urine increase | 12 (100) | 7 (58) |
|  | Glutamate dehydrogenase increase | 6 (50) | 5 (42) |
|  | Cystatin C increase | 5 (42) | 8 (67) |
|  | Urinary sediment abnormal | 5 (42) | 3 (25) |
|  | γ-glutamyltransferase increase | 3 (25) | 2 (17) |
|  | Complement factor C3 decrease | 2 (17) | 2 (17) |
|  | Troponin I increase | 2 (17) | 0 |
|  | C-reactive protein increase | 0 | 3 (25) |
|  | Haptoglobin increase | 0 | 3 (25) |
|  | White blood cells urine-positive | 0 | 3 (25) |
| **Musculoskeletal and connective tissue disorders** | Pain in extremity | 3 (25) | 0 |
|  | Back pain | 2 (17) | 3 (25) |
|  | Myalgia | 2 (17) | 0 |
|  | Arthralgia | 1 (8) | 2 (17) |
| **Nervous system disorders** | Headache | 9 (75) | 7 (58) |
| **Renal and urinary disorders** | Proteinuria | 11 (92) | 6 (50) |
|  | Albuminuria | 7 (58) | 9 (75) |
| **Respiratory, thoracic, and mediastinal disorders** | Cough | 3 (25) | 4 (33) |
|  | Oropharyngeal pain | 3 (25) | 2 (17) |
| **Skin and subcutaneous tissue disorders** | Dry skin | 2 (17) | 0 |
|  | Alopecia | 0 | 2 (17) |
|  | Erythema | 0 | 2 (17) |
| **Not coded** | Elevated level of MCP1 | 2 (17) | 5 (42) |

^a^Includes all AEs (classified as both treatment related and unrelated) ocurring in at least two subjects in either the continuous or the intermittent treatment phases.

^b^Reported as an “injection-site reaction” with no specific description.

^c^Only two of these three subjects were described by the investigator as having ulcers; the third subject was reported as having wounds at the injection site, which the investigator has confirmed were not ulcers.

MCP1, monocyte chemotactic protein-1.
