# Supplementary material for: Long-Term Efficacy, Safety, and Pharmacokinetics of Drisapersen in Duchenne Muscular Dystrophy: Results from an Open-Label Extension Study
Source: PLoS One. 2016 Sep 2;11(9):e0161955. doi: 10.1371/journal.pone.0161955 (PMC5010191; doi:10.1371/journal.pone.0161955)
Supplement: S4 Table — Only data for scheduled visits at weeks 24, 48, 72, 96, 120, 144 and 168 are displayed in this table. (DOCX) [file pone.0161955.s008.docx]

## S4 Table. Echocardiography during continued treatment (safety population).

| **Subject** | **Visit** | **Fractional shortening, %** | **Left ventricular ejection fraction, %** | **Left ventricular ejection fraction modified Simpson’s, %** | **Left ventricular end diastolic diameter, %** | **Left ventricular end systolic diameter, %** | **Left ventricular posterior wall thickness, %** | **Left ventricular septum wall thickness, %** | **Clinically significant abnormal** |
| --- | --- | --- | --- | --- | --- | --- | --- | --- | --- |
| **1** | Week 24 | 37 | NA | NA | 4.0 | NA | 4.0 | 7.0 | No |
|  | Week 48 | 38 | 69 | 65 | 4.2 | 2.6 | 4.1 | 6.7 | No |
|  | Week 72 | 40 | 71 | 75 | 4.1 | 1.1 | 5.1 | 6.3 | No |
|  | Week 96 | 38 | 68 | 48 | 3.7 | 2.3 | 6.0 | 6.0 | No |
|  | Week 120 | 35 | 65 | 62 | 2.6 | 3.9 | 5.3 | 6.0 | No |
|  | Week 144 | 34 | 64 | 46 | 3.8 | 2.5 | 7.0 | 5.0 | No |
|  | Week 168 | 35 | 65 | 67 | 3.7 | 2.4 | 4.0 | 6.0 | No |
| **2** | Week 24 | 41 | NA | NA | 4.1 | NA | 7.0 | 6.0 | No |
|  | Week 48 | 44 | 76 | 52 | 4.0 | 2.2 | 7.0 | 5.0 | No |
|  | Week 72 | 38 | 69 | 78 | 3.8 | 2.4 | 5.6 | 6.9 | No |
|  | Week 96 | 31 | 59 | 49 | 4.2 | 2.9 | 7.0 | 8.0 | No |
|  | Week 120 | 42 | 73 | 74 | 3.8 | 2.2 | 7.0 | 6.0 | No |
|  | Week 144 | 37 | 68 | 51 | 4.0 | 2.5 | 7.0 | 7.0 | No |
|  | Week 168 | 43 | 75 | 70 | 3.7 | 1.1 | 7.0 | 7.0 | No |
| **3** | Week 24 | 25 | 49 | 52 | 4.1 | 3.1 | 5.0 | 8.0 | No |
|  | Week 48 | 33 | NA | 65 | 3.9 | 2.6 | 6.0 | 7.0 | No |
|  | Week 72 | 27 | 53 | 56 | 4.2 | 3.1 | 5.2 | 6.5 | No |
|  | Week 96 | 24 | 49 | NA | 4.1 | 3.0 | 5.0 | 6.0 | No |
|  | Week 120 | 33 | 62 | 60 | 4.1 | 2.7 | 5.5 | 7.8 | No |
|  | Week 144 | 31 | 59 | 68 | 3.9 | 2.7 | 5.9 | 7.0 | No |
|  | Week 168 | NA | NA | NA | NA | NA | NA | NA | No |
| **4** | Week 24 | 28 | NA | NA | 4.8 | NA | 8.0 | 9.0 | No |
|  | Week 48 | 28 | 53 | 63 | 4.8 | 3.5 | 7.0 | 9.0 | No |
|  | Week 72 | 28 | 54 | 45 | 4.4 | 3.2 | 10.0 | 9.6 | No |
|  | Week 96 | 35 | 64 | 61 | 4.8 | 3.1 | 7.0 | 7.0 | No |
|  | Week 120 | 32 | 61 | NA | 2.9 | 4.3 | 7.4 | 10.0 | No |
|  | Week 144 | 30 | 58 | NA | 4.0 | 2.8 | 8.0 | 10.0 | No |
|  | Week 168 | 37 | 57 | NA | 4.8 | 3.4 | 7.0 | 8.0 | No |
| **5** | Week 24 | 31 | NA | NA | 4.6 | NA | 5.0 | 6.0 | No |
|  | Week 48 | 33 | 61 | 57 | 4.9 | 3.4 | 4.3 | 6.7 | No |
|  | Week 72 | 33 | 58 | 49 | 4.6 | 3.1 | 6.1 | 6.1 | No |
|  | Week 96 | 38 | 68 | 54 | 4.7 | 2.9 | 5.0 | 5.0 | No |
|  | Week 120 | 26 | 52 | 50 | 4.6 | 3.3 | 5.0 | 5.5 | No |
|  | Week 144 | 32 | 61 | 49 | 4.0 | 2.7 | 7.0 | 6.0 | No |
|  | Week 168 | 36 | 68 | 68 | 4.3 | 2.8 | 5.0 | 5.0 | No |
| **6** | Week 24 | 31 | 59 | 54 | 3.9 | 2.7 | 6.0 | 6.0 | No |
|  | Week 48 | 36 | NA | 56 | 3.9 | 2.5 | 6.0 | 5.7 | No |
|  | Week 72 | 41 | 73 | 69 | 3.7 | 2.2 | 5.0 | 6.0 | No |
|  | Week 96 | 37 | 68 | 57 | 3.5 | 2.2 | 7.0 | 6.0 | No |
|  | Week 120 | 40 | 71 | 57 | 2.1 | 3.4 | 5.0 | 5.9 | No |
|  | Week 144 | NA | NA | NA | NA | NA | NA | NA | No |
|  | Week 168 | NA | NA | NA | NA | NA | NA | NA | No |
| **7** | Week 24 | 39 | NA | NA | 3.6 | NA | 7.0 | 9.0 | No |
|  | Week 48 | 36 | 66 | 59 | 4.0 | 2.6 | 7.3 | 7.7 | No |
|  | Week 72 | 40 | 71 | 50 | 4.0 | 2.4 | 6.5 | 7.4 | No |
|  | Week 96 | 37 | 68 | 61 | 3.8 | 2.4 | 7.2 | 7.2 | No |
|  | Week 120 | 41 | 73 | 73 | 2.3 | 3.9 | 7.4 | 7.4 | No |
|  | Week 144 | 36 | 67 | NA | 3.8 | 2.4 | 6.0 | 7.0 | No |
|  | Week 168 | 34 | 64 | 77 | 3.5 | 2.3 | 9.0 | 8.0 | No |
| **8** | Week 24 | 20 | 41 | 34 | 5.2 | 4.2 | 8.0 | 8.0 | No |
|  | Week 48 | 16 | 35 | 43 | 4.9 | 4.1 | 6.0 | 6.0 | No |
|  | Week 72 | 21 | 42 | 42 | 5.0 | 3.9 | 6.0 | 7.3 | No |
|  | Week 96 | 17 | 36 | 47 | 4.7 | 3.9 | 7.5 | 8.0 | No |
|  | Week 120 | 23 | 42 | 40 | 4.9 | 3.8 | 6.0 | 7.4 | Yes |
|  | Week 144 | NA | NA | NA | NA | NA | NA | NA | NA |
|  | Week 168 | NA | NA | NA | NA | NA | NA | NA | NA |
| **9** | Week 24 | 40 | 72 | 63 | 3.4 | 2.0 | 5.9 | 4.6 | No |
|  | Week 48 | 41 | NA | 57 | 3.5 | 2.1 | 5.3 | 5.6 | No |
|  | Week 72 | 29 | 57 | 55 | 3.1 | 2.2 | 5.5 | 6.8 | No |
|  | Week 96 | 32 | 61 | 62 | 3.3 | 2.2 | 7.0 | 6.0 | No |
|  | Week 120 | 35 | 66 | 66 | 3.4 | 2.2 | 4.8 | 4.8 | No |
|  | Week 144 | 44 | 76 | 67 | 3.5 | 2.0 | 5.3 | 4.9 | No |
|  | Week 168 | 40 | 71 | 65 | 3.5 | 2.1 | 5.5 | 5.6 | No |
| **10** | Week 24 | 33 | NA | NA | 4.3 | NA | 7.0 | 8.0 | No |
|  | Week 48 | 35 | 65 | NA | 4.0 | 2.6 | 6.0 | 6.0 | No |
|  | Week 72 | 33 | 63 | 42 | 3.6 | 2.4 | 8.2 | 7.1 | No |
|  | Week 96 | 37 | 67 | 60 | 3.9 | 2.4 | 7.0 | 5.0 | No |
|  | Week 120 | 34 | 64 | NA | 2.3 | 3.6 | 4.0 | 7.0 | No |
|  | Week 144 | 41 | 72 | 44 | 3.9 | 2.3 | 8.0 | 7.0 | No |
|  | Week 168 | 37 | 61 | 62 | 3.6 | 2.7 | 7.0 | 6.0 | No |
| **11** | Week 24 | 33 | NA | NA | 4.3 | NA | 6.0 | 7.0 | No |
|  | Week 48 | 32 | 61 | 48 | 4.0 | 2.8 | 5.7 | 5.5 | No |
|  | Week 72 | 38 | 80 | 48 | 3.4 | 2.1 | 8.0 | 7.0 | No |
|  | Week 96 | 43 | 75 | 45 | 3.1 | 1.7 | 8.0 | 6.0 | No |
|  | Week 120 | 35 | 66 | 54 | 3.7 | 2.4 | 6.0 | 7.0 | No |
|  | Week 144 | 44 | 76 | 55 | 3.8 | 2.1 | 7.0 | 7.0 | No |
|  | Week 168 | 37 | 67 | 45 | 3.8 | 2.4 | 7.0 | 6.0 | No |
| **12** | Week 24 | 34 | 64 | 60 | 4.1 | 2.7 | 6.0 | 7.0 | No |
|  | Week 48 | 32 | 61 | NA | 4.0 | 2.7 | 5.5 | 5.0 | No |
|  | Week 72 | 40 | 71 | 55 | 4.0 | 2.4 | 5.7 | 5.7 | No |
|  | Week 96 | 35 | 65 | 65 | 4.0 | 2.6 | 6.4 | 6.4 | No |
|  | Week 120 | 37 | 68 | 53 | 3.9 | 2.4 | 7.0 | 6.0 | No |
|  | Week 144 | NA | NA | NA | NA | NA | NA | NA | No |
|  | Week 168 | NA | NA | NA | NA | NA | NA | NA | No |

Only data for scheduled visits at weeks 24, 48, 72, 96, 120, 144 and 168 are displayed in this table
